# The Contribution of Type 2 Diabetes to Parkinson’s Disease Aetiology

**DOI:** 10.3390/ijms25084358

**Published:** 2024-04-15

**Authors:** Samo Ribarič

**Affiliations:** Institute of Pathophysiology, Faculty of Medicine, University of Ljubljana, Zaloška 4, 1000 Ljubljana, Slovenia; samo.ribaric@mf.uni-lj.si

**Keywords:** advanced glycation end products, diabetes, hyperglycemia, insulin resistance, neuroinflammation, Parkinson’s disease, reactive oxidative species (ROS), α-synuclein oligomers, α-synuclein

## Abstract

Type 2 diabetes (T2D) and Parkinson’s disease (PD) are chronic disorders that have a significant health impact on a global scale. Epidemiological, preclinical, and clinical research underpins the assumption that insulin resistance and chronic inflammation contribute to the overlapping aetiologies of T2D and PD. This narrative review summarises the recent evidence on the contribution of T2D to the initiation and progression of PD brain pathology. It also briefly discusses the rationale and potential of alternative pharmacological interventions for PD treatment.

## 1. Introduction

Type 2 diabetes (T2D) and Parkinson’s disease (PD) are chronic disorders that have a significant health impact on a global scale [1,2]. This narrative review aims to summarise the recent evidence on the contribution of T2D to PD aetiology. The introduction presents the roles of α-syn in physiological and PD-related conditions and describes the crucial PD-related changes in glucose metabolism. The following two chapters compare T2D risk factors for patients with PD and the clinical signs and symptoms of PD and T2D. A whole chapter is devoted to the overlap of PD and diabetes aetiologies and reviews the contribution of insulin resistance to the initiation and progression of PD pathology. The final chapter briefly discusses the rationale and potential of alternative pharmacological interventions for PD.

PD is the second most common chronic neurodegenerative brain disorder (NDD), with a progressive loss of sensory, locomotor, cognitive, and autonomic functions. It is distinguished from other NDDs by the concomitant loss of dopamine neurons in pars compacta of substanca nigra (SNpc) and the accumulation of aggregated α-syn containing amyloid fibrils, i.e., Lewy Bodies, and inclusion bodies in the cytoplasm of surviving neurons [3,4].

The α-syn protein monomer (α-syn), the precursor of aggregated α-syn forms, has many physiological functions and is present in different brain cells (e.g., dopaminergic and noradrenergic neurons, microglia, and astrocytes) and in several brain regions (e.g., the frontal cortex, hippocampus, striatum, and olfactory bulb). However, only the dopaminergic neurons of SNpc seem to have the increased vulnerability to oxidative stress that contributes to α-syn-associated pathology [5,6]. The unique developmental conditions and the morphological and electrophysiological properties of differentiated SNpc dopaminergic neurons underpin their vulnerability to oxidative stress compared to other dopaminergic cells. Ni and Ernst (2020) comprehensively reviewed these conditions and properties [7]. They can be summarised as (a) an increased vulnerability to electron transport inhibitors [8,9,10], (b) many synaptic contacts per neuron with a high energy rate consumption per nerve cell (estimated at 1 to 2.5 million synaptic connections per human SNpc neuron, about 10 times more than other subpopulations of dopaminergic neurons) [11], (c) a higher adenosine triphosphate (ATP) consumption rate due to the autonomous pacemaker action potential transmission [12,13,14], and (d) the high concentrations of transcription factors during embryogenesis, which program the undifferentiated, future prSNpc neurons to a high energy state [15,16].

The lack of established diagnostic markers hinders the detection and monitoring of PD progression since PD shares some features (e.g., neuroinflammation) with multiple system atrophy (MSA), vascular Parkinsonism (VaP), and dementia with Lewy Bodies (LBs) [17]. There are many monogenic types of familial PD, and some of them, for example, deficiencies in gene expressions of E3 ubiquitin ligase (Parkin), protein deglycase DJ-1 (DJ-1), and protein kinase with a mitochondrial targeting domain (PINK1), have been extensively studied [18,19,20]. However, most PD cases are sporadic, and their pathogenesis is poorly understood [21]. Ageing is a primary risk factor and is sometimes described as a pre-PD condition [22,23,24]. Also, epidemiological, preclinical, and clinical data support the association of T2D with an increased risk or accelerated progression of PD signs and symptoms [1,2,25,26,27,28].

### 1.1. Physiology and Pathophysiology of Alfa Synuclein

The hallmark of PD is the loss of dopaminergic neurons in the SNpc, accompanied by LBs in surviving brain neurons [3,4]. A significant component of LBs is α-syn, and this protein is also present in the blood, mainly in red blood cells (RBCs). Oligomeric α-syn (α-synO)-enriched extracellular vesicles from RBCs cross the blood–brain barrier (BBB), accumulate in astrocytes and microglia, and precipitate astrocytic death and microglial activation [29,30]. Therefore, PD is an α-syn proteinopathy associated with chronic systemic- and neuroinflammation. This inflammation is associated with increased levels of inflammatory cytokines with activated microglia in the central nervous system (CNS) and high levels of activated monocytes in the periphery. The chronic CNS and peripheral inflammations in PD are elicited and sustained by increased concentrations of toxic α-synO [31].

The propagation of α-syn proteinopathy in the CNS is facilitated by the protein’s release and uptake through interconnected neural networks, thus enabling pathological, oligomeric and fibrillar forms of α-syn aggregates to induce the endogenous, normal α-syn to adopt a self-propagating conformation that precipitates the formation of insoluble, phosphorylated, aggregates of LBs [32,33,34,35,36,37,38]. Therefore, the transmission of PD-associated pathology within the brain is caused by the prion-like properties of pathological α-syn forms that are not transmissible from one organism to another, in contrast to MSA α-syn proteinopathy [5].

#### Formation of α-syn Oligomers, Fibrillar Conglomerates, and LBs

α-syn is a physiological, soluble form of the protein, with three domains: (a) the C-terminal region, (b) the central—non-amyloid, hydrophobic—region enabling the protein’s oligomerisation, and (c) the alpha helix-forming N-terminal region that enables lipid binding.

The protein is located in most brain neurons, in their cytoplasm and organelles. It regulates the cells’ membrane and organelle-associated processes like (a) mitochondrial (MT) fusion–fission and the prevention of reactive oxidative species (ROS)-induced MT fragmentation, (b) limiting cytosolic protein import across the outer MT membrane to the intermembrane MT space by blocking the Voltage-Dependent Anion Channel and the translocase of the outer mitochondrial membrane (TOM), (c) promoting the protein with SNARE motif (SNARE) complex formation to facilitate exocytosis, and (d) interacting with clathrin to promote the formation of endocytotic vesicles [5,32].

The α-syn’s intraneuronal locations are the synaptic terminals, nucleus, MT, endoplasmic reticulum, and the endolysosomal system [5]. The overexpression of normal α-syn is sufficient to promote the formation of toxic α-synO and fibrillar conglomerates [5]. α-synO is assumed to form pores in the plasma membrane of cells [39,40], facilitating the diffusion of Ca^2+^, increasing its intracellular concentration, and triggering cell death [41,42,43]. Intracellular, toxic α-synO is secreted by an exosomal, calcium-dependent mechanism and is assumed to transmit PD pathology from cell to cell in the brain [44].

Toxic α-syn forms are associated with (a) increased oxidative stress [45,46], (b) impaired axonal transport [47,48,49], (c) the disruption of ubiquitin-proteasome machinery [50,51], (d) impaired MT and synaptic function [52,53], (e) the disruption of normal deoxyribonucleic acid (DNA) transcription by the inhibition of the histone deacetylase (HDAC) acetylation of DNA and DNA methylation by methyltransferase 1 [54,55], and (f) Golgi apparatus (GA) fragmentation associated with impaired GA Ca^2+^ transport and impaired GA protein trafficking and maturation [56,57]. The accumulation of undigestible α-syn forms in the endolysosomal system inhibits vesicular traffic between the endoplasmic reticulum (ER) and the GA [58]. Also, sustained overactivation of the ER’s unfolded protein response (UPR) precipitates apoptosis. Apoptosis is triggered by the release of ER’s Ca^2+^ stores into the low-Ca^2+^-concentration cytosol [59].

The ER’s other essential functions are also perturbed by toxic α-syn forms (e.g., protein and lipid synthesis, protein folding, post-translational modifications, ER-GA vesicular protein transport, and ER-MT signalling). PD-associated GA malfunction has detrimental cell-wide and transcellular effects since the GA processes and sorts proteins for multiple cellular destinations, including lysosomes, plasma membranes, and secretory vesicles [5]. Also, α-syn binds to other proteins, e.g., tau protein and β-amyloid, thus forming LBs, pathological, neuronal intracytoplasmic inclusions of over 70 proteins with core α-syn protein fibrillar aggregates [60,61,62].

Brain insulin resistance (IR) attenuates the insulin-degrading enzyme’s (IDE) inhibition of α-syn fibril formation from α-synO, thus increasing the probability of the development of PD pathology [63,64,65].

### 1.2. Brain Glucose Metabolism

Normal brain nerves’ ATP needs depend on a continuous supply and oxidative phosphorylation of glucose [66,67]. The regional brain ATP synthesis rate rapidly adapts to meet local energy demands; the average time from uptake to complete glucose oxidation is less than 1 ms. Nerve signal transduction-related activities account for 70% of the total brain energy consumption, and 50% of the whole brain energy consumption is used by the Na^+^-K^+^-ATPase activity [67,68]. Excitatory neurons have a significantly larger energy consumption than inhibitory neurons, about 80% and 20%, respectively. In the brain, glucose transporters (GLUT) GLUT3 and GLUT4 transport glucose into neurons; GLUT1 transports glucose into astrocytes and endothelial cells [69,70,71].

In contrast to neurons, astrocytes rely on glycolysis to meet their energy demands [67,68,72]. Brain nerve cells can utilise lactate to meet energy demands. The primary source of lactate production is the high glycolysis rate in astrocytes, with lactate released into the extracellular space, which nerve cells acquire and oxidise. Astrocytes also regulate local cerebral blood flow to nerve cells by inducing vasoconstriction during low energy demands (i.e., low oxygen consumption) and vasodilatation when the nerves’ energy demands and oxygen consumption increase [73].

SNpc dopaminergic neurons have a very high energy consumption [7]. These neurons are also very susceptible to oxidative stress since the dopamine transporter pumps dopamine from the synaptic cleft back into the cytosol, and cytosolic dopamine generates increased levels of ROS, including dopamine semiquinone radicals [9,74].

The four significant pathways that metabolise glucose in brain cells are (a) glycolysis (metabolises glucose to pyruvic acid and generates 2 ATP molecules per one glucose), (b) glycogenesis (for glucose storage in astrocytes), (c) tricarboxylic acid cycle and oxidative phosphorylation (metabolises pyruvic acid to acetyl-CoA; acetyl-CoA is metabolised to carbon dioxide and water, and generates 30 or 32 molecules of ATP per one glucose), and (d) the pentose phosphate shunt pathway (metabolises glucose to ribose-5-phosphate and generates reduced nicotinamide adenine dinucleotide phosphate (NADPH) [67].

Brain glucose metabolism is impaired from the early stages of PD by many mechanisms, including insulin resistance, oxidative stress, abnormal glycated modifications, blood–brain barrier dysfunction, and hyperglycemia-induced damages. Over time, the combined effects of these pathological processes lead to excessive methylglyoxal (MGO) and ROS production, neuroinflammation, MT dysfunction with decreased energy availability, decreased dopamine neurotransmitter availability, brain neurotransmitter dysregulation, the aggregation and phosphorylation of α-synuclein, and dopaminergic neuron cell death [28].

Hyperglycemia is a non-motor symptom in more than 50% of patients with PD [75,76]. Hyperglycemia or diabetes (T2D and T1D) increases the risk of PD [77,78,79,80,81,82,83].

Patients in the early stages of PD have regional brain glucose hypometabolism, including the posterior temporoparietal, occipital, frontal, prefrontal, sensorimotor cortex and cerebellum putamen, pallidum, SNpc, and caudate [28,84,85,86,87,88]. The treatment of PD patients modifies brain glucose consumption. Levodopa decreases overall brain glucose consumption [89]. Deep brain stimulation of the subthalamus nuclei has diverse regional effects; it increases glucose metabolism in the pallidum, dorsolateral prefrontal cortex, and posterior parieto-occipital cortex and decreases glucose metabolism in the orbitofrontal cortex and parahippocampal gyrus [90]. Compared to PD patients without cognitive decline, who have a widespread cortical glucose hypometabolism, PD patients with mild cognitive impairment have reduced glucose metabolism in the temporoparietal region, and PD patients with dementia have a brain pattern of reduced glucose metabolism similar to demented patients with Alzheimer’s disease [91,92,93].

#### 1.2.1. Glycolysis

During glycolysis, each glucose molecule is converted to two pyruvate molecules and one nicotinamide adenine dinucleotide phosphate (NADPH) and four ATP molecules are generated. Enzymes regulate some glycolysis reactions, and some PD forms are associated with the dysregulation of glycolysis enzymes (e.g., phosphoglycerate kinase 1 (PGK1), pyruvate dehydrogenase, glyceraldehyde-3-phosphate dehydrogenase (GAPDH)).

**Figure 1:**
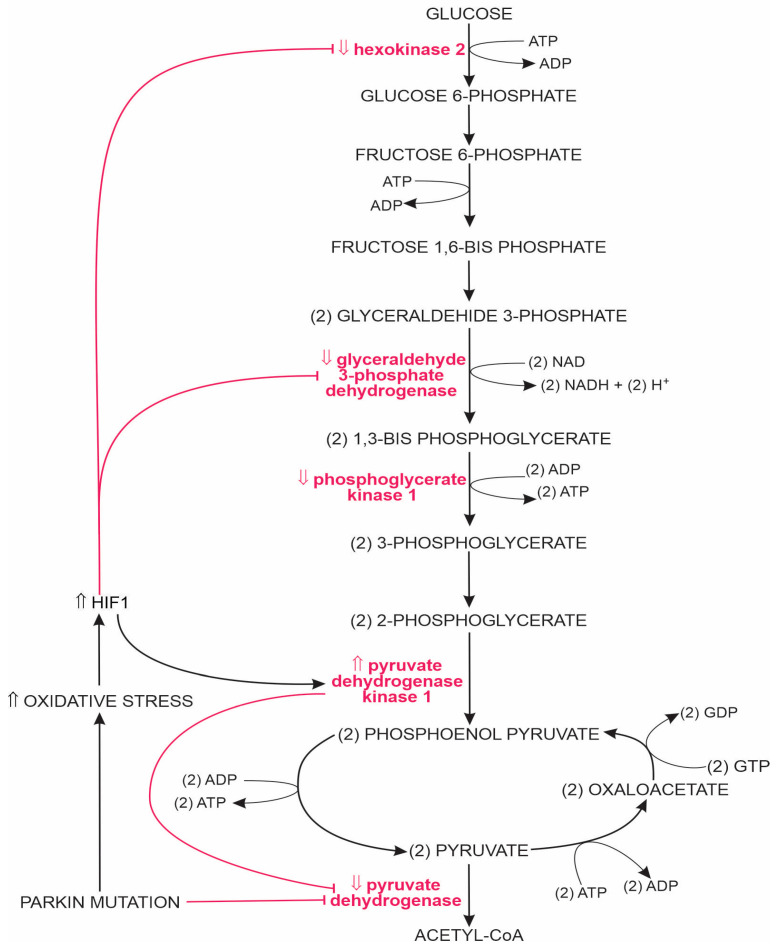
PD-associated changes in the activity of glycolysis-associated enzymes are marked in red lettering. Numbers in brackets denote the number of molecules. Abbreviations: ADP (adenosine diphosphate); ATP (adenosine triphosphate); GDP (guanosine diphosphate); GTP (guanosine-5′-triphosphate); HIF1 (hypoxia-inducible factor 1); NAD (nicotinamide adenine dinucleotide); NADH (reduced nicotinamide adenine dinucleotide).; ⇓ (reduced activity); ⇑ (increased activity).

For example, (a) mutation of the *PGK1* gene (with reduced expression of the PGK1 protein) will attenuate the production of glycolysis ATP [28,94,95,96,97,98], and (b) reduced expressions of the *DJ1* or *PINK1* genes impair respiratory complex (RC) III function, stimulate MT depolarisation and ROS production, and increase levels of hypoxia-inducible factor 1 (HIF1). Increased HIF1 increases pyruvate dehydrogenase kinase 1 concentration, which inhibits pyruvate dehydrogenase, reduces TCA activity and ATP production, and causes lactic acidosis due to the accumulation of pyruvate and lactate [28,99,100]. Long-term increased HIF1 activity sustains chronically increased GAPDH levels, thus increasing oxidised and aggregated GAPDH. Aggregated GAPDH molecules promote α-syn aggregation. Also, oxidised GAPDH has an increased binding affinity with monomeric and oligomeric forms of α-syn, leading to GAPDH aggregation and inactivation and the inhibition of glycolysis [28,101,102]. T2D accentuates the PD-related detrimental changes in nerve cell glycolysis since glycated α-syn has an increased binding affinity with GAPDH; thus, the PD-related deficiency of functional α-syn and GAPDH molecules in nerve brain cells is further accentuated [103]. Examples of PD-associated changes in the activity of glycolysis-associated enzymes are presented in Figure 1.

#### 1.2.2. The Tricarboxylic Acid Cycle in the MT Matrix

**Figure 2:**
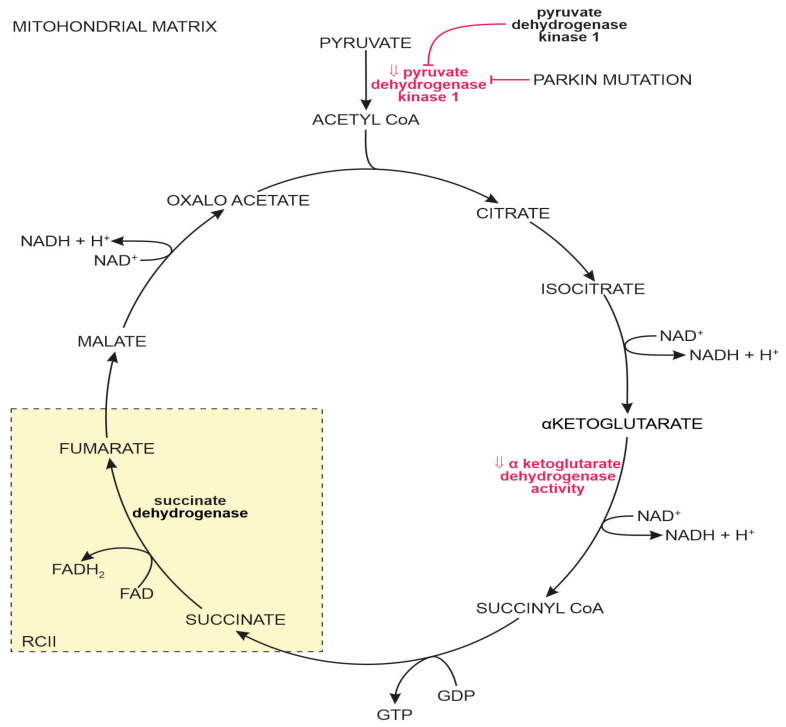
PD-associated reduced activity of the tricarboxylic acid cycle-associated enzymes is marked in red lettering. Abbreviations: FAD (flavin adenine dinucleotide); FADH_2_ (dihydroflavine-adenine dinucleotide); NAD (nicotinamide adenine dinucleotide); NADH (reduced nicotinamide adenine dinucleotide); RCII (respiratory complex 2 of the electron transport chain); ⇓ (reduced activity).

In mammalian cells’ MT, pyruvate molecules, the end product of glycolysis, are transported from the cytosol to the MT matrix. One pyruvate molecule is converted to one molecule of CO_2_, and a two-carbon acetyl group is attached to the coenzyme A (CoA), collectively called acetyl CoA. Acetyl CoA is the first and last step in the three citric acid closed-loop cycle. The eight-step TCA cycle produces two CO_2_ molecules, one ATP molecule (or an equivalent GTP molecule), and reduced forms of nicotinamide adenine dinucleotide (NAD^+^) and flavin adenine dinucleotide (FAD^+^) (i.e., NADH and FADH_2_). In the presence of oxygen, NADH and FADH_2_ transfer their electrons to the oxidative phosphorylation pathway. In PD, the activity of the α-ketoglutarate dehydrogenase enzyme complex (a compound of the TCA cycle) is attenuated in SNpc [104]; see Figure 2 for details.

#### 1.2.3. Oxidative Phosphorylation

Oxidative phosphorylation is implemented by respiratory complex proteins of the electron transport chain (RCI-IV) embedded in the inner MT membrane. PD animal models and human studies agree that inhibiting oxidative phosphorylation in nerve cells precipitates the degeneration of dopaminergic neurons and PD-associated symptoms. For example, the activity of RCC-I in idiopathic PD is reduced, and the accumulation of α-syn contributes to RCC-I inhibition, associated increased free radical production, and MT autophagy in SNpc [45,105,106,107,108,109].

In genetic forms of PD, oxidative phosphorylation in dopaminergic neurons is also degraded. For example, (a) reduced RC-I and RC-IV activity in Parkin mutations [110]; (b) attenuated MT ATP production associated with mutations of the α-syn protein expressing gene (SNCA) and oxidative inactivation of carbonic anhydrase, which interacts with MT amino acids [111,112]; and (c) attenuated RC II activity due to alpha–ketoglutarate dehydrogenase complex (KGDHC) deficiency with a lack of succinylation, an MT signalling pathway [104,107]. *PINK1* mutations are associated with attenuated RC I activity due to an increased open state of the MT permeability transition pore (MTPTP). Long-term opening of the MTPTP leads to the detrimental efflux of Ca^2+^ ions, antioxidant molecules (glutathione), and cytochrome c from the MT into the cytosol and the influx of small osmotically active molecules and water molecules promoting MT swelling. The loss of electron transport chain components (cytochrome c) stops electron transport and ATP production and, together with the loss of antioxidant molecules, reduces ROS MT buffering capacity and promotes ROS-elicited MT damage. Sustained MT cytochrome c leakage into the cytosol triggers apoptosis by activating pro-apoptotic signalling pathways. The sustained increase in the cytosolic Ca^2+^ concentration released from the MT also contributes to activating pro-apoptotic signalling pathways [113,114,115,116,117].

#### 1.2.4. The Pentose Phosphate Pathway

The cytosolic pentose phosphate pathway (PPP) is initiated after the first step of glycolysis, which converts glucose to glucose 6-phosphate in the presence of ATP and catalyses it with hexokinase. The PPP provides the cell with two metabolites that are vital for cell survival: ribose 5-phosphate (for nucleic acid synthesis) and NADPH. NADPH is essential for (a) the synthesis of fatty acids, sterols, nucleotides, and non-essential amino acids and (b) replenishing cellular antioxidant defences by reducing oxidised glutathione (GSSG) to reduced glutathione (GSH) [118].

The basal ganglia of patients in the early stages of PD have decreased levels of the PPP rate-limiting enzyme glucose-6-phosphate dehydrogenase (G6PD), which is responsible for regenerating NADPH from NADP to sustain cellular antioxidant defences [85]. On the other hand, in preclinical experiments, high glucose stimulation of the PPP leads to excessive NADPH, and increased NADPH levels could stimulate the unsaturated fatty acid production associated with α-syn aggregation in dopaminergic neurons. [85,119,120,121,122].

### 1.3. Insulin Resistance-Associated PD Pathology in Patients Comorbid with T2D

IR was investigated in two subgroups of PD patients (with and without dementia). Even after adjustment for disease duration and motor disability, the percentage of IR patients was significantly higher in PD patients with dementia; PD patients with dementia were two times more likely to have IR than patients with PD only [123].

A recent cohort study evaluated PD and T2D comorbidity for PD progression, neuropathological markers (α-syn quantification in key brain regions and the staging of vascular, Lewy, and Alzheimer’s pathologies), time to disability milestones (recurrent falls, wheelchair use, dementia, and care home placement), and survival. The researchers concluded that pre-existing T2D contributed to faster disease progression and reduced survival in PD patients, which was not associated with increased vascular, Lewy, or Alzheimer’s pathologies [124].

The potential of blood constituents and brain imaging for brain neuropathological markers in patients with PD and T2D comorbidity was evaluated by comparing the markers’ values in patients with PD only, with PD and T2D, and with T2D. The study’s conclusion was that the most significant risk markers for PD and T2D comorbidity were reduced low-density lipoprotein cholesterol (a measure of cerebral atrophy and increased dementia risk) and an increased fibrinogen concentration (a measure of CNS inflammation and lesion in the microcirculation, BBB, and neurovascular units) [125].

A paired comparison study evaluated the changes in neuropathology markers and clinical progression between (a) patients with PD only and PD patients with T2D comorbidity and (b) T2D-only patients and patients without PD or T2D over a 36-month follow-up period. Patients with PD and T2D comorbidity had worse motor disability signs, lower striatal dopamine transporter binding, and higher tau cerebrospinal fluid (CSF) levels than patients with PD only. Patients with T2D only had lower striatal dopamine transporter binding and higher tau and α-syn CSF levels than healthy controls. The overall conclusion was that T2D predisposes patients to PD pathology and that T2D and PD comorbidity accelerate PD progression [126].

## 2. Diabetes Risk Factors for PD

PD and T2D are age-associated chronic disorders; in the age group of 65 and above, about 25% of adults have T2D [127,128] and 1–2% have PD [129,130].

A recent retrospective study compared two cohorts, with prediabetes and T2D patients (without antidiabetic drugs or previous T2D diagnosis), to a reference cohort. The T2D and prediabetes cohorts were associated with a higher risk of PD. Stratification analysis by sex suggested prediabetes association with PD risk in women only. In stratification analysis below 65 years of age, T2D and prediabetes were associated with a greater PD risk in women and men [77].

In another recent, combined retrospective and prospective study, the effect of T2D and antidiabetic treatment was evaluated on the age at PD onset and the all-cause mortality. The calculated T2D standardised ratio for PD patients was 3.8% compared to a 5,3% overall prevalence in the general population. The study reported that (a) when T2D is treated with any antidiabetic therapy before PD diagnosis, the onset of PD is delayed, and (b) the duration of T2D increases mortality in patients who developed T2D before PD onset but not in patients who developed T2D after PD onset [131].

There are no known familial mutations in PD-associated genes in patients with comorbid T2D. However, PED/PEA-15 protein overexpression in animal models leads to concomitant T2D and PD pathology [132].

About 60% of PD patients have IR [133]. Phosphorylated α-synuclein deposits were detected in the cytoplasm of pancreatic β cells in most subjects with PD or PD-free patients with T2D [133]. Several large-scale cohort studies concluded that T2D patients have an increased causal risk of developing PD [1,82]. Diabetes-free patients with PD have an increased risk for IR [134]. Increased glycated haemoglobin concentration (HbA1c ≥ 42 mmol/mol) is an independent predictor for the rapid progression of motor symptoms in patients with PD [135,136,137]. Hyperglycemia promotes the formation of advanced glycated end products (AGEs), and these products accelerate α-syn aggregation by enabling the crosslinking of misfolded α-syn from monomers to dimers, α-synO, and β-sheets with fibril formation [1,138].

On the other hand, antidiabetic medication for patients with diabetes reduces the risk of developing Parkinson’s disease motor symptoms [1,83,139,140]. A recent observational cross-sectional study of 111 participants reported a positive association between IR and non-motor disability scores in patients with PD [141]. Prediabetes or midlife glycemic variability also increases the risk for PD [77,80,142,143].

The risk of PD is the highest for T2D patient subgroups between the ages of 24 and 44 and patients with a low body mass index with diabetes longer than five years [81,83]. Compared to patients with PD only, patients with T2D and PD had a more rapid progression of motor signs, such as postural instability, gait impairment, or wheelchair use, and a more rapid progression of cognitive symptoms, such as depression, mild cognitive impairment, dementia or memory loss, and a further reduced survival [124,135,136,144,145,146,147,148,149,150]. In patients with PD, the motor and non-motor PD markers were accelerated by concomitant T2D even after adjustment for age, sex, and baseline PD disease severity [126].

A recent study [151] analysed the effect of combined T2D and PD disorders on motor and non-motor signs. It concluded that motor and non-motor signs progress in patients with PD and T2D comorbidity. The study’s results are summarised in Table 1.

## 3. Clinical Signs and Symptoms of PD and Diabetes Are Either Similar or Distinct

### 3.1. The Order of Appearance of Gastrointestinal, Cognitive, and Motor Symptoms in PD

Cognitive and gastrointestinal (GI) PD symptoms often precede the motor symptoms. For example, delayed gastric emptying, constipation, or bowel incontinence precede the appearance of motor symptoms by years [1,152,153]. A recent analysis of the longitudinal relationship between the severity of GI (e.g., oropharyngeal dysphagia, delayed gastric emptying, constipation or bowel incontinence) and cognitive impairment symptoms (e.g., forgetfulness, slowed mental processing, decreased ability to multitask, and limited working memory) concluded that cognitive outcomes could be predicted by the severity of GI symptoms in newly diagnosed PD patients [154]. The time of appearance of cognitive impairment in PD is variable. However, about 80% of PD patients develop these symptoms between 5 and 20 years after diagnosis. Also, the pattern of cognitive impairment in PD patients ranges from posterior-cortical or frontal-executive to a mixed pattern of cognitive impairment [154,155,156,157,158].

The mammalian GI system is part of the gut–brain bidirectional communication system between the central and the enteric nervous system, connecting brain emotional and cognitive networks with peripheral intestinal function. This communication system (by vagal nerve signalling, the modulation of neuroimmune responses, and the release of neuroactive products from gut bacteria or enteroendocrine cells into the blood) enables the brain to influence intestinal activities, including the activity of immune cells and the gut, to affect human cognition and emotional activity [159,160,161]. Animal and human studies reported that changes in gut microbiome composition, associated with markers of chronic GI inflammation (e.g., tumour necrosis factor-α, interferon-gamma, interleukin-6, and interleukin-1β), accelerate the pathogenesis of PD by promoting neuroinflammation leading to neurodegeneration [152,162,163,164,165,166,167,168,169]. In humans, GI α-syn aggregates precede the onset of motor symptoms by years [152].

Animal and human studies support the hypothesis that α-syn aggregates initially develop in the enteric nervous system and are transported via the vagus nerve to the brain, where α-syn aggregates spread in a prion-like fashion by inducing the normal α-syn to adopt a self-propagating conformation that precipitates the formation of insoluble, phosphorylated aggregates [152,153].

### 3.2. Symptoms of PD and Diabetes

GI symptoms in PD and T2D overlap; for example, delayed gastric emptying is prevalent in 70% of patients with PD and 50% of patients with T2D. Constipation is also a frequent symptom in patients with PD and T2D [1]. Patients with T2D develop more severe motor (i.e., postural instability, gait disturbance) and non-motor symptoms (i.e., depression, cognitive impairment) than T2D-free PD patients [144,150,170].

### 3.3. Brain Pathology

Diabetes, in patients with PD, accelerates white matter atrophy in the parietal and occipital regions, frontal grey matter loss, and the progression of cognitive impairment [171,172]. Although diabetes increases the risk for cerebrovascular disease, neither postmortem studies nor brain magnetic resonance imaging studies have found any evidence for an increased burden of cerebrovascular disease among PD patients with concomitant T2D, compared to PD patients without T2D [2,124,144]. The BBB’s physiological permeability is compromised in patients with PD. The BBB’s pathological permeability is reflected in the presence of erythrocytes and serum proteins in the CSF, a reduced CSF/plasma albumin ratio, hemosiderin deposits around the brain capillaries, and capillary endothelial degeneration and dysfunction [173,174]. PD-associated pathological BBB changes are further accentuated by hyperglycemia (associated with BBB glycation, oxidative stress, and inflammation) that (a) decreases the density of pericytes, (b) prevents physiological interactions between astrocyte projections and endothelial cells, (c) disrupts tight junctions between endothelial cells and pericyte function, and increases the transport of extracellular vesicles with α-syn from the blood to the brain, thus further stimulating microglia inflammation [30,175,176,177,178,179,180,181,182,183].

### 3.4. Selective Loss of High-Metabolism Cells in T2D and PD

There is a selective loss of high-metabolic-rate cells in PD and T2D, i.e., insulin-releasing pancreatic islets’ β-cells in patients with diabetes and SNpc dopaminergic neurons with well-branched unmyelinated axons with tonic action potential activity, that releases neurotransmitters across a large number of brain synapses in PD patients. Clinical signs and symptoms (i.e., hyperglycemia in T2D and motor symptoms in PD) are preceded by at least a 50% loss of the respective high-metabolic cells [184,185,186,187,188,189].

## 4. Overlap of PD and Diabetes Aetiologies

### 4.1. T2D Does Not Accelerate Lewy Body Formation in PD

Although patients with concomitant T2D and PD have a consistently more rapid progression of motor signs and non-motor, cognitive symptoms [124,135,136,144,145,146,147,148,149,150], this accelerated PD progression is not reflected in an increased burden of LBs or a high global/regional vascular pathology compared to T2D-free PD patients [2,124,190]. There are at least two non-exclusive explanations for this contradiction: T2D degrades motor and non-motor brain functions by PD-independent mechanisms, and/or T2D increases the fraction of toxic α-synO that is not routinely measured on human histopathological brain sections [191]. α-synO is also localised to PD-affected brain regions free of LBs [191].

### 4.2. Amylin Neuropathology

PD and T2D are chronic disorders with systemic and CNS deposition of a misfolded protein, namely α-syn in PD and amyloid deposits in T2D [192]. T2D amyloid deposition is concentrated in the pancreatic islets (amylin is co-secreted with insulin in the pancreatic β-cells). However, amylin is also present in the blood and crosses the BBB into the brain’s intracellular and extracellular space, where amylin seems to accelerate α-syn aggregation [141,193,194,195,196,197,198,199,200,201]. Patients with PD have higher fasting plasma amylin/insulin ratios than healthy controls. The increased fasting plasma amylin/insulin ratios in PD patients could promote the accelerated deposition of the misfolded α-syn protein in the presence of amylin observed in vitro in these patients [196].

### 4.3. Hyperglycemia

Animal studies reported that hyperglycemia attenuates the action potential frequency of dopaminergic neurons and basal dopamine concentrations in the mesocorticolimbic and nigrostriatal motor pathways [202,203,204,205]. These results are consistent with the clinically observed, more severe PD phenotypes and higher L-Dopa doses in patients with concomitant PD and T2D [146,150].

As reviewed by Dai et al. [28], hyperglycemia contributes to PD pathophysiology by the following mechanisms: (a) dopamine synthesis, release, and uptake are inhibited in SNpc; (b) microglial stimulation promotes intense neuroinflammation; (c) autophagy, mitophagy, and the modulation of lysosomal function by late endosomal/lysosomal P5-type transport ATPase (ATP13A2) are inhibited; (d) there is a reduction in Parkin/PINK1 expression that inhibits MT function; and (e) the increased production of MGO (a very reactive dicarbonyl by-product of glucose metabolism) promotes glutamatergic hyperactivity and glycated modifications of proteins, DNA, RNA, and lipids.

The overall effects of hyperglycemia-related changes are as follows: (a) increased oxidative stress [206,207,208], (b) the accelerated aggregation of α-syn [209,210], (c) MT failure [207,211], (d) proteostasis failure, [209,210,212,213,214], and (e) dopaminergic neurons’ cell death that precipitates PD clinical non-motor and motor symptoms [28,182,183,215,216,217,218].

### 4.4. Increased Protein, Lipid, and Nucleic Acid Glycation

MGO levels are increased in PD and lead to enhanced glycated modifications of proteins, including α-syn crosslinking and phosphorylation in SNpc and locus coeruleus [219,220]. Also, MGO attenuates the removal of glycated α-syn forms by degrading the efficiency of the ubiquitin–proteasome system, the autophagy–lysosome pathway, and heat-shock proteins’ responses, thus promoting α-syn oligomerisation and aggregation, increased α-syn insolubility, and inclusion formation [209,210,212,213,214]. Patients with PD are more susceptible to the detrimental effects of glycation due to reduced nigral neuronal glutathione levels and age-associated decline in the glyoxalase [221,222].

Glycated and aggregated α-syn cannot bind to small unilamellar vesicles involved in synaptic exocytosis. Therefore, normal exocytosis of dopamine-rich vesicles at the presynaptic membrane is blocked with a concomitant presynaptic accumulation of ubiquitination and proteasomal degradation-resistant α-syn pathologic forms. This accumulation enhances ROS production and increases oxidative stress, accompanied by dopaminergic neurons’ cell death and the development of motor and non-motor PD symptoms [209,210,215,216,219,220,223].

As with PD, the nonenzymatic reactions (i.e., glycations) between MGO or glucose on the one hand and proteins, lipids, or nucleic acids on the other are accelerated in T2D due to hyperglycemia and increased levels of MGO. The glycation process generates a wide variety of AGEs identified in human SNpc and locus coeruleus tissue and is associated with accelerated cognitive ageing [209,219,220,224,225,226,227]. AGEs bind with receptors for advanced glycation end products (RAGEs). This activates the nuclear factor kappa-light-chain-enhancer of activated B cells (NF-κB) signalling pathway and promotes inflammation and neuronal death [228].

It has been suggested that T2D generates increased quantities of glycated α-syn species that (a) have a reduced ability to bind to intracellular membranes, (b) are resistant to ubiquitination and proteasomal degradation, and (c) are prone to aggregation into proteasomal and lysosomal degradation-resistant toxic α-synO forms [210,229]. Glycated α-synuclein species can be extracted from red blood cells and are potentially a biomarker to monitor the progression of PD [2,135].

### 4.5. Insulin Resistance

Insulin sustains the normal cell function of brain cells. The hormone binds to the cells’ insulin receptors, activating four signalling pathways via the insulin receptor substrates (IRS1) or IRS2 subtypes. These IRS-associated signalling pathways are (a) the Ca^2+^ channel and NMDA glutamate receptor activation pathway [1,192], (b) the GABA receptor recruitment promoting pathway [1,192,230], (c) the cell proliferation and synapse maintenance supporting MAPK signalling pathway [1,231,232], and (d) the PI3K (phosphatidylinositol 3-kinase)-AKT (protein kinase B) signalling pathway that regulates neurotransmitter release and promotes cell survival. Cell survival effects of the PI3K-AKT signalling pathway are promoted by (a) inhibiting excessive apoptosis and (b) attenuating the pro-inflammatory NF.κB-dependent signalling pathways, (c) attenuating FOXO1-mediated MT oxidative stress, (d) reducing α-syn aggregation by preventing the GSK3β inhibition of IDEs that break down the α-syn protein, and (e) promoting synapse regeneration by mTORC1/2 activation [1,233,234]. All four signalling pathways support neuronal growth and survival [2,192,235,236,237].

IR develops when insulin receptor-rich target cells develop a reduced response to high physiological insulin levels. The most reliable marker of systemic IR is the combination of elevated plasma glucose and insulin levels, reflecting the inability of pancreatic β cells to sustain plasma glucose within physiological levels. However, insulin regulates many cell functions in various cells, such as neuronal growth and survival. Therefore, the impact of IR on a specific function in a particular cell type cannot be measured by hyperglycemia alone.

Animal and human studies of IR in the skeletal muscle, liver, adipose, and brain tissue reported several causes of IR, including the downregulation of insulin receptors, ER stress, increased intracellular ROS, and chronic inflammation. These can modulate the insulin signalling pathways [28,192,238].

The current consensus is that the T2D-linked systemic IR and the brain IR—expressed together with systemic IR or existing independently—contribute to the initiation and progression of PD-associated α-syn brain pathology. Systemic IR effects that underpin PD-associated α-syn pathology are hyperglycemia and hyperglycemia-associated brain pathologies (e.g., small arteries; arteriole, venule, and capillary dysfunction; chronic neuroinflammation; and BBB dysfunction) [28,192]. Brain IR contributes to α-syn brain pathology by promoting α-syn aggregation and deposition and attenuating α-syn species clearance by IDEs, autophagy, and unfolded protein response [2,192].

Animal models of PD—elicited with environmental toxins (e.g., 1-methyl-4-phenyl-1,2,3,6-tetrahydropyridine (MPTP), 6-hydroxydopamine (6-OHDA)), nutrient excess, or a high-fat diet—were associated with central and peripheral IR and inflammation in combination with the accelerated loss of brain dopaminergic neurons [26]. More than half of PD patients have IR, and these patients have a more rapid progression of non-motor (i.e., dementia) and motor PD symptoms [28]. Chronic IR contributes to neuronal cell death by downregulating insulin receptor expression in (a) dopaminergic neurons of the basal ganglia and tegmental brain stem and (b) in the blood–brain barrier, thus reducing insulin transport to brain cells [28].

The metabolic effects of insulin on brain cells are mediated by the insulin receptor substrate 1 (IRS-1)–AKT and MAPK pathways [2,231,239,240,241]. Insulin indirectly affects glucose transport into brain cells by stimulating GLUT3 translocation to the brain cell’s membrane. Glucose uptake follows N-methyl-D aspartate receptor depolarisation, which mediates the sugar’s uptake by GLUT 3 [242,243,244].

Insufficient insulin actions on brain cells (i.e., brain IR) have profound metabolic changes in neurons, astrocytes, and microglia [245]. In astrocytes, insulin promotes glycogen storage and neurotrophic changes. Insulin modulates the release of cytokines from microglia and astrocytes [2,246,247,248]. Studies of brain IR in animal models and postmortem human tissue linked brain IR with increased IRS-1 phosphorylation at serine residues [249,250,251,252]. Brain IR in PD can develop without T2D and is assumed to be due to an exceptional, excessive age-related loss of insulin receptors in these patients [2,253,254,255]. Research on PD animal models concluded that IR attenuates MT biogenesis (i.e., the capacity of enzymes for glycolysis and oxidative phosphorylation), concomitantly preventing the MT metabolic capacity to meet the cells’ need for ATP and increasing intracellular ROS content and oxidative stress [256,257,258].

The joint effects of the PD and T2D pathological signalling pathways are as follows: (a) increased levels of pro-inflammatory cytokines, TNF-α, IL-1β, and IL-6, that enhance oxidative stress and MT dysfunction [1,259,260,261,262,263,264,265,266,267,268,269,270,271], (b) impaired neuroplasticity and memory formation [1,272,273,274], and (c) the development and acceleration of neurodegenerative disorders [1,147,272,273,274].

**Figure 3:**
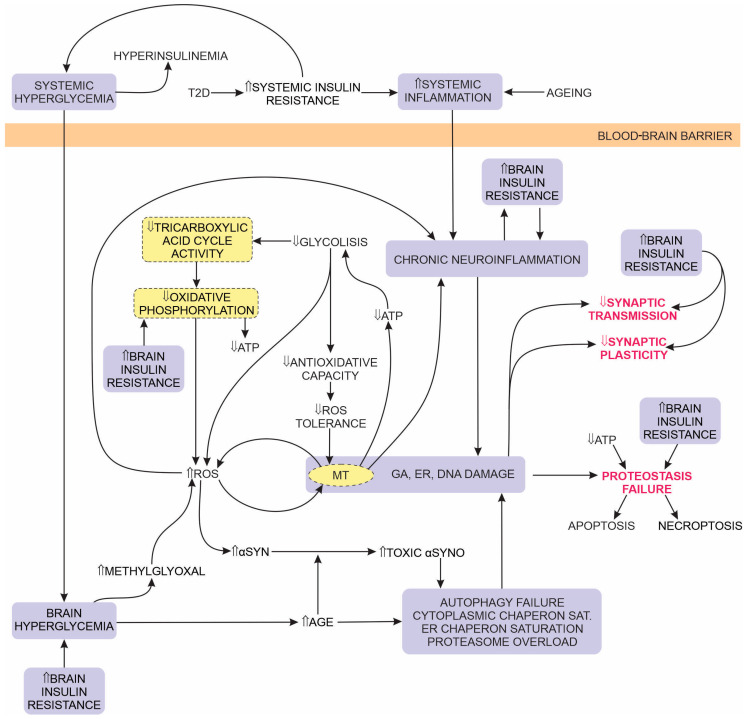
Combined effects of IR and PD-associated metabolic changes on neuron proteostasis. Abbreviations: AGE (advanced glycation end product); ATP (adenosine triphosphate)**;** DNA (deoxyribonucleic acid); ER (endoplasmic reticulum); GA (Golgi apparatus); MT (mitochondria); ROS (reactive oxidative species); T2D (Type 2 diabetes); α-syn (α-synuclein); α-synO (α-synuclein oligomer); ⇓ (reduced activity); ⇑ (increased activity). The yellow background indicates the MT location of metabolic processes.

Pro-inflammatory cytokines (e.g., TNF-α) downregulate IRS-1 expression, thus forming a positive feedback mechanism for accelerating IR by attenuating the intracellular insulin signalling [275]. Figure 3 summarises the combined effects of IR and PD-associated metabolic changes on neuron proteostasis.

### 4.6. Oxidative Stress and Inflammation

Dopaminergic neurons have a high metabolic rate that increases their susceptibility to oxidative stress-related cell structural and functional disruption [180]. For example, oxidative stress (a) oxidates dopamine into toxic quinone and semiquinone and (b) attenuates glucocerebrosidase activity; both changes further increase ROS levels [276,277]. Increased oxidative stress promotes α-syn oxidation, aggregation, and prion-like spread in the brain [277,278,279].

Long-term hyperglycemia modulates oxidative stress-related signalling pathways in brain cells in the following ways: (a) it upregulates the oxidative stress-enhancing transcription factors nuclear factor erythroid 2-related factor (Nrf 2) and FOXO1 and downregulates an Nrf 2 inhibitor, Keap1, in the SN [206]; (b) it downregulates the level and antioxidant catalase activity in the caudate putamen [206] and increases the level of thioredoxin-binding protein (TXNIP), an endogenous inhibitor of intracellular ROS elimination in dopaminergic neurons [207].

Chronic inflammation, with increasing intensity over time, correlates with the progression of motor and non-motor PD symptoms [259,260,261,262]. C-reactive protein (CRP) has been recently suggested as a primary marker to predict the risk of and monitor PD progression due to its use in widespread, standardised assays with a wide detection range [25]. However, human studies did not causally associate CRP levels with the pathogenesis of PD [280].

PD and T2D share chronic, low-grade inflammation as a common pathophysiological mechanism with increased levels of serum CRP, TNF-α, interferon-γ, interleukin-1β (IL-1β), IL-2, and IL-6, [83,263,267]. In brain IR, insulin receptors on astrocytes and microglia are downregulated, and the lack of insulin signalling promotes the increased secretion of inflammatory cytokines IL-6 and IL-8 from these cells [269,281]. Animal studies reported that increased microglial activation promotes (a) the excessive synaptic pruning of hippocampal nerve cells with associated memory impairment and (b) a reduced microglial clearance of extracellular α-synuclein, which promotes the formation of toxic α-synO forms and fibrils [270,271].

### 4.7. Mitochondrial Dysfunction

In PD, the high metabolic rate of brain dopaminergic neurons increases their vulnerability to the combined detrimental effects of increased MT oxidant stress and the MT accumulation of toxic α-synO. Brain IR further compounds the damaging impact of MT oxidant stress and toxic α-synO forms [2,28,235,237]. Insulin signalling supports normal MT biogenesis (i.e., oxidative function) via the modulation of the mTORC and FOXO1 signalling pathways, which converge and adjust the activity of peroxisome proliferator-activated receptor-γ coactivator 1-α (PGC1α). In summary, insulin stimulates MT ATP production in neurons and glial cells, and IR inhibition has the opposite effect [282,283,284].

Animal studies of T2D’s effects on brain cells reported a decreased ATP-to-adenosine diphosphate ratio, reduced MT membrane potential and coenzyme Q9 levels, increased Ca^2+^ accumulation in MT, and increased oxidative stress. The PD-related changes in MT biogenesis, including (a) a reduction in MT complex I content in the SN and (b) reduced levels of Parkin associated with the accumulation of a Parkin-interacting substrate that downregulates PGC1, are also present in animal models of advanced T2D stages [27,285,286,287,288,289,290,291].

PD and T2D share increased intracellular levels of MGO, a reactive dicarbonyl by-product of glucose metabolism (derived from glyceraldehyde 3-phosphate) that forms glycation products with proteins, lipids, and nucleic acids. Under physiological conditions, low levels of MGO are sustained by its conversion to D-lactate in the presence of the glyoxalase-1 enzyme. The enzyme’s efficiency is reduced by sustained hypoxia, inflammation, and oxidative stress, leading to increased toxic levels of MGO. Toxic MGO levels accelerate the production of glycation products that promote IR, increased inflammation, increased oxidative stress, the disruption of MT biogenesis, and cell death [218,292]. In animal models and human studies of PD, as well as in animal models of T2D, increased levels of MGO were associated with increased levels of the toxic dopamine-derived tetrahydroisoquinoline (ADTIQ), which promotes MT apoptosis and cell death [218,292].

### 4.8. Reduced Efficiency of Autophagy and Proteasome Degradation

PD inhibits autolysosome cargo degradation and retrograde trafficking, and T2D-associated IR and hyperglycemia inhibit autophagosome synthesis; both disorders reduce the efficiency of autophagy. Therefore, T2D in patients with PD further degrades the proteostasis of these patients [28,237,293,294,295,296,297,298,299]. Regular insulin signalling balances autophagy activity within physiological limits by (a) AKT-mediated activation of the mTOR and the inhibition of the FOXO1/3 signalling pathways and (b) preventing α-syn fibril formation from α-synO with the phosphatidylinositol 3-kinase (PI3K)-signalling pathway-activated IDEs. Normal IDE activity prevents α-syn fibril formation, thus enabling optimal autolysosome cargo degradation [63,64]. The physiological activity of the insulin-PI3K-AKT-(mTORC1) signalling pathway inhibits excessive autophagy and promotes nerve cell growth and survival [300,301,302]; therefore, early hyperinsulinemia associated with excessive insulin signalling will attenuate cell growth and reduce survival.

PD and T2D are associated with chronic inflammation in the brain that, via enhanced activation of the TNFα-IFNy signalling pathways, interferes with the Parkin function in proteasome degradation. Parkin (an E3 ubiquitin ligase) recognises and labels proteins located on the outer membrane of MT with ubiquitin (i.e., protein ubiquitination), thus directing them towards proteasomal or autophagy degradation. Regular Parkin activity is essential for cell survival; this protein promotes autophagy and proteasomal-associated removal of damaged MT and regulates MT-dependent and MT-independent apoptosis. PARK2 gene mutations are associated with reduced Parkin activity and familial PD [18,19,20]. Human studies and animal models of PD reported high expression levels of TNFα/IFNy in brain tissue, associated with increased expression levels of FAT10 (ubiquitin-like modifier HLA-F adjacent transcript 10 or ubiquitin D) [17,18].

In a recent experiment on TNFα/IFNγ-IFN-stimulated cells, FAT10 bound to Parkin to a degree that (a) overstimulated Parkin proteasome degradation, (b) inhibited Parkin-associated ubiquitination of the outer membrane of MT proteins, (c) delayed mitophagy, and (d) promoted cell death due to enhanced sensitivity to MT damage [18]. The study supports the hypothesis that chronic inflammation in PD, associated with high expression levels of TNFα/IFNγ from microglial cells in SNpc, stimulates excessively high expression levels of FAT10 and causally relates the inhibition of Parkin-regulated proteostasis and autophagy in dopaminergic brain nerve cells [18].

The expression of the Midnolin (MIDN) gene is reduced in some cases of sporadic, monogenic PD. In a cell model, the inhibition of MIDN expression reduced neurite outgrowth and the expression of Parkin. On the other hand, insulin promoted MIDN expression by the kinase1/2 and phosphoinositide 3-kinase-dependent signalling pathways [21]. Therefore, IR alone can attenuate MIDN expression and reduce proteasomal or autophagy degradation.

### 4.9. T2D Hyperglycemia Accelerates or Induces the Onset of PD Pathology

Hyperglycemia leads to degenerating PD-related brain pathways, especially the nigrostriatal motor pathway [205]. Several hyperglycemia-associated pathophysiology mechanisms in PD affect dopamine turnover, neuroinflammation, autophagy, lysosomal function and MT function, MGO production, and oxidative stress.

Dopamine synthesis, release, and uptake in dopaminergic neurons [303] is inhibited due to reduced activities of the dopamine transporter (DAT), G-protein-activated inward rectifier potassium channel 2, and vesicular monoamine transporter 2 (VMAT2) [206].

Hyperglycemia induces and sustains severe neuroinflammation by activating the microglia [304].

Normal autophagy and lysosomal cycling are inhibited by (a) increased levels of TXNIP [207,305] that activates ER stress-mediated nucleotide-binding oligomerisation domain (NOD)-like receptor protein-3 inflammasome complex formation and (b) by attenuated Parkin/PINK1 expression that inhibits normal MT function [207].

Hyperglycemia stimulates MGO production (a biologically reactive by-product of glycolysis), and increased MGO levels promote α-syn glycation, crosslinking, and phosphorylation in SNpc. Also, high MGO levels suppress the ubiquitin–proteasome system, the autophagy and lysosome pathways, and heat-shock proteins’ responses, thus inhibiting α-syn clearance and promoting α-syn oligomerisation, aggregation, and inclusion formation [209,210,214]. Glycated and aggregated α-syn cannot bind to small unilamellar vesicles, thus disrupting normal neurotransmission and eliciting dopaminergic neuron loss, ROS production, and the development or stimulated progression of motor and non-motor PD symptoms [209,210,215,216,223]. Methylglyoxal reacts with dopamine to produce the neurotoxin 1-acetyl-6, 7-dihydroxy-1,2,3,4-tetrahydro-isoquinoline, which promotes the apoptosis of dopaminergic neurons [218].

Hyperglycemia directly increases oxidative stress in the basal ganglia. It does this by first upregulating nuclear factor erythroid 2-related factor (Nrf 2) and forkhead box O1 (FOX O1) and downregulating Keap1 (Nrf 2 inhibitor) [206]. Second, it decreases the antioxidant activity of catalase [206] and increases the levels of the endogenous inhibitor for ROS elimination in dopaminergic neurons (TXNIP) [207]. Hyperglycemia also promotes oxidative stress in dopaminergic neurons indirectly by stimulating neuroinflammation [276,304], mitochondrial dysfunction [207], lysosomal dysfunction-related protein degradation impairment [207,276,305], VMAT2 reduction-related dopamine oxidation, and quinone production [206,208,276,306].

The aggregate effect of hyperglycemia-related impairments promotes the aggregation of α-syn and the apoptosis of the dopaminergic neurons field [217,304], reflected in motor deficits [304].

## 5. Pharmacological Interventions for PD

At present, no treatment reverses or stops the progression of PD. The lack of established diagnostic PD markers, such as distinguishing PD from other synucleinopathies, hinders drug development [17]. Current pharmacological treatment delays the progression of PD signs and symptoms. It is focused on enhancing dopaminergic transmission with three classes of drugs: (a) dopamine precursors (levodopa), (b) inhibitors (e.g., MAO B inhibitors) of enzymes that oxidise dopamine, and (c) agonists of dopamine receptors (e.g., ropinirole and pramipexole) [2,245,307,308].

### 5.1. Conventional Pharmacological Interventions for PD

The absence of disease-modifying treatments for PD was attributed to suboptimal preclinical testing and clinical trial protocols. Current animal models of PD focus on the late stage of the disease, i.e., the loss of dopaminergic neurons and motor dysfunction. These models are not optimal for studying the preclinical and early clinical signs and symptoms of PD (e.g., extra-dopaminergic and non-motor dysfunction) nor PD’s diverse aetiology. Also, current clinical trial protocols are not focused on drug development for the aetiology of diverse PD subgroups. Targeted preclinical testing of pharmacological treatment candidates is proposed to address individual derangements of PD pathology (e.g., iron and calcium overload, dysfunctions of the MT, organelles, mutant β-glucocerebrosidase and α-synuclein, chronic neuroinflammation, and disrupted autophagy) [309,310,311,312,313,314,315,316]. Protocols for washout, delayed, randomised withdrawal, and long-term trial designs were proposed to improve the detection of disease modification effects [317].

Conventional pharmacological PD treatment focuses on sustaining dopamine’s effects in the dopaminergic nerves’ depleted striatum by drugs that either (a) cross the BBB and metabolise to dopamine (levodopa), (b) bind to and activate dopamine receptors (dopamine agonists), (b) attenuate the breakdown of endogenous dopamine (monoamine oxidase B and catechol-O-methyl transferase inhibitors), and (d) attempt to correct the imbalance between dopamine and acetylcholine activity in the brain (anticholinergics) [318,319,320,321,322,323,324].

#### 5.1.1. Levodopa

Levodopa (L-DOPA) is metabolised in the brain to dopamine by DOPA decarboxylase [323]. It is most effective in reducing bradykinesia and enables PD patients to perform daily activities like dressing, walking, and handling utensils. Disease progression necessitates increasing the frequency of dosing and also increases the risk of significant adverse effects (i.e., dyskinesias and severe on–off motor fluctuations). Adverse effects due to the conversion of levodopa to dopamine outside the CNS can be attenuated by the concomitant use of carbidopa or benserazide peripheral inhibitors of DOPA decarboxylase that do not cross the BBB [323,325,326,327]. An alternative to oral levodopa is the intestinal infusion of levodopa gel [328].

#### 5.1.2. Dopamine Agonists

Dopamine agonists (e.g., ropinirole, rotigotine, and apomorphine) are prescribed as (a) initial therapy in younger PD patients, (b) to patients who cannot tolerate levodopa, or (c) as an adjunct to levodopa therapy to reduce the incidence and severity of dystonia, motor fluctuations, and dyskinesia in comparison to levodopa therapy alone [319,323,329]. The most significant adverse effects of treatment with dopamine agonists are (a) compulsive and impulsive behaviours (i.e., impulse control disorders) [330,331,332,333,334,335,336] and (b) withdrawal syndrome secondary to drug withdrawal or reduced dosage in patients with impulse control disorders [336].

#### 5.1.3. Monoamine Oxidase B (MAO-B) Inhibitors

MAO-B inhibitors (e.g., selegiline, rasagiline, and safinamide) are used for the initial treatment of PD patients to delay levodopa therapy with the inevitable levodopa-induced motor complications [329]. They sustain dopamine availability in the striatum by reducing dopamine’s monoamine oxidase B breakdown. The most common adverse effects of these drugs are gastrointestinal [323,329].

#### 5.1.4. Catechol-O-methyl Transferase (COMT) Inhibitors

COMT inhibitors (e.g., entacapone, tolcapone, opicapone) reduce the catechol-O-methyl transferase enzyme’s activity, thus reducing dopamine degradation in the brain. The drugs are used in combination with levodopa and may lead to the amplification of levodopa-induced adverse effects [323,324,329].

#### 5.1.5. Anticholinergics

Trihexyphenidyl, benztropine, orphenadrine, procyclidine, and biperiden are antagonists of cholinergic receptors that reduce acetylcholine neurotransmitter activity. Reduced brain acetylcholine neurotransmitter activity is assumed to compensate for the reduced PD-associated loss of dopaminergic brain activity [329]. Anticholinergics contribute to a modest reduction in rigidity and tremors and are prescribed to young PD patients at early stages of the disease. The drugs’ important adverse effect is confusion in elderly patients or patients with cognition disabilities [324].

#### 5.1.6. Amantadine

Amantadine is a weak antagonist of the NMDA-type glutamate receptor (has an anti-dyskinetic effect) that also increases dopamine release and blocks dopamine reuptake (has an antiparkinsonian effect) [337]. The drug is beneficial for attenuating the severity of levodopa-induced dyskinesias [338]. The drug’s side effects are hallucinations, confusion and impaired concentration, insomnia, nightmares, and agitation [324].

Physical activity reduces nigrostriatal LB formation and dopaminergic neuron death and increases brain-derived neurotrophic factor (BDNF) expression in animal models of PD [339,340,341]. The beneficial effects of moderate-to-vigorous physical activity, such as reduced PD risk and a slower decline in locomotor function, were reproduced in human studies [342,343,344,345,346]. Since T2D is a risk factor for PD, it is essential to note that sustaining a healthy diet, a normal BMI, and regular exercise reduces the risk for T2D [184,347,348,349].

### 5.2. Potential Alternative Pharmacological Interventions for PD

Preclinical, epidemiological, and post-mortem studies support the suggestions that T2D and PD have overlapping aetiologies, that T2D is a risk factor for PD, and that T2D accelerates the severity and progression of PD [2,28,235,350]. The shared disruptions of brain cells’ signalling pathways in T2D and PD are due to attenuated PI3K-AKT signalling pathway activity, which is the result of impaired insulin signalling, leading to (a) the FOXO1-promoted activation of pro-apoptosis factors and reduced MT function [2,235,237,351]; (b) GSK3-increased α-syn aggregation, NF-κB-enhanced NLRP3 inflammasome activation, pro-inflammatory cytokine signalling ((IL)-1β, -6; TNFα), and pro-inflammatory microglial activation [2,235,237]; and (c) reduced neural growth and synaptic plasticity or cell death due to mTOR inhibition [2,235,237].

Several drivers contributed to the development of alternative pharmacological PD treatments: (a) the overlap between PD and T2D aetiologies stimulated the evaluation of antidiabetic drugs for the treatment of PD [237,248,350,352,353], (b) the destructive effects of chronic, low-grade neuroinflammation in PD [245,354], (c) the overproduction of soluble α-syn promotes the formation of toxic α-synO–fibrillar conglomerates that increase the risk for PD [5,355], and (d) the high cholesterol and cholesterol metabolites accelerate α-syn aggregation, inhibit tyrosine hydroxylase expression, and reduce dopamine synthesis [356,357,358,359,360,361,362].

#### 5.2.1. Anti-Alfa Synuclein Vaccination and Humanised α-Synuclein Antibodies (Arbo et al. 2022) [245]

Neuroinflammation in PD can be attenuated by the injection of humanised α-synuclein antibodies or vaccination with peptides that elicit an Ab response against α-synuclein. Both approaches could be suitable for early-stage PD patients and populations at a high risk for PD [245]. The advantage of vaccination against α-synuclein over the injection of antibodies is the ability to treat a large population in a shorter time since frequent injections are not required [363]. The proof of principle for vaccination against PD was demonstrated in animal models and early clinical trials [363].

An example of a vaccine candidate for PD treatment is PD01A, a short peptide that induces a specific antibody response to α-syn O. Phase 1 clinical trials with PD01A confirmed tolerance, substantial immune response, and dose-dependent effects [364]. In July 2023, a Phase 2 trial began testing an optimised vaccine formulation named ACI-7104.056 (ClinicalTrials.gov Identifier: NCT06015841).

Examples of α-synuclein antibodies are 9E4 antibody prasinezumab (PRX002) and BIIB054. Prasinezumab is a humanised IgG1 monoclonal antibody directed against aggregated α-synuclein. A Phase 2 clinical trial with prasinezumab concluded that the treatment had no meaningful effect on global or imaging measures of PD progression compared to placebo and was associated with infusion reactions [365]. BIIB054 (also known as cinpanemab) is a human-derived monoclonal α-syn antibody. A multicenter, double-blind, Phase 2 trial (SPARK) concluded that in participants with early PD, over 52 weeks, cinpanemab did not delay or reduce clinical measures of PD progression nor PD-related changes in DaT-SPECT imaging compared to the placebo, control group [366]. A follow-up data analysis of the SPARK study concluded that 52 weeks of treatment with cinpanemab had no effect on the selected biomarkers of patients with early PD and that more suitable biomarkers for disease severity and progression in early PD are necessary [367].

#### 5.2.2. Biguanides

Metformin, a biguanide, is the most prescribed oral antidiabetic drug worldwide for treating T2D. The drug reduces blood glucose by (a) inhibiting MT complex I, which stimulates AMPK and inhibits hepatic glucose production and (b) increasing skeletal muscle glucose uptake by increased GLUT4 incorporation into the cell membrane. Preclinical studies reported that metformin attenuated ROS production [368,369] and the activity of inflammatory cytokines [369,370,371] by attenuating NF-κB activity [372]. Epidemiological studies reported diverse effects of metformin on modifying the risk of PD in T2D patients, ranging from a reduced risk [373] and no change in risk [374] to an increased risk [375,376] of PD in T2D patients.

#### 5.2.3. Dipeptidyl Peptidase-4 Inhibitors

Dipeptidyl peptidase-4 inhibitors (DPP4 inhibitors), also known as gliptins, slow the degradation of their substrate incretin GLP1, thus prolonging GLP1 signalling, which stimulates insulin secretion by pancreatic β cells [377,378] and supports neuroprotective, antiapoptotic, and anti-inflammatory effects in the CNS [379,380,381]. Animal model studies of PD using DPP4 inhibitors (sitagliptin and liraglutide) reported their beneficial effects, including improved motor function, reduced memory deficits, reduced dopaminergic degeneration, reduced oxidative stress and neuroinflammatory markers, attenuated apoptosis, and increased neurogenesis [382,383,384,385,386,387]. Population-based studies, either case–control [388] or longitudinal cohort [140] studies, reported a reduced risk for PD in patients using DPP4 inhibitors. PD patients with diabetes who were treated with DPP4 inhibitors had (a) an increased baseline dopamine transporter availability in the anterior, posterior, and ventral putamen; (b) a slower increase in the required L-DOPA doses; and (c) a reduced rate of L-DOPA-induced dyskinesia [389]. Also, treatment with DPP4 inhibitors attenuated cognitive decline in older T2D patients with or without mild cognitive impairment [390,391,392]. Randomised, controlled clinical trials are necessary to prove the effectiveness of treatment with DPP4 inhibitors in PD patients [245].

#### 5.2.4. Flavonoids

A recent review summarised the potential beneficial effects of flavonoids in plant-based food in preventing and managing synucleinopathies, including PD. Baicalein, luteolin, quercetin, myricitrin, epigallocatechin-3-gallate, and genistein are reported as the most effective flavonoids against synucleinopathies. The PD-preventive effect of flavonoids is attributed to the reduced α-syn production and aggregation, enhanced degradation of α-syn aggregates with autophagy, and modulation of antioxidant enzyme activity, thus protecting dopaminergic neurons from oxidative damage and cell death. The authors concluded that clinical trials to evaluate the long-term safety, optimal dosage, and efficacy of flavonoids in humans are necessary to validate the reported beneficial effects of flavonoids in animal models of PD [393].

#### 5.2.5. Glucagon-like Peptide-1 Receptor Agonists

Glucagon-like Peptide-1 (GLP-1) is an incretin hormone. The ingestion of nutrients stimulates its release from the gut enteroendocrine cells [394]. GLP1 increases insulin secretion by pancreatic β cells and reduces glucagon secretion by pancreatic α cells. In addition, GLP1 has anti-inflammatory, antiapoptotic, and neuroprotective effects [395]. In the CNS, GLP1 regulates thermogenesis, blood pressure control, neural development, growth and regeneration, energy homeostasis, satiety control, water intake, and stress reaction [396].

GLP1 receptor agonists (e.g., exenatide, liraglutide, and semaglutide), with a longer half-life than GLP1, were developed for treating patients with T2D [394,397]. These agonists are candidates for PD treatment since they can cross the BBB and affect brain cells’ metabolism [398,399]. Animal model studies of PD with GLP-1 receptor agonists (exenatide, liraglutide, and semaglutide) reported reduced dopaminergic degeneration, restored dopamine levels, improved motor function [316,400,401,402,403] and attenuated α-synuclein accumulation, reduced oxidative stress and neuroinflammation, improved MT function, stimulated autophagy, and increased glial cell line-derived neurotrophic factor expression and neurogenesis [316,403,404,405]. Clinical trials on PD patients with the GLP1 receptor agonist exenatide reported consistent improvement in motor and cognitive functions. A single-blind trial reported persistent improvement in motor and cognitive functions even 12 months after treatment interruption [406]. These results were confirmed with a follow-up randomised, double-anonymized placebo control study on patients with moderate PD [143,407]. The efficacy of the GLP1 receptor agonists exenatide, liraglutide, lixisenatide, and semaglutide for PD treatment is being evaluated in clinical trials (ClinicalTrials.gov Identifiers: NCT04305002; NCT02953665; NCT03439943; NCT03659682).

#### 5.2.6. Hydroxy-3-methyl-glutaryl-coenzyme A Reductase Inhibitors

3-Hydroxy-3-methylglutaryl-coenzyme A (HMG-CoA) reductase inhibitors, also known as statins, inhibit the conversion of HMG-CoA to mevalonate, the rate-limiting step in cholesterol biosynthesis. They are widely used for treating dyslipidemia. The justification for considering HMG-CoA inhibitors for treating PD is underpinned by preclinical studies reporting the involvement of cholesterol and its metabolites in PD-associated pathology. For example, (a) LBs contain isoforms of isopentenyl diphosphate isomerase (IDI), an enzyme related to cholesterol biosynthesis [408]; (b) cholesterol and its metabolites accelerate α-syn aggregation by proteasomes inhibition and activation of the liver X receptors [356,357]; (c) cholesterol preferentially facilitates α-synO interactions [358,359,360]; and (d) cholesterol metabolites inhibit the expression of tyrosine hydroxylase (TH) and dopamine synthesis and promote oxidative stress, cell death, and inflammation [361]. Epidemiological studies and clinical trials have not consistently reported a positive association between high levels of cholesterol and PD [123,409,410,411,412,413,414].

#### 5.2.7. IL-1β Inhibitors

IL-1β is a microglia-activating pro-inflammatory cytokine [415,416]. In animal models of PD, α-synuclein species increased IL-1β release [417,418] that was associated with PD pathology and cognitive impairment [419,420,421]. A potential IL-1β inhibitor for PD treatment is canakinumab, a human monoclonal IL-1β antibody (also known as ACZ885) that attenuates inflammation and is approved for treating auto-inflammatory diseases [422]. There are no registered clinical trials at ClinicalTrials.gov to evaluate the safety and efficacy of IL-1β inhibitors.

#### 5.2.8. Insulin

With or without concomitant T2D, PD patients have impaired brain insulin signalling [245,423]. Normal brain insulin signalling is vital for brain cells; it enables cell growth and repair, long-term potentiation, regulates apoptosis, attenuates oxidative stress, and reduces dopaminergic cell death. Therefore, insulin resistance accelerates dopaminergic degeneration and the progression of motor and cognitive symptoms [256,424,425]. Studies of PD animal models reported beneficial effects of insulin treatment, including reduced motor impairment and attenuated neuronal loss [426] and improved MT function [427]. A randomised, double-blinded, placebo-controlled trial with 4 weeks of intranasal insulin (INI) application in PD patients improved verbal fluency and Hoehn–Yahr and unified Parkinson’s disease rating scale-part 3 motor scores. Additional, controlled clinical trials are necessary to prove the effectiveness and safety of long-term treatment with INI application in PD patients [423]. Potential drawbacks of long-term INI treatment are (a) the desensitisation of the PI3K pathway, necessitating an increase in INI dosage [423], and (b) an excessive stimulation of NMDA receptors with increased excitotoxicity and cell death [240]. Concerns about the development of adverse effects of chronic INI treatment stimulated research on alternative PD treatment approaches with Glucagon-like Peptide-1 receptor agonists and dipeptidyl peptidase-4 inhibitors.

#### 5.2.9. Lenalidomide

Lenalidomide, a thalidomide analogue, is currently used for treating multiple myeloma and myelodysplastic syndromes [428]. The drug has anti-inflammatory and immune cell-modulating effects, including the inhibition of TNF-α, IL-1, IL-6, and IL-12 expression; the stimulation of T-cell proliferation; and increased IL-2 and IFNγ production [429]. In animal PD models, the drug reduced microgliosis, the expression of pro-inflammatory cytokines, NF-κB activation, and dopaminergic fibre loss in the striatum and improved locomotion [430]. Additional beneficial effects of lenalidomide treatment on animal models of PD include increased BDNF expression in the substantia nigra with improved neuronal survival and normal dopamine levels associated with improved locomotion [431]. To date, there are no registered clinical trials at ClinicalTrials.gov to evaluate the safety and efficacy of the drug.

#### 5.2.10. Nonsteroidal Anti-Inflammatory Drugs (NSAIDs)

Neuroinflammation contributes to the development and progression of NDDs, including PD. Cytokines mediate the neuroinflammation [351]; therefore, NSAIDs could attenuate the development and progression of NDDs. NSAIDs inhibit cyclooxygenase (COX) enzymes; this attenuates the conversion of arachidonic acid to bioactive prostaglandins. COX enzymes exist in two forms: COX1 and COX2. COX1-regulated prostaglandins are involved in various homeostatic functions throughout the body. COX2-regulated prostaglandins mediate pain and inflammation [432]. Reduced levels of COX 2-regulated prostaglandins are associated with reduced inflammatory cytokine levels and attenuated PD neuroinflammation [433]. Preclinical studies on animal models of PD reported that the NSAIDs acetylsalicylic acid, ibuprofen, and indomethacin reduced neuroinflammation, reduced the loss of nigral neurons, restored dopamine levels, and improved locomotor activity [434,435]. Epidemiologic studies of the long-term effects of NSAID do not consistently report a decreased risk of developing PD [436,437,438]. Clinical trials are necessary to determine whether NSAIDs reduce the risk of PD.

#### 5.2.11. Sulfonylureas

Sulfonylureas (e.g., chlorpropamide, glibenclamide, glimepiride, glipizide, tolazamide, and tolbutamide) stimulate pancreatic β-cell insulin secretion and are used for the treatment of T2D [245]. These drugs also attenuate inflammation [439]. Preclinical studies on animal models of PD reported that glibenclamide, by inhibiting microglial ATP-sensitive potassium channels and and sulfonylurea receptor1—Ca^2+^ activated nonselective cation channels, attenuates neuroinflammation and improves spatial learning and memory [440,441,442,443]. A systematic review and meta-analysis did not identify any association between using sulfonylureas and PD risk in T2D patients [374].

#### 5.2.12. Thiazolidinediones

Thiazolidinediones (also known as glitazones) are used for the treatment of T2D. These drugs are PPAR-γ agonists that (a) increase adipokine-elicited insulin sensitivity [245] and (b) attenuate inflammation by inhibition of NF-κB and NLRP3 and activation of MAPK signalling pathways [444,445]. In animal models of PD, the aggregate effects of pioglitazone and rosiglitazone were improved motor cognitive functions, reduced dopaminergic neurodegeneration with improved dopamine levels, improved MT function, and attenuated microglial and astroglial cytokine inflammatory responses [446,447,448,449,450,451,452,453,454]. A randomised, multicenter placebo-controlled study testing pioglitazone reported (a) no effect on disease progression and (b) no change in peripheral biomarkers in patients with PD [455]. A recent meta-analysis of four observational studies concluded that T2D patients treated with thiazolidinediones have a reduced risk for PD [456].

A summary of potential alternative pharmacological treatments for PD is presented in Table 2. The most promising drugs for alternative pharmacological PD treatments are Glucagon-like Peptide-1 (GLP1) agonists or dipeptidyl peptidase-4 enzyme (DPP4) inhibitors (also known as gliptins).

## 6. Conclusions

The view of PD etiopathology has shifted from a brain-centered disease, characterised by motor symptoms, to a multiorgan disease with multisystemic etiopathology and prodromal non-motor symptoms caused by the interactions of several genetic risk factors, environmental factors, and ageing [479].

The clinical diversity of PD signs and symptoms, combined with the incomplete understanding of PD aetiology, necessitate the use of suboptimal combined biomarkers for diagnosis, treatment monitoring, and prognosis and justify the research and development of combined biomarkers better tailored to specific clinical phenotypes [480,481].

Lactate and MGO are possible biomarker candidates for PD-associated dementia. Both were measured in human samples, lactate in CSL [482] and MGO in serum [483], and are associated with brain pathology and reduced cognition.

Epidemiological, preclinical, and clinical research underpins the assumption of overlapping T2D and PD aetiologies due to insulin resistance (IR). T2D-linked systemic IR and the brain IR—expressed together or existing independently—contribute to the initiation and progression of PD-associated α-syn brain pathology.

Systemic IR effects sustaining PD-associated α-syn pathology are hyperglycemia and hyperglycemia-associated brain pathologies (e.g., small blood vessel dysfunction, chronic neuroinflammation, and BBB dysfunction). Brain IR contributes to α-syn brain pathology by promoting α-syn aggregation and deposition and attenuating α-syn species clearance by IDEs, autophagy, and unfolded protein response. PD and T2D pathologies are also associated with chronic inflammation in the brain that interferes with normal MT function and proteasome degradation in brain neurons.

The current lack of treatment interventions that either stop or reverse the progression of PD, combined with the understanding of signalling pathways that underpin the overlapping PD and T2D aetiologies, has encouraged the evaluation of repurposing antidiabetic drugs for PD treatment.

## 7. Study Limitations

This study has the limitations of a narrative review and provides a comprehensive, non-exhaustive overview of the current knowledge of the contribution of diabetes to Parkinson’s disease aetiology.

## Figures and Tables

**Table 1:** Comparison of motor and cognitive signs between patients with PD only and healthy older adults (HOAs) and with patients with PD and T2D. Symbols: + (change in motor or cognitive signs when comparing PD patients to HOAs); ++ (increased change in motor or cognitive sign when comparing PD patients to patients with PD + T2D); = (no additional change in motor or cognitive sign when comparing PD to PD + T2D patients).

Change in Clinical Sign	PD vs. HOA	PD vs. PD + T2D
slower gait	+	++
worse balance	+	++
reduced muscle strength	+	++
reduced motor endurance	+	++
reduced motor–cognitive function	+	=
impaired attention	+	++

**Table 2:** Summary of potential alternative drugs for pharmacological treatment of PD.

Substance	Mechanism of Action	Observed Effects in Preclinical, PD Animal Model and Cell Studies	Results of PD Epidemiological or Clinical Trial Studies
anti α-synuclein protein monomer (α-syn) vaccination and humanised α-synuclein antibodies	Peptides that elicit an antibody response to oligomeric α-synuclein (e.g., PD01A) or humanised antibody (e.g., PRX002).	The effectiveness of active immunization against Parkinson’s disease (PD) (improved locomotor activity, memory and learning, reduced death of pars compacta of substanca nigra (SNpc) nerve cells) was demonstrated in mouse and rat models [363].	Phase 1 clinical trials confirmed tolerance, substantial immune response, and dose-dependent effects [364,457,458]. BIIB054 development terminated after Phase 2 clinical trial [245].
biguanides (i.e., metformin)	(1) Inhibit mitochondrial (MT) complex I, which stimulates AMP-activated protein kinase (AMPK) that inhibits (a) gluconeogenesis and hepatic glucose production and increases skeletal muscle glucose uptake by increased glucose transporter (GLUT)4 incorporation into the cell membrane (b). (2) Inhibit nuclear factor kappa-light-chain-enhancer of activated B cells (NF-κB) and reduce reactive oxidative species (ROS) production.	Overall consistent, positive effects. Improved locomotor activity and motor coordination, reduced degeneration of dopaminergic neurons, reversed dopamine depletion, inhibited α-syn phosphorylation and aggregation, decreased MT dysfunction and oxidative stress, inhibited neuroinflammation, increased the production of neurotrophic factors [459,460,461,462,463,464].	Diverse results of epidemiological studies; either no effect, a decreased risk, or an increased risk of developing PD in type 2 diabetes (T2D) patients [373,374,375,376].
dipeptidyl peptidase-4 (DPP4) inhibitors	Prolong glucagon-like peptide-1 (GLP-1) signalling by inhibiting its degradation.	Improved motor performance; reduced memory deficits, oxidative stress, and dopaminergic degeneration; increased GLP-1 expression in the brain; and reduced neuroinflammation [382,383,384,385,386]. The combination of a DPP4 inhibitor with levodopa was more effective than levodopa alone [384,387].	Epidemiological studies reported a reduced incidence of PD associated with a record of DPP4-inhibitor intake [140,388].
flavonoids	Multiple actions: (a) modulate the activity/expression of the antioxidant enzymes superoxide dismutase, glutathione peroxidase, and endothelial nitric oxide synthase; (b) reduce ROS damage; and (c) promote autophagy.	Reduced excessive α-syn production, oligomerisation, and aggregation; enhanced α-syn autophagy; reduced oxidative damage and apoptosis of dopaminergic neurons [393,465].	None.
Glucagon-like Peptide-1 receptor (GLP-1) agonists	Promote insulin secretion by pancreatic β cells; in the central nervous system (CNS), they have neuroprotective, antiapoptotic, and anti-inflammatory effects.	Reduced dopaminergic degeneration, α-syn accumulation, and neuroinflammation; restored dopamine levels; attenuated motor dysfunction; improved MT function [316,402,403,405].	Consistent improvement in motor and cognitive functions in clinical trials [143,406,466,467].
Hydroxymethylglutaryl-CoA (HMG-CoA) reductase inhibitors	Inhibit the conversion of HMG-CoA to mevalonate, the rate-limiting step in cholesterol synthesis.	Consistent reports of a positive association between high levels of cholesterol and PD cholesterol and cholesterol metabolites accelerated α-syn aggregation, inhibited tyrosine hydroxylase expression, and reduced dopamine synthesis; promoted oxidative stress, cell death, synaptic loss, and neuroinflammation in the CNS [356,357,358,359,360,361,362].	Clinical trials and observational studies report diverse results from a positive association between high cholesterol levels and an increased risk of PD to a reduced risk of PD associated with high cholesterol [410,411,412,413,414,468,469].
interleukin (IL)-1β inhibitors inhibitors	Inhibit α-syn-elicited release of IL-1 β that stimulates additional release of pro-inflammatory cytokines from astrocytes and microglia [415,416,470].	None.	None.
insulin (intranasal insulin (INI) application)	Promotes cell growth and repair, long-term potentiation; reduces apoptosis, oxidative stress, and dopaminergic cell death.	Low-dose, INI application improved motor and MT function and reduced dopaminergic cell death [427,471].	Randomised, double-blinded, placebo-controlled trial improved verbal fluency and Hoehn-Yahr and unified Parkinson’s disease rating scale-part 3 motor scores with 4 weeks of INI [472].
lenalidomide	Inhibits tumour necrosis factor α (TNF-α), IL-1, IL-6, and IL-12 expression; stimulates T-cell proliferation; and increases production of IL-2 and IFNγ.	Reduced microgliosis, attenuated pro-inflammatory cytokine expression and NF-κB activation, attenuated dopaminergic fibre loss, improved locomotor activity, increased SNpc brain-derived neurotrophic factor expression, and improved neuronal survival [430,431].	None.
nonsteroidal anti-inflammatory drugs (NSAIDs)	Inhibit cyclooxygenase enzymes, thus reducing the conversion of arachidonic acid to prostaglandins, which stimulate the release of inflammatory cytokines.	Reduced loss of nigral neurons, restored dopamine levels, improved locomotor activity, reduced neuroinflammation [434,435,473,474].	A limited number of epidemiological studies have concluded that NSAIDs may decrease the risk of PD [436,475].
sulfonylureas	Stimulate pancreatic β-cell insulin secretion by closing sulfonylurea receptor (Sur1)-regulated channels that elicit membrane depolarization, the influx of Ca2+, and insulin release from vesicles. Sur1-regulated channels are also expressed in neurons, astrocytes, microglial cells, oligodendrocytes, and endothelial cells.	Attenuated motor and memory impairment; decreased oxidative stress, the inhibition of NF-κB, and NLR family pyrin domain-containing protein 3 (NLRP3) inflammasome activation; attenuated neuroinflammation; reduced α-syn expression, dopaminergic neuronal damage, and apoptosis [441,442,443,476].	A systematic review and meta-analysis did not identify any association between the use of sulfonylureas and PD risk in T2D patients [374].
thiazolidinediones (TZD)	PPAR-γ agonists that (a) increase adipokine-elicited insulin sensitivity and (b) attenuate inflammation by the inhibition of NF-κB and NLRP3 and the activation of mitogen-activated protein kinase (MAPK) signalling pathways.	Improved motor cognitive functions, reduced dopaminergic neurodegeneration with improved dopamine levels, improved MT function, attenuated microglial and astroglial cytokine inflammatory response [446,448,449,451,452,453,454,477,478].	A meta-analysis of four observational studies concluded that T2D patients treated with TZD have a reduced risk for PD (Hussain et al., 2020 [456]). A randomized, multicenter, placebo-controlled study reported (a) no effect on disease progression and (b) no change in peripheral biomarkers in patients with PD [455].

## Data Availability

Not applicable.

## Abbreviations

6-OHDA	6-hydroxydopamine
ADTIQ	tetrahydroisoquinoline (ADTIQ)
AGE	advanced glycation end product
AKT	protein kinase B
AMPA	α-amino-3-hydroxy-5-methyl-4-isoxazole-propionic acid
AMPK	AMP-activated protein kinase
ATP	adenosine triphosphate
ATP13A2	late endosomal/lysosomal P5-type transport ATPase
BBB	blood–brain barrier
BDNF	brain-derived neurotrophic factor
CNS	central nervous system
CoA	coenzyme A
COMT	catechol-O-methyl transferase
CRP	C-reactive protein
CSF	cerebrospinal fluid
COX	cyclooxygenase
DAT	dopamine transporter
DJ1	protein deglycase DJ1
DLB	dementia with Lewy Bodies
DNA	deoxyribonucleic acid
DPP4s	dipeptidyl peptidase-4 enzyme inhibitors, also known as gliptins
ER	endoplasmic reticulum
FAD	flavin adenine dinucleotide
FADH2	dihydroflavine-adenine dinucleotide
FAT10	ubiquitin-like modifier HLA-F adjacent transcript 10, or ubiquitin D
FOXO1	forkhead box protein O1
G3P	glycerol-3-phosphate
G6PD	glucose-6-phosphate dehydrogenase
GA	Golgi apparatus
GABA	gamma-aminobutyric acid
GAPDH	glyceraldehyde-3-phosphate dehydrogenase
GI	gastrointestinal
GLP1	Glucagon-like Peptide-1
GLUT	glucose transporter
GSH	reduced glutathione
GSK3β	glycogen synthase kinase 3β
GSSG	glutathione disulfide, oxidised glutathione
GTP	guanosine-5′-triphosphate
HDAC	histone deacetylase
HIF1	hypoxia-inducible factor 1
HMG-CoA	3-Hydroxy-3-methylglutaryl-coenzyme A
IDE	insulin-degrading enzyme
IFNγ	interferon gamma
IL	interleukin
INI	intranasal insulin
IR	insulin resistance
IRS-1	insulin receptor substrate 1
KGDHC	alpha-ketoglutarate dehydrogenase complex
L-DOPA	levodopa
LBs	Lewy Bodies
MAPK	mitogen-activated protein kinase
MGO	methylglyoxal
MIDN	Midnolin
MOA-B	monoamine oxidase B
MPTP	1-methyl-4-phenyl-1,2,3,6-tetrahydropyridine
MSA	multiple system atrophy
MT	mitochondria, mitochondrial
mTORC1	mTOR Complex 1
MTPTP	mitochondrial permeability transition pore
NAD	nicotinamide adenine dinucleotide
NADH	reduced nicotinamide adenine dinucleotide
NADPH	reduced nicotinamide adenine dinucleotide phosphate
NDD	neurodegenerative brain disorder
NF-κB	nuclear factor kappa-light-chain-enhancer of activated B cells
NLRP3	NLR family pyrin domain-containing protein 3
NMDA	N-methyl-D-aspartate
Nrf 2	nuclear factor erythroid 2-related factor
NSAIDs	nonsteroidal anti-inflammatory drugs
PARK2	encodes cytosolic ubiquitin-E3- ligase, the Parkin protein FAT10
Parkin	465-amino acid residue E3 ubiquitin ligase
PD	Parkinson’s disease
PGC1α	peroxisome proliferator-activated receptor-γ coactivator 1-α
PGK1	phosphoglycerate kinase 1
PI3K	phosphatidylinositol 3-kinase
PINK1	protein kinase with a mitochondrial targeting domain
PPP	pentose phosphate pathway
RAGEs	receptors for advanced glycation end products
RBCs	red blood cells
RC	respiratory complex of the electron transport chain
RNA	ribonucleic acid
ROS	reactive oxidative species
SNARE	protein containing a characteristic 60–70 residue domain, the SNARE motif
SNCA	Synuclein Alpha gene
SNpc	pars compacta of substanca nigra
T2D	Type 2 diabetes
TCA	tricarboxylic acid
TNFα	tumour necrosis factor α
TXNIP	thioredoxin-binding protein
UPR	unfolded protein response
VaP	vascular Parkinsonism
VMAT2	vesicular monoamine transporter 2
α-syn	α-synuclein protein monomer
α-synO	α-synuclein oligomer

## References

[1] Yan Y., Shimoga D., Sharma A. (2023). Parkinson’s Disease and Diabetes Mellitus: Synergistic Effects on Pathophysiology and GI Motility. Curr. Gastroenterol. Rep..

[2] Cullinane P.W., de Pablo Fernandez E., Konig A., Outeiro T.F., Jaunmuktane Z., Warner T.T. (2023). Type 2 Diabetes and Parkinson’s Disease: A Focused Review of Current Concepts. Mov. Disord..

[3] Poewe W., Seppi K., Tanner C.M., Halliday G.M., Brundin P., Volkmann J., Schrag A.E., Lang A.E. (2017). Parkinson disease. Nat. Rev. Dis. Primers.

[4] Nussbaum R.L., Ellis C.E. (2003). Alzheimer’s disease and Parkinson’s disease. N. Engl. J. Med..

[5] Bernal-Conde L.D., Ramos-Acevedo R., Reyes-Hernandez M.A., Balbuena-Olvera A.J., Morales-Moreno I.D., Arguero-Sanchez R., Schule B., Guerra-Crespo M. (2019). Alpha-Synuclein Physiology and Pathology: A Perspective on Cellular Structures and Organelles. Front. Neurosci..

[6] Stevenson T.J., Murray H.C., Turner C., Faull R.L.M., Dieriks B.V., Curtis M.A. (2020). alpha-synuclein inclusions are abundant in non-neuronal cells in the anterior olfactory nucleus of the Parkinson’s disease olfactory bulb. Sci. Rep..

[7] Ni A., Ernst C. (2022). Evidence That Substantia Nigra Pars Compacta Dopaminergic Neurons Are Selectively Vulnerable to Oxidative Stress Because They Are Highly Metabolically Active. Front. Cell. Neurosci..

[8] Brichta L., Greengard P. (2014). Molecular determinants of selective dopaminergic vulnerability in Parkinson’s disease: An update. Front. Neuroanat..

[9] Pacelli C., Giguere N., Bourque M.J., Levesque M., Slack R.S., Trudeau L.E. (2015). Elevated Mitochondrial Bioenergetics and Axonal Arborization Size Are Key Contributors to the Vulnerability of Dopamine Neurons. Curr. Biol..

[10] Jaumotte J.D., Wyrostek S.L., Zigmond M.J. (2016). Protection of cultured dopamine neurons from MPP(+) requires a combination of neurotrophic factors. Eur. J. Neurosci..

[11] Bolam J.P., Pissadaki E.K. (2012). Living on the edge with too many mouths to feed: Why dopamine neurons die. Mov. Disord..

[12] Surmeier D.J., Guzman J.N., Sanchez J., Schumacker P.T. (2012). Physiological phenotype and vulnerability in Parkinson’s disease. Cold Spring Harb. Perspect. Med..

[13] Puopolo M., Raviola E., Bean B.P. (2007). Roles of subthreshold calcium current and sodium current in spontaneous firing of mouse midbrain dopamine neurons. J. Neurosci..

[14] Goldberg J.A., Guzman J.N., Estep C.M., Ilijic E., Kondapalli J., Sanchez-Padilla J., Surmeier D.J. (2012). Calcium entry induces mitochondrial oxidant stress in vagal neurons at risk in Parkinson’s disease. Nat. Neurosci..

[15] Bell S., McCarty V., Peng H., Jefri M., Hettige N., Antonyan L., Crapper L., O’Leary L.A., Zhang X., Zhang Y. (2021). Lesch-Nyhan disease causes impaired energy metabolism and reduced developmental potential in midbrain dopaminergic cells. Stem Cell Rep..

[16] Pristera A., Lin W., Kaufmann A.K., Brimblecombe K.R., Threlfell S., Dodson P.D., Magill P.J., Fernandes C., Cragg S.J., Ang S.L. (2015). Transcription factors FOXA1 and FOXA2 maintain dopaminergic neuronal properties and control feeding behavior in adult mice. Proc. Natl. Acad. Sci. USA.

[17] Abdi I.Y., Ghanem S.S., El-Agnaf O.M. (2022). Immune-related biomarkers for Parkinson’s disease. Neurobiol. Dis..

[18] Roverato N.D., Sailer C., Catone N., Aichem A., Stengel F., Groettrup M. (2021). Parkin is an E3 ligase for the ubiquitin-like modifier FAT10, which inhibits Parkin activation and mitophagy. Cell Rep..

[19] Sircar E., Rai S.R., Wilson M.A., Schlossmacher M.G., Sengupta R. (2021). Neurodegeneration: Impact of S-nitrosylated Parkin, DJ-1 and PINK1 on the pathogenesis of Parkinson’s disease. Arch. Biochem. Biophys..

[20] Gundogdu M., Tadayon R., Salzano G., Shaw G.S., Walden H. (2021). A mechanistic review of Parkin activation. Biochim. Biophys. Acta Gen. Subj..

[21] Sagehashi N., Obara Y., Maruyama O., Nakagawa T., Hosoi T., Ishii K. (2022). Insulin Enhances Gene Expression of Midnolin, a Novel Genetic Risk Factor for Parkinson’s Disease, via Extracellular Signal-Regulated Kinase, Phosphoinositide 3-Kinase and Multiple Transcription Factors in SH-SY5Y Cells. J. Pharmacol. Exp. Ther..

[22] Collier T.J., Kanaan N.M., Kordower J.H. (2017). Aging and Parkinson’s disease: Different sides of the same coin?. Mov. Disord..

[23] Bennett D.A., Beckett L.A., Murray A.M., Shannon K.M., Goetz C.G., Pilgrim D.M., Evans D.A. (1996). Prevalence of parkinsonian signs and associated mortality in a community population of older people. N. Engl. J. Med..

[24] Tysnes O.B., Storstein A. (2017). Epidemiology of Parkinson’s disease. J. Neural. Transm..

[25] Mehta N., Luthra N.S., Corcos D.M., Fantuzzi G. (2023). C-reactive protein as the biomarker of choice to monitor the effects of exercise on inflammation in Parkinson’s disease. Front. Immunol..

[26] Santiago J.A., Potashkin J.A. (2014). System-based approaches to decode the molecular links in Parkinson’s disease and diabetes. Neurobiol. Dis..

[27] Khang R., Park C., Shin J.H. (2015). Dysregulation of parkin in the substantia nigra of db/db and high-fat diet mice. Neuroscience.

[28] Dai C., Tan C., Zhao L., Liang Y., Liu G., Liu H., Zhong Y., Liu Z., Mo L., Liu X. (2023). Glucose metabolism impairment in Parkinson’s disease. Brain Res. Bull..

[29] Sheng L., Stewart T., Yang D., Thorland E., Soltys D., Aro P., Khrisat T., Xie Z., Li N., Liu Z. (2020). Erythrocytic alpha-synuclein contained in microvesicles regulates astrocytic glutamate homeostasis: A new perspective on Parkinson’s disease pathogenesis. Acta Neuropathol. Commun..

[30] Matsumoto J., Stewart T., Sheng L., Li N., Bullock K., Song N., Shi M., Banks W.A., Zhang J. (2017). Transmission of alpha-synuclein-containing erythrocyte-derived extracellular vesicles across the blood-brain barrier via adsorptive mediated transcytosis: Another mechanism for initiation and progression of Parkinson’s disease?. Acta Neuropathol. Commun..

[31] Liu Z., Chan R.B., Cai Z., Liu X., Wu Y., Yu Z., Feng T., Yang Y., Zhang J. (2022). alpha-Synuclein-containing erythrocytic extracellular vesicles: Essential contributors to hyperactivation of monocytes in Parkinson’s disease. J. Neuroinflamm..

[32] Calabresi P., Di Lazzaro G., Marino G., Campanelli F., Ghiglieri V. (2023). Advances in understanding the function of alpha-synuclein: Implications for Parkinson’s disease. Brain.

[33] El-Agnaf O.M.A., Salem S.A., Paleologou K.E., Cooper L.J., Fullwood N.J., Gibson M.J., Curran M.D., Court J.A., Mann D.M.A., Ikeda S.-I. (2003). α-Synuclein implicated in Parkinson’s disease is present in extracellular biological fluids, including human plasma. FASEB J..

[34] Adler C.H., Beach T.G. (2016). Neuropathological basis of nonmotor manifestations of Parkinson’s disease. Mov. Disord..

[35] Beach T.G., Adler C.H., Lue L., Sue L.I., Bachalakuri J., Henry-Watson J., Sasse J., Boyer S., Shirohi S., Brooks R. (2009). Unified staging system for Lewy body disorders: Correlation with nigrostriatal degeneration, cognitive impairment and motor dysfunction. Acta Neuropathol..

[36] Braak H., Rub U., Gai W.P., Del Tredici K. (2003). Idiopathic Parkinson’s disease: Possible routes by which vulnerable neuronal types may be subject to neuroinvasion by an unknown pathogen. J. Neural. Transm..

[37] Luk K.C., Song C., O’Brien P., Stieber A., Branch J.R., Brunden K.R., Trojanowski J.Q., Lee V.M. (2009). Exogenous alpha-synuclein fibrils seed the formation of Lewy body-like intracellular inclusions in cultured cells. Proc. Natl. Acad. Sci. USA.

[38] Volpicelli-Daley L.A., Luk K.C., Patel T.P., Tanik S.A., Riddle D.M., Stieber A., Meaney D.F., Trojanowski J.Q., Lee V.M. (2011). Exogenous alpha-synuclein fibrils induce Lewy body pathology leading to synaptic dysfunction and neuron death. Neuron.

[39] Tsigelny I.F., Sharikov Y., Wrasidlo W., Gonzalez T., Desplats P.A., Crews L., Spencer B., Masliah E. (2012). Role of alpha-synuclein penetration into the membrane in the mechanisms of oligomer pore formation. FEBS J..

[40] Froula J.M., Castellana-Cruz M., Anabtawi N.M., Camino J.D., Chen S.W., Thrasher D.R., Freire J., Yazdi A.A., Fleming S., Dobson C.M. (2019). Defining alpha-synuclein species responsible for Parkinson’s disease phenotypes in mice. J. Biol. Chem..

[41] Quist A., Doudevski I., Lin H., Azimova R., Ng D., Frangione B., Kagan B., Ghiso J., Lal R. (2005). Amyloid ion channels: A common structural link for protein-misfolding disease. Proc. Natl. Acad. Sci. USA.

[42] Angelova P.R., Ludtmann M.H., Horrocks M.H., Negoda A., Cremades N., Klenerman D., Dobson C.M., Wood N.W., Pavlov E.V., Gandhi S. (2016). Ca^2+^ is a key factor in alpha-synuclein-induced neurotoxicity. J. Cell Sci..

[43] Fusco G., Chen S.W., Williamson P.T.F., Cascella R., Perni M., Jarvis J.A., Cecchi C., Vendruscolo M., Chiti F., Cremades N. (2017). Structural basis of membrane disruption and cellular toxicity by alpha-synuclein oligomers. Science.

[44] Emmanouilidou E., Melachroinou K., Roumeliotis T., Garbis S.D., Ntzouni M., Margaritis L.H., Stefanis L., Vekrellis K. (2010). Cell-produced alpha-synuclein is secreted in a calcium-dependent manner by exosomes and impacts neuronal survival. J. Neurosci..

[45] Chinta S.J., Mallajosyula J.K., Rane A., Andersen J.K. (2010). Mitochondrial alpha-synuclein accumulation impairs complex I function in dopaminergic neurons and results in increased mitophagy in vivo. Neurosci. Lett..

[46] Martinez J.H., Fuentes F., Vanasco V., Alvarez S., Alaimo A., Cassina A., Coluccio Leskow F., Velazquez F. (2018). Alpha-synuclein mitochondrial interaction leads to irreversible translocation and complex I impairment. Arch. Biochem. Biophys..

[47] Lee H.J., Khoshaghideh F., Lee S., Lee S.J. (2006). Impairment of microtubule-dependent trafficking by overexpression of alpha-synuclein. Eur. J. Neurosci..

[48] Fujita Y., Ohama E., Takatama M., Al-Sarraj S., Okamoto K. (2006). Fragmentation of Golgi apparatus of nigral neurons with alpha-synuclein-positive inclusions in patients with Parkinson’s disease. Acta Neuropathol..

[49] Fan J., Hu Z., Zeng L., Lu W., Tang X., Zhang J., Li T. (2008). Golgi apparatus and neurodegenerative diseases. Int. J. Dev. Neurosci..

[50] Sugeno N., Takeda A., Hasegawa T., Kobayashi M., Kikuchi A., Mori F., Wakabayashi K., Itoyama Y. (2008). Serine 129 phosphorylation of alpha-synuclein induces unfolded protein response-mediated cell death. J. Biol. Chem..

[51] Heman-Ackah S.M., Manzano R., Hoozemans J.J.M., Scheper W., Flynn R., Haerty W., Cowley S.A., Bassett A.R., Wood M.J.A. (2017). Alpha-synuclein induces the unfolded protein response in Parkinson’s disease SNCA triplication iPSC-derived neurons. Hum. Mol. Genet..

[52] Kamp F., Exner N., Lutz A.K., Wender N., Hegermann J., Brunner B., Nuscher B., Bartels T., Giese A., Beyer K. (2010). Inhibition of mitochondrial fusion by alpha-synuclein is rescued by PINK1, Parkin and DJ-1. EMBO J..

[53] Rostovtseva T.K., Gurnev P.A., Protchenko O., Hoogerheide D.P., Yap T.L., Philpott C.C., Lee J.C., Bezrukov S.M. (2015). alpha-Synuclein Shows High Affinity Interaction with Voltage-dependent Anion Channel, Suggesting Mechanisms of Mitochondrial Regulation and Toxicity in Parkinson Disease. J. Biol. Chem..

[54] Desplats P., Spencer B., Coffee E., Patel P., Michael S., Patrick C., Adame A., Rockenstein E., Masliah E. (2011). Alpha-synuclein sequesters Dnmt1 from the nucleus: A novel mechanism for epigenetic alterations in Lewy body diseases. J. Biol. Chem..

[55] Kontopoulos E., Parvin J.D., Feany M.B. (2006). Alpha-synuclein acts in the nucleus to inhibit histone acetylation and promote neurotoxicity. Hum. Mol. Genet..

[56] Liu M., Qin L., Wang L., Tan J., Zhang H., Tang J., Shen X., Tan L., Wang C. (2018). alpha-synuclein induces apoptosis of astrocytes by causing dysfunction of the endoplasmic reticulum-Golgi compartment. Mol. Med. Rep..

[57] Paiva I., Jain G., Lazaro D.F., Jercic K.G., Hentrich T., Kerimoglu C., Pinho R., Szego E.M., Burkhardt S., Capece V. (2018). Alpha-synuclein deregulates the expression of COL4A2 and impairs ER-Golgi function. Neurobiol. Dis..

[58] Lee Y.J., Wang S., Slone S.R., Yacoubian T.A., Witt S.N. (2011). Defects in very long chain fatty acid synthesis enhance alpha-synuclein toxicity in a yeast model of Parkinson’s disease. PLoS ONE.

[59] Betzer C., Lassen L.B., Olsen A., Kofoed R.H., Reimer L., Gregersen E., Zheng J., Cali T., Gai W.P., Chen T. (2018). Alpha-synuclein aggregates activate calcium pump SERCA leading to calcium dysregulation. EMBO Rep..

[60] Wakabayashi K., Tanji K., Mori F., Takahashi H. (2007). The Lewy body in Parkinson’s disease: Molecules implicated in the formation and degradation of alpha-synuclein aggregates. Neuropathology.

[61] Ono K., Takahashi R., Ikeda T., Yamada M. (2012). Cross-seeding effects of amyloid beta-protein and alpha-synuclein. J. Neurochem..

[62] Yacoubian T.A., Standaert D.G. (2014). Reaping what you sow: Cross-seeding between aggregation-prone proteins in neurodegeneration. Mov. Disord..

[63] Sharma S.K., Chorell E., Wittung-Stafshede P. (2015). Insulin-degrading enzyme is activated by the C-terminus of alpha-synuclein. Biochem. Biophys. Res. Commun..

[64] Zhao L., Teter B., Morihara T., Lim G.P., Ambegaokar S.S., Ubeda O.J., Frautschy S.A., Cole G.M. (2004). Insulin-degrading enzyme as a downstream target of insulin receptor signaling cascade: Implications for Alzheimer’s disease intervention. J. Neurosci..

[65] Sharma S.K., Chorell E., Steneberg P., Vernersson-Lindahl E., Edlund H., Wittung-Stafshede P. (2015). Insulin-degrading enzyme prevents alpha-synuclein fibril formation in a nonproteolytical manner. Sci. Rep..

[66] Mergenthaler P., Lindauer U., Dienel G.A., Meisel A. (2013). Sugar for the brain: The role of glucose in physiological and pathological brain function. Trends Neurosci..

[67] Dienel G.A. (2019). Brain Glucose Metabolism: Integration of Energetics with Function. Physiol. Rev..

[68] Yu Y., Herman P., Rothman D.L., Agarwal D., Hyder F. (2018). Evaluating the gray and white matter energy budgets of human brain function. J. Cereb. Blood Flow. Metab..

[69] Ashrafi G., Wu Z., Farrell R.J., Ryan T.A. (2017). GLUT4 Mobilization Supports Energetic Demands of Active Synapses. Neuron.

[70] Pearson-Leary J., McNay E.C. (2016). Novel Roles for the Insulin-Regulated Glucose Transporter-4 in Hippocampally Dependent Memory. J. Neurosci..

[71] Simpson I.A., Carruthers A., Vannucci S.J. (2007). Supply and demand in cerebral energy metabolism: The role of nutrient transporters. J. Cereb. Blood Flow. Metab..

[72] Mielke R., Kessler J., Szelies B., Herholz K., Wienhard K., Heiss W.D. (1998). Normal and pathological aging--findings of positron-emission-tomography. J. Neural. Transm..

[73] Belanger M., Allaman I., Magistretti P.J. (2011). Brain energy metabolism: Focus on astrocyte-neuron metabolic cooperation. Cell Metab..

[74] Trist B.G., Hare D.J., Double K.L. (2019). Oxidative stress in the aging substantia nigra and the etiology of Parkinson’s disease. Aging Cell.

[75] Marques A., Dutheil F., Durand E., Rieu I., Mulliez A., Fantini M.L., Boirie Y., Durif F. (2018). Glucose dysregulation in Parkinson’s disease: Too much glucose or not enough insulin?. Parkinsonism Relat. Disord..

[76] Liu W., Tang J. (2021). Association between diabetes mellitus and risk of Parkinson’s disease: A prisma-compliant meta-analysis. Brain Behav..

[77] Sanchez-Gomez A., Diaz Y., Duarte-Salles T., Compta Y., Marti M.J. (2021). Prediabetes, type 2 diabetes mellitus and risk of Parkinson’s disease: A population-based cohort study. Parkinsonism Relat. Disord..

[78] Rhee S.Y., Lee W.Y. (2021). Association Between Glycemic Status and the Risk of Parkinson Disease: A Nationwide Population-Based Study. Diabetes Care 2020;43:2169-2175. Diabetes Care.

[79] Klimek P., Kautzky-Willer A., Chmiel A., Schiller-Fruhwirth I., Thurner S. (2015). Quantification of diabetes comorbidity risks across life using nation-wide big claims data. PLoS Comput. Biol..

[80] Chung H.S., Lee J.S., Kim J.A., Roh E., Lee Y.B., Hong S.H., Yu J.H., Kim N.H., Yoo H.J., Seo J.A. (2021). Fasting plasma glucose variability in midlife and risk of Parkinson’s disease: A nationwide population-based study. Diabetes Metab..

[81] De Pablo-Fernandez E., Goldacre R., Pakpoor J., Noyce A.J., Warner T.T. (2018). Association between diabetes and subsequent Parkinson disease: A record-linkage cohort study. Neurology.

[82] Chohan H., Senkevich K., Patel R.K., Bestwick J.P., Jacobs B.M., Bandres Ciga S., Gan-Or Z., Noyce A.J. (2021). Type 2 Diabetes as a Determinant of Parkinson’s Disease Risk and Progression. Mov. Disord..

[83] Jeong S.M., Han K., Kim D., Rhee S.Y., Jang W., Shin D.W. (2020). Body mass index, diabetes, and the risk of Parkinson’s disease. Mov. Disord..

[84] Meyer P.T., Frings L., Hellwig S. (2014). Update on SPECT and PET in parkinsonism—Part 2: Biomarker imaging of cognitive impairment in Lewy-body diseases. Curr. Opin. Neurol..

[85] Dunn L., Allen G.F., Mamais A., Ling H., Li A., Duberley K.E., Hargreaves I.P., Pope S., Holton J.L., Lees A. (2014). Dysregulation of glucose metabolism is an early event in sporadic Parkinson’s disease. Neurobiol. Aging.

[86] Eggers C., Hilker R., Burghaus L., Schumacher B., Heiss W.D. (2009). High resolution positron emission tomography demonstrates basal ganglia dysfunction in early Parkinson’s disease. J. Neurol. Sci..

[87] Borghammer P., Chakravarty M., Jonsdottir K.Y., Sato N., Matsuda H., Ito K., Arahata Y., Kato T., Gjedde A. (2010). Cortical hypometabolism and hypoperfusion in Parkinson’s disease is extensive: Probably even at early disease stages. Brain Struct. Funct..

[88] Szturm T., Beheshti I., Mahana B., Hobson D.E., Goertzen A., Ko J.H. (2021). Imaging Cerebral Glucose Metabolism during Dual-Task Walking in Patients with Parkinson’s disease. J. Neuroimaging.

[89] Berding G., Odin P., Brooks D.J., Nikkhah G., Matthies C., Peschel T., Shing M., Kolbe H., van Den Hoff J., Fricke H. (2001). Resting regional cerebral glucose metabolism in advanced Parkinson’s disease studied in the off and on conditions with [(18)F]FDG-PET. Mov. Disord..

[90] Li D., Zuo C., Guan Y., Zhao Y., Shen J., Zan S., Sun B. (2006). FDG-PET study of the bilateral subthalamic nucleus stimulation effects on the regional cerebral metabolism in advanced Parkinson disease. Acta Neurochir. Suppl..

[91] Firbank M.J., Yarnall A.J., Lawson R.A., Duncan G.W., Khoo T.K., Petrides G.S., O’Brien J.T., Barker R.A., Maxwell R.J., Brooks D.J. (2017). Cerebral glucose metabolism and cognition in newly diagnosed Parkinson’s disease: ICICLE-PD study. J. Neurol. Neurosurg. Psychiatry.

[92] Vander Borght T., Minoshima S., Giordani B., Foster N.L., Frey K.A., Berent S., Albin R.L., Koeppe R.A., Kuhl D.E. (1997). Cerebral metabolic differences in Parkinson’s and Alzheimer’s diseases matched for dementia severity. J. Nucl. Med..

[93] Peppard R.F., Martin W.R., Carr G.D., Grochowski E., Schulzer M., Guttman M., McGeer P.L., Phillips A.G., Tsui J.K., Calne D.B. (1992). Cerebral glucose metabolism in Parkinson’s disease with and without dementia. Arch. Neurol..

[94] Sakaue S., Kasai T., Mizuta I., Suematsu M., Osone S., Azuma Y., Imamura T., Tokuda T., Kanno H., El-Agnaf O.M.A. (2017). Early-onset parkinsonism in a pedigree with phosphoglycerate kinase deficiency and a heterozygous carrier: Do PGK-1 mutations contribute to vulnerability to parkinsonism?. npj Parkinsons Dis..

[95] Sotiriou E., Greene P., Krishna S., Hirano M., DiMauro S. (2010). Myopathy and parkinsonism in phosphoglycerate kinase deficiency. Muscle Nerve.

[96] Cai R., Zhang Y., Simmering J.E., Schultz J.L., Li Y., Fernandez-Carasa I., Consiglio A., Raya A., Polgreen P.M., Narayanan N.S. (2019). Enhancing glycolysis attenuates Parkinson’s disease progression in models and clinical databases. J. Clin. Investig..

[97] Chen X., Zhao C., Li X., Wang T., Li Y., Cao C., Ding Y., Dong M., Finci L., Wang J.H. (2015). Terazosin activates Pgk1 and Hsp90 to promote stress resistance. Nat. Chem. Biol..

[98] Simmering J.E., Welsh M.J., Liu L., Narayanan N.S., Pottegard A. (2021). Association of Glycolysis-Enhancing alpha-1 Blockers With Risk of Developing Parkinson Disease. JAMA Neurol..

[99] Kim J.W., Tchernyshyov I., Semenza G.L., Dang C.V. (2006). HIF-1-mediated expression of pyruvate dehydrogenase kinase: A metabolic switch required for cellular adaptation to hypoxia. Cell Metab..

[100] Requejo-Aguilar R., Lopez-Fabuel I., Fernandez E., Martins L.M., Almeida A., Bolanos J.P. (2014). PINK1 deficiency sustains cell proliferation by reprogramming glucose metabolism through HIF1. Nat. Commun..

[101] Barinova K., Khomyakova E., Semenyuk P., Schmalhausen E., Muronetz V. (2018). Binding of alpha-synuclein to partially oxidized glyceraldehyde-3-phosphate dehydrogenase induces subsequent inactivation of the enzyme. Arch. Biochem. Biophys..

[102] Melnikova A., Pozdyshev D., Barinova K., Kudryavtseva S., Muronetz V.I. (2020). alpha-Synuclein Overexpression in SH-SY5Y Human Neuroblastoma Cells Leads to the Accumulation of Thioflavin S-positive Aggregates and Impairment of Glycolysis. Biochemistry.

[103] Semenyuk P., Barinova K., Muronetz V. (2019). Glycation of alpha-synuclein amplifies the binding with glyceraldehyde-3-phosphate dehydrogenase. Int. J. Biol. Macromol..

[104] Mizuno Y., Matuda S., Yoshino H., Mori H., Hattori N., Ikebe S. (1994). An immunohistochemical study on alpha-ketoglutarate dehydrogenase complex in Parkinson’s disease. Ann. Neurol..

[105] Gerlach M., Riederer P., Przuntek H., Youdim M.B. (1991). MPTP mechanisms of neurotoxicity and their implications for Parkinson’s disease. Eur. J. Pharmacol..

[106] Keeney P.M., Xie J., Capaldi R.A., Bennett J.P. (2006). Parkinson’s disease brain mitochondrial complex I has oxidatively damaged subunits and is functionally impaired and misassembled. J. Neurosci..

[107] Mann V.M., Cooper J.M., Daniel S.E., Srai K., Jenner P., Marsden C.D., Schapira A.H. (1994). Complex I, iron, and ferritin in Parkinson’s disease substantia nigra. Ann. Neurol..

[108] Muftuoglu M., Elibol B., Dalmizrak O., Ercan A., Kulaksiz G., Ogus H., Dalkara T., Ozer N. (2004). Mitochondrial complex I and IV activities in leukocytes from patients with parkin mutations. Mov. Disord..

[109] Devi L., Raghavendran V., Prabhu B.M., Avadhani N.G., Anandatheerthavarada H.K. (2008). Mitochondrial import and accumulation of alpha-synuclein impair complex I in human dopaminergic neuronal cultures and Parkinson disease brain. J. Biol. Chem..

[110] Palacino J.J., Sagi D., Goldberg M.S., Krauss S., Motz C., Wacker M., Klose J., Shen J. (2004). Mitochondrial dysfunction and oxidative damage in parkin-deficient mice. J. Biol. Chem..

[111] Heck R.W., Tanhauser S.M., Manda R., Tu C., Laipis P.J., Silverman D.N. (1994). Catalytic properties of mouse carbonic anhydrase V. J. Biol. Chem..

[112] Poon H.F., Frasier M., Shreve N., Calabrese V., Wolozin B., Butterfield D.A. (2005). Mitochondrial associated metabolic proteins are selectively oxidized in A30P alpha-synuclein transgenic mice--a model of familial Parkinson’s disease. Neurobiol. Dis..

[113] Cui T., Fan C., Gu L., Gao H., Liu Q., Zhang T., Qi Z., Zhao C., Zhao H., Cai Q. (2011). Silencing of PINK1 induces mitophagy via mitochondrial permeability transition in dopaminergic MN9D cells. Brain Res..

[114] Dagda R.K., Cherra S.J., Kulich S.M., Tandon A., Park D., Chu C.T. (2009). Loss of PINK1 function promotes mitophagy through effects on oxidative stress and mitochondrial fission. J. Biol. Chem..

[115] McCoy M.K., Cookson M.R. (2012). Mitochondrial quality control and dynamics in Parkinson’s disease. Antioxid. Redox Signal..

[116] Gegg M.E., Cooper J.M., Schapira A.H., Taanman J.W. (2009). Silencing of PINK1 expression affects mitochondrial DNA and oxidative phosphorylation in dopaminergic cells. PLoS ONE.

[117] Morais V.A., Verstreken P., Roethig A., Smet J., Snellinx A., Vanbrabant M., Haddad D., Frezza C., Mandemakers W., Vogt-Weisenhorn D. (2009). Parkinson’s disease mutations in PINK1 result in decreased Complex I activity and deficient synaptic function. EMBO Mol. Med..

[118] Ge T., Yang J., Zhou S., Wang Y., Li Y., Tong X. (2020). The Role of the Pentose Phosphate Pathway in Diabetes and Cancer. Front. Endocrinol..

[119] Alecu I., Bennett S.A.L. (2019). Dysregulated Lipid Metabolism and Its Role in alpha-Synucleinopathy in Parkinson’s Disease. Front. Neurosci..

[120] Haythorne E., Rohm M., van de Bunt M., Brereton M.F., Tarasov A.I., Blacker T.S., Sachse G., Silva Dos Santos M., Terron Exposito R., Davis S. (2019). Diabetes causes marked inhibition of mitochondrial metabolism in pancreatic beta-cells. Nat. Commun..

[121] Tu D., Gao Y., Yang R., Guan T., Hong J.S., Gao H.M. (2019). The pentose phosphate pathway regulates chronic neuroinflammation and dopaminergic neurodegeneration. J. Neuroinflamm..

[122] Fecchio C., Palazzi L., de Laureto P.P. (2018). alpha-Synuclein and Polyunsaturated Fatty Acids: Molecular Basis of the Interaction and Implication in Neurodegeneration. Molecules.

[123] Bosco D.A., Fowler D.M., Zhang Q., Nieva J., Powers E.T., Wentworth P., Lerner R.A., Kelly J.W. (2006). Elevated levels of oxidized cholesterol metabolites in Lewy body disease brains accelerate alpha-synuclein fibrilization. Nat. Chem. Biol..

[124] de Pablo-Fernandez E., Courtney R., Rockliffe A., Gentleman S., Holton J.L., Warner T.T. (2021). Faster disease progression in Parkinson’s disease with type 2 diabetes is not associated with increased alpha-synuclein, tau, amyloid-beta or vascular pathology. Neuropathol. Appl. Neurobiol..

[125] Wang T., Yuan F., Chen Z., Zhu S., Chang Z., Yang W., Deng B., Que R., Cao P., Chao Y. (2020). Vascular, inflammatory and metabolic risk factors in relation to dementia in Parkinson’s disease patients with type 2 diabetes mellitus. Aging.

[126] Pagano G., Polychronis S., Wilson H., Giordano B., Ferrara N., Niccolini F., Politis M. (2018). Diabetes mellitus and Parkinson disease. Neurology.

[127] Laiteerapong N., Huang E.S., Cowie C.C., Casagrande S.S., Menke A., Cissell M.A., Eberhardt M.S., Meigs J.B., Gregg E.W., Knowler W.C., Barrett-Connor E., Becker D.J. (2018). Diabetes in Older Adults. Diabetes in America.

[128] (2021). Introduction: Standards of Medical Care in Diabetes—2021. Diabetes Care.

[129] Hirtz D., Thurman D.J., Gwinn-Hardy K., Mohamed M., Chaudhuri A.R., Zalutsky R. (2007). How common are the “common” neurologic disorders?. Neurology.

[130] De Rijk M.C., Launer L.J., Berger K., Breteler M.M., Dartigues J.F., Baldereschi M., Fratiglioni L., Lobo A., Martinez-Lage J., Trenkwalder C. (2000). Prevalence of Parkinson’s disease in Europe: A collaborative study of population-based cohorts. Neurologic Diseases in the Elderly Research Group. Neurology.

[131] Pezzoli G., Cereda E., Amami P., Colosimo S., Barichella M., Sacilotto G., Zecchinelli A., Zini M., Ferri V., Bolliri C. (2023). Onset and mortality of Parkinson’s disease in relation to type II diabetes. J. Neurol..

[132] Perruolo G., Viggiano D., Fiory F., Cassese A., Nigro C., Liotti A., Miele C., Beguinot F., Formisano P. (2016). Parkinson-like phenotype in insulin-resistant PED/PEA-15 transgenic mice. Sci. Rep..

[133] Martinez-Valbuena I., Amat-Villegas I., Valenti-Azcarate R., Carmona-Abellan M.D.M., Marcilla I., Tunon M.T., Luquin M.R. (2018). Interaction of amyloidogenic proteins in pancreatic beta cells from subjects with synucleinopathies. Acta Neuropathol..

[134] Hogg E., Athreya K., Basile C., Tan E.E., Kaminski J., Tagliati M. (2018). High Prevalence of Undiagnosed Insulin Resistance in Non-Diabetic Subjects with Parkinson’s Disease. J. Parkinsons Dis..

[135] Markaki I., Ntetsika T., Sorjonen K., Svenningsson P., BioPark Study G. (2021). Euglycemia Indicates Favorable Motor Outcome in Parkinson’s Disease. Mov. Disord..

[136] Zittel S., Uyar M., Lezius S., Gerloff C., Choe C.U. (2021). HbA1c and Motor Outcome in Parkinson’s Disease in the Mark-PD Study. Mov. Disord..

[137] Huxford B., Haque T., Joseph A.B., Simonet C., Gallagher D., Budu C., Dobson R., Noyce A. (2022). Parkinson’s Disease and Type 2 Diabetes: HbA1c Is Associated with Motor and Cognitive Severity. Mov. Disord..

[138] Konig A., Vicente Miranda H., Outeiro T.F. (2018). Alpha-Synuclein Glycation and the Action of Anti-Diabetic Agents in Parkinson’s Disease. J. Parkinsons Dis..

[139] Schernhammer E., Hansen J., Rugbjerg K., Wermuth L., Ritz B. (2011). Diabetes and the risk of developing Parkinson’s disease in Denmark. Diabetes Care.

[140] Brauer R., Wei L., Ma T., Athauda D., Girges C., Vijiaratnam N., Auld G., Whittlesea C., Wong I., Foltynie T. (2020). Diabetes medications and risk of Parkinson’s disease: A cohort study of patients with diabetes. Brain.

[141] Sanchez-Gomez A., Alcarraz-Vizan G., Fernandez M., Fernandez-Santiago R., Ezquerra M., Camara A., Serrano M., Novials A., Munoz E., Valldeoriola F. (2020). Peripheral insulin and amylin levels in Parkinson’s disease. Parkinsonism Relat. Disord..

[142] Rhee S.Y., Han K.D., Kwon H., Park S.E., Park Y.G., Kim Y.H., Yoo S.J., Rhee E.J., Lee W.Y. (2020). Association Between Glycemic Status and the Risk of Parkinson Disease: A Nationwide Population-Based Study. Diabetes Care.

[143] Athauda D., Maclagan K., Skene S.S., Bajwa-Joseph M., Letchford D., Chowdhury K., Hibbert S., Budnik N., Zampedri L., Dickson J. (2017). Exenatide once weekly versus placebo in Parkinson’s disease: A randomised, double-blind, placebo-controlled trial. Lancet.

[144] Kotagal V., Albin R.L., Muller M.L., Koeppe R.A., Frey K.A., Bohnen N.I. (2013). Diabetes is associated with postural instability and gait difficulty in Parkinson disease. Parkinsonism Relat. Disord..

[145] Malek N., Lawton M.A., Swallow D.M., Grosset K.A., Marrinan S.L., Bajaj N., Barker R.A., Burn D.J., Hardy J., Morris H.R. (2016). Vascular disease and vascular risk factors in relation to motor features and cognition in early Parkinson’s disease. Mov. Disord..

[146] Cereda E., Barichella M., Cassani E., Caccialanza R., Pezzoli G. (2012). Clinical features of Parkinson disease when onset of diabetes came first: A case-control study. Neurology.

[147] Mohamed Ibrahim N., Ramli R., Koya Kutty S., Shah S.A. (2018). Earlier onset of motor complications in Parkinson’s patients with comorbid diabetes mellitus. Mov. Disord..

[148] Mollenhauer B., Zimmermann J., Sixel-Doring F., Focke N.K., Wicke T., Ebentheuer J., Schaumburg M., Lang E., Friede T., Trenkwalder C. (2019). Baseline predictors for progression 4 years after Parkinson’s disease diagnosis in the De Novo Parkinson Cohort (DeNoPa). Mov. Disord..

[149] Ou R., Wei Q., Hou Y., Zhang L., Liu K., Lin J., Jiang Z., Song W., Cao B., Shang H. (2021). Effect of diabetes control status on the progression of Parkinson’s disease: A prospective study. Ann. Clin. Transl. Neurol..

[150] Athauda D., Evans J., Wernick A., Virdi G., Choi M.L., Lawton M., Vijiaratnam N., Girges C., Ben-Shlomo Y., Ismail K. (2022). The Impact of Type 2 Diabetes in Parkinson’s Disease. Mov. Disord..

[151] Barter J.D., Thomas D., Ni L., Bay A.A., Johnson T.M., Prusin T., Hackney M.E. (2023). Parkinson’s Disease and Diabetes Mellitus: Individual and Combined Effects on Motor, Cognitive, and Psychosocial Functions. Healthcare.

[152] Nair A.T., Ramachandran V., Joghee N.M., Antony S., Ramalingam G. (2018). Gut Microbiota Dysfunction as Reliable Non-invasive Early Diagnostic Biomarkers in the Pathophysiology of Parkinson’s Disease: A Critical Review. J. Neurogastroenterol. Motil..

[153] Cersosimo M.G., Benarroch E.E. (2012). Pathological correlates of gastrointestinal dysfunction in Parkinson’s disease. Neurobiol. Dis..

[154] Jones J.D., Rahmani E., Garcia E., Jacobs J.P. (2020). Gastrointestinal symptoms are predictive of trajectories of cognitive functioning in de novo Parkinson’s disease. Parkinsonism Relat. Disord..

[155] Williams-Gray C.H., Mason S.L., Evans J.R., Foltynie T., Brayne C., Robbins T.W., Barker R.A. (2013). The CamPaIGN study of Parkinson’s disease: 10-year outlook in an incident population-based cohort. J. Neurol. Neurosurg. Psychiatry.

[156] Caviness J.N., Driver-Dunckley E., Connor D.J., Sabbagh M.N., Hentz J.G., Noble B., Evidente V.G., Shill H.A., Adler C.H. (2007). Defining mild cognitive impairment in Parkinson’s disease. Mov. Disord..

[157] Janvin C.C., Larsen J.P., Aarsland D., Hugdahl K. (2006). Subtypes of mild cognitive impairment in Parkinson’s disease: Progression to dementia. Mov. Disord..

[158] Domellof M.E., Ekman U., Forsgren L., Elgh E. (2015). Cognitive function in the early phase of Parkinson’s disease, a five-year follow-up. Acta Neurol. Scand..

[159] Chapelet G., Leclair-Visonneau L., Clairembault T., Neunlist M., Derkinderen P. (2019). Can the gut be the missing piece in uncovering PD pathogenesis?. Parkinsonism Relat. Disord..

[160] Liang S., Wu X., Jin F. (2018). Gut-Brain Psychology: Rethinking Psychology From the Microbiota-Gut-Brain Axis. Front. Integr. Neurosci..

[161] Ransohoff R.M. (2016). How neuroinflammation contributes to neurodegeneration. Science.

[162] Sampson T.R., Debelius J.W., Thron T., Janssen S., Shastri G.G., Ilhan Z.E., Challis C., Schretter C.E., Rocha S., Gradinaru V. (2016). Gut Microbiota Regulate Motor Deficits and Neuroinflammation in a Model of Parkinson’s Disease. Cell.

[163] Hinkle J.T., Perepezko K., Mills K.A., Mari Z., Butala A., Dawson T.M., Pantelyat A., Rosenthal L.S., Pontone G.M. (2018). Dopamine transporter availability reflects gastrointestinal dysautonomia in early Parkinson disease. Parkinsonism Relat. Disord..

[164] Scheperjans F., Aho V., Pereira P.A., Koskinen K., Paulin L., Pekkonen E., Haapaniemi E., Kaakkola S., Eerola-Rautio J., Pohja M. (2015). Gut microbiota are related to Parkinson’s disease and clinical phenotype. Mov. Disord..

[165] Gareau M.G., Wine E., Rodrigues D.M., Cho J.H., Whary M.T., Philpott D.J., Macqueen G., Sherman P.M. (2011). Bacterial infection causes stress-induced memory dysfunction in mice. Gut.

[166] Clarke G., Grenham S., Scully P., Fitzgerald P., Moloney R.D., Shanahan F., Dinan T.G., Cryan J.F. (2013). The microbiome-gut-brain axis during early life regulates the hippocampal serotonergic system in a sex-dependent manner. Mol. Psychiatry.

[167] Devos D., Lebouvier T., Lardeux B., Biraud M., Rouaud T., Pouclet H., Coron E., Bruley des Varannes S., Naveilhan P., Nguyen J.M. (2013). Colonic inflammation in Parkinson’s disease. Neurobiol. Dis..

[168] Lindqvist D., Hall S., Surova Y., Nielsen H.M., Janelidze S., Brundin L., Hansson O. (2013). Cerebrospinal fluid inflammatory markers in Parkinson’s disease--associations with depression, fatigue, and cognitive impairment. Brain Behav. Immun..

[169] Yu S.Y., Zuo L.J., Wang F., Chen Z.J., Hu Y., Wang Y.J., Wang X.M., Zhang W. (2014). Potential biomarkers relating pathological proteins, neuroinflammatory factors and free radicals in PD patients with cognitive impairment: A cross-sectional study. BMC Neurol..

[170] Bohnen N.I., Kotagal V., Muller M.L., Koeppe R.A., Scott P.J., Albin R.L., Frey K.A., Petrou M. (2014). Diabetes mellitus is independently associated with more severe cognitive impairment in Parkinson disease. Parkinsonism Relat. Disord..

[171] Ong M., Foo H., Chander R.J., Wen M.C., Au W.L., Sitoh Y.Y., Tan L., Kandiah N. (2017). Influence of diabetes mellitus on longitudinal atrophy and cognition in Parkinson’s disease. J. Neurol. Sci..

[172] Petrou M., Davatzikos C., Hsieh M., Foerster B.R., Albin R.L., Kotagal V., Muller M.L., Koeppe R.A., Herman W.H., Frey K.A. (2016). Diabetes, Gray Matter Loss, and Cognition in the Setting of Parkinson Disease. Acad. Radiol..

[173] Gray M.T., Woulfe J.M. (2015). Striatal blood-brain barrier permeability in Parkinson’s disease. J. Cereb. Blood Flow Metab..

[174] Pienaar I.S., Lee C.H., Elson J.L., McGuinness L., Gentleman S.M., Kalaria R.N., Dexter D.T. (2015). Deep-brain stimulation associates with improved microvascular integrity in the subthalamic nucleus in Parkinson’s disease. Neurobiol. Dis..

[175] Elabi O.F., Cunha J., Gaceb A., Fex M., Paul G. (2021). High-fat diet-induced diabetes leads to vascular alterations, pericyte reduction, and perivascular depletion of microglia in a 6-OHDA toxin model of Parkinson disease. J. Neuroinflamm..

[176] Rom S., Heldt N.A., Gajghate S., Seliga A., Reichenbach N.L., Persidsky Y. (2020). Hyperglycemia and advanced glycation end products disrupt BBB and promote occludin and claudin-5 protein secretion on extracellular microvesicles. Sci. Rep..

[177] Takemoto M., Yamashita T., Ohta Y., Tadokoro K., Omote Y., Morihara R., Abe K. (2021). Cerebral Microbleeds in Patients with Parkinson’s Disease and Dementia with Lewy Bodies: Comparison Using Magnetic Resonance Imaging and 99 mTc-ECD SPECT Subtraction Imaging. J. Alzheimers Dis..

[178] Peelaerts W., Bousset L., Van der Perren A., Moskalyuk A., Pulizzi R., Giugliano M., Van den Haute C., Melki R., Baekelandt V. (2015). alpha-Synuclein strains cause distinct synucleinopathies after local and systemic administration. Nature.

[179] Biondetti E., Santin M.D., Valabregue R., Mangone G., Gaurav R., Pyatigorskaya N., Hutchison M., Yahia-Cherif L., Villain N., Habert M.O. (2021). The spatiotemporal changes in dopamine, neuromelanin and iron characterizing Parkinson’s disease. Brain.

[180] Mahoney-Sanchez L., Bouchaoui H., Ayton S., Devos D., Duce J.A., Devedjian J.C. (2021). Ferroptosis and its potential role in the physiopathology of Parkinson’s Disease. Prog. Neurobiol..

[181] Pyatigorskaya N., Sharman M., Corvol J.C., Valabregue R., Yahia-Cherif L., Poupon F., Cormier-Dequaire F., Siebner H., Klebe S., Vidailhet M. (2015). High nigral iron deposition in LRRK2 and Parkin mutation carriers using R2* relaxometry. Mov. Disord..

[182] Zhang L., Zhang L., Li Y., Li L., Melchiorsen J.U., Rosenkilde M., Holscher C. (2020). The Novel Dual GLP-1/GIP Receptor Agonist DA-CH5 Is Superior to Single GLP-1 Receptor Agonists in the MPTP Model of Parkinson’s Disease. J. Parkinsons Dis..

[183] Zhang P., Chen L., Zhao Q., Du X., Bi M., Li Y., Jiao Q., Jiang H. (2020). Ferroptosis was more initial in cell death caused by iron overload and its underlying mechanism in Parkinson’s disease. Free Radic. Biol. Med..

[184] DeFronzo R.A., Ferrannini E., Groop L., Henry R.R., Herman W.H., Holst J.J., Hu F.B., Kahn C.R., Raz I., Shulman G.I. (2015). Type 2 diabetes mellitus. Nat. Rev. Dis. Primers.

[185] Attwell D., Laughlin S.B. (2001). An energy budget for signaling in the grey matter of the brain. J. Cereb. Blood Flow Metab..

[186] Pissadaki E.K., Bolam J.P. (2013). The energy cost of action potential propagation in dopamine neurons: Clues to susceptibility in Parkinson’s disease. Front. Comput. Neurosci..

[187] Surmeier D.J., Obeso J.A., Halliday G.M. (2017). Selective neuronal vulnerability in Parkinson disease. Nat. Rev. Neurosci..

[188] Chatterjee S., Khunti K., Davies M.J. (2017). Type 2 diabetes. Lancet.

[189] Fearnley J.M., Lees A.J. (1991). Ageing and Parkinson’s disease: Substantia nigra regional selectivity. Brain.

[190] Parkkinen L., O’Sullivan S.S., Collins C., Petrie A., Holton J.L., Revesz T., Lees A.J. (2011). Disentangling the relationship between lewy bodies and nigral neuronal loss in Parkinson’s disease. J. Parkinsons Dis..

[191] Roberts R.F., Wade-Martins R., Alegre-Abarrategui J. (2015). Direct visualization of alpha-synuclein oligomers reveals previously undetected pathology in Parkinson’s disease brain. Brain.

[192] Cheong J.L.Y., de Pablo-Fernandez E., Foltynie T., Noyce A.J. (2020). The Association Between Type 2 Diabetes Mellitus and Parkinson’s Disease. J. Parkinsons Dis..

[193] Clark A., Nilsson M.R. (2004). Islet amyloid: A complication of islet dysfunction or an aetiological factor in Type 2 diabetes?. Diabetologia.

[194] Jackson K., Barisone G.A., Diaz E., Jin L.W., DeCarli C., Despa F. (2013). Amylin deposition in the brain: A second amyloid in Alzheimer disease?. Ann. Neurol..

[195] Banks W.A., Kastin A.J. (1998). Differential permeability of the blood-brain barrier to two pancreatic peptides: Insulin and amylin. Peptides.

[196] Horvath I., Wittung-Stafshede P. (2016). Cross-talk between amyloidogenic proteins in type-2 diabetes and Parkinson’s disease. Proc. Natl. Acad. Sci. USA.

[197] Verma N., Ly H., Liu M., Chen J., Zhu H., Chow M., Hersh L.B., Despa F. (2016). Intraneuronal Amylin Deposition, Peroxidative Membrane Injury and Increased IL-1beta Synthesis in Brains of Alzheimer’s Disease Patients with Type-2 Diabetes and in Diabetic HIP Rats. J. Alzheimers Dis..

[198] Ly H., Verma N., Wu F., Liu M., Saatman K.E., Nelson P.T., Slevin J.T., Goldstein L.B., Biessels G.J., Despa F. (2017). Brain microvascular injury and white matter disease provoked by diabetes-associated hyperamylinemia. Ann. Neurol..

[199] Oskarsson M.E., Paulsson J.F., Schultz S.W., Ingelsson M., Westermark P., Westermark G.T. (2015). In vivo seeding and cross-seeding of localized amyloidosis: A molecular link between type 2 diabetes and Alzheimer disease. Am. J. Pathol..

[200] Schultz N., Byman E., Fex M., Wennstrom M. (2017). Amylin alters human brain pericyte viability and NG2 expression. J. Cereb. Blood Flow. Metab..

[201] Martinez-Valbuena I., Valenti-Azcarate R., Amat-Villegas I., Riverol M., Marcilla I., de Andrea C.E., Sanchez-Arias J.A., Del Mar Carmona-Abellan M., Marti G., Erro M.E. (2019). Amylin as a potential link between type 2 diabetes and alzheimer disease. Ann. Neurol..

[202] Saller C.F., Chiodo L.A. (1980). Glucose suppresses basal firing and haloperidol-induced increases in the firing rate of central dopaminergic neurons. Science.

[203] Montefusco O., Assini M.C., Missale C. (1983). Insulin-mediated effects of glucose on dopamine metabolism. Acta Diabetol. Lat..

[204] Murzi E., Contreras Q., Teneud L., Valecillos B., Parada M.A., De Parada M.P., Hernandez L. (1996). Diabetes decreases limbic extracellular dopamine in rats. Neurosci. Lett..

[205] Renaud J., Bassareo V., Beaulieu J., Pinna A., Schlich M., Lavoie C., Murtas D., Simola N., Martinoli M.G. (2018). Dopaminergic neurodegeneration in a rat model of long-term hyperglycemia: Preferential degeneration of the nigrostriatal motor pathway. Neurobiol. Aging.

[206] Perez-Taboada I., Alberquilla S., Martin E.D., Anand R., Vietti-Michelina S., Tebeka N.N., Cantley J., Cragg S.J., Moratalla R., Vallejo M. (2020). Diabetes Causes Dysfunctional Dopamine Neurotransmission Favoring Nigrostriatal Degeneration in Mice. Mov. Disord..

[207] Su C.J., Shen Z., Cui R.X., Huang Y., Xu D.L., Zhao F.L., Pan J., Shi A.M., Liu T., Yu Y.L. (2020). Thioredoxin-Interacting Protein (TXNIP) Regulates Parkin/PINK1-mediated Mitophagy in Dopaminergic Neurons Under High-glucose Conditions: Implications for Molecular Links Between Parkinson’s Disease and Diabetes. Neurosci. Bull..

[208] Dionisio P.A., Amaral J.D., Rodrigues C.M.P. (2021). Oxidative stress and regulated cell death in Parkinson’s disease. Ageing Res. Rev..

[209] Chegao A., Guarda M., Alexandre B.M., Shvachiy L., Temido-Ferreira M., Marques-Morgado I., Fernandes Gomes B., Matthiesen R., Lopes L.V., Florindo P.R. (2022). Glycation modulates glutamatergic signaling and exacerbates Parkinson’s disease-like phenotypes. npj Parkinsons Dis..

[210] Vicente Miranda H., Szego E.M., Oliveira L.M.A., Breda C., Darendelioglu E., de Oliveira R.M., Ferreira D.G., Gomes M.A., Rott R., Oliveira M. (2017). Glycation potentiates alpha-synuclein-associated neurodegeneration in synucleinopathies. Brain.

[211] Xie B., Lin F., Peng L., Ullah K., Wu H., Qing H., Deng Y. (2014). Methylglyoxal increases dopamine level and leads to oxidative stress in SH-SY5Y cells. Acta Biochim. Biophys. Sin..

[212] Rowan S., Bejarano E., Taylor A. (2018). Mechanistic targeting of advanced glycation end-products in age-related diseases. Biochim. Biophys. Acta Mol. Basis Dis..

[213] Thornalley P.J., Langborg A., Minhas H.S. (1999). Formation of glyoxal, methylglyoxal and 3-deoxyglucosone in the glycation of proteins by glucose. Biochem. J..

[214] Shaikh S., Nicholson L.F. (2008). Advanced glycation end products induce in vitro cross-linking of alpha-synuclein and accelerate the process of intracellular inclusion body formation. J. Neurosci. Res..

[215] Du X.Y., Xie X.X., Liu R.T. (2020). The Role of alpha-Synuclein Oligomers in Parkinson’s Disease. Int. J. Mol. Sci..

[216] Uceda A.B., Frau J., Vilanova B., Adrover M. (2022). Glycation of alpha-synuclein hampers its binding to synaptic-like vesicles and its driving effect on their fusion. Cell. Mol. Life Sci..

[217] Ambrosi G., Cerri S., Blandini F. (2014). A further update on the role of excitotoxicity in the pathogenesis of Parkinson’s disease. J. Neural Transm..

[218] Xie B., Lin F., Ullah K., Peng L., Ding W., Dai R., Qing H., Deng Y. (2015). A newly discovered neurotoxin ADTIQ associated with hyperglycemia and Parkinson’s disease. Biochem. Biophys. Res. Commun..

[219] Castellani R., Smith M.A., Richey P.L., Perry G. (1996). Glycoxidation and oxidative stress in Parkinson disease and diffuse Lewy body disease. Brain Res..

[220] Munch G., Luth H.J., Wong A., Arendt T., Hirsch E., Ravid R., Riederer P. (2000). Crosslinking of alpha-synuclein by advanced glycation endproducts--an early pathophysiological step in Lewy body formation?. J. Chem. Neuroanat..

[221] Pearce R.K., Owen A., Daniel S., Jenner P., Marsden C.D. (1997). Alterations in the distribution of glutathione in the substantia nigra in Parkinson’s disease. J. Neural Transm..

[222] Kuhla B., Boeck K., Luth H.J., Schmidt A., Weigle B., Schmitz M., Ogunlade V., Munch G., Arendt T. (2006). Age-dependent changes of glyoxalase I expression in human brain. Neurobiol. Aging.

[223] Fusco G., Pape T., Stephens A.D., Mahou P., Costa A.R., Kaminski C.F., Kaminski Schierle G.S., Vendruscolo M., Veglia G., Dobson C.M. (2016). Structural basis of synaptic vesicle assembly promoted by alpha-synuclein. Nat. Commun..

[224] Nakayama K., Nakayama M., Iwabuchi M., Terawaki H., Sato T., Kohno M., Ito S. (2008). Plasma alpha-oxoaldehyde levels in diabetic and nondiabetic chronic kidney disease patients. Am. J. Nephrol..

[225] Beisswenger P.J., Drummond K.S., Nelson R.G., Howell S.K., Szwergold B.S., Mauer M. (2005). Susceptibility to diabetic nephropathy is related to dicarbonyl and oxidative stress. Diabetes.

[226] Nemet I., Turk Z., Duvnjak L., Car N., Varga-Defterdarovic L. (2005). Humoral methylglyoxal level reflects glycemic fluctuation. Clin. Biochem..

[227] Yaffe K., Lindquist K., Schwartz A.V., Vitartas C., Vittinghoff E., Satterfield S., Simonsick E.M., Launer L., Rosano C., Cauley J.A. (2011). Advanced glycation end product level, diabetes, and accelerated cognitive aging. Neurology.

[228] Schmidt A.M., Yan S.D., Yan S.F., Stern D.M. (2001). The multiligand receptor RAGE as a progression factor amplifying immune and inflammatory responses. J. Clin. Investig..

[229] Farzadfard A., Konig A., Petersen S.V., Nielsen J., Vasili E., Dominguez-Meijide A., Buell A.K., Outeiro T.F., Otzen D.E. (2022). Glycation modulates alpha-synuclein fibrillization kinetics: A sweet spot for inhibition. J. Biol. Chem..

[230] Wan Q., Xiong Z.G., Man H.Y., Ackerley C.A., Braunton J., Lu W.Y., Becker L.E., MacDonald J.F., Wang Y.T. (1997). Recruitment of functional GABA(A) receptors to postsynaptic domains by insulin. Nature.

[231] Arnold S.E., Arvanitakis Z., Macauley-Rambach S.L., Koenig A.M., Wang H.Y., Ahima R.S., Craft S., Gandy S., Buettner C., Stoeckel L.E. (2018). Brain insulin resistance in type 2 diabetes and Alzheimer disease: Concepts and conundrums. Nat. Rev. Neurol..

[232] Yao W.D., Gainetdinov R.R., Arbuckle M.I., Sotnikova T.D., Cyr M., Beaulieu J.M., Torres G.E., Grant S.G., Caron M.G. (2004). Identification of PSD-95 as a regulator of dopamine-mediated synaptic and behavioral plasticity. Neuron.

[233] Zhao W.Q., Chen H., Quon M.J., Alkon D.L. (2004). Insulin and the insulin receptor in experimental models of learning and memory. Eur. J. Pharmacol..

[234] Brunet A., Bonni A., Zigmond M.J., Lin M.Z., Juo P., Hu L.S., Anderson M.J., Arden K.C., Blenis J., Greenberg M.E. (1999). Akt promotes cell survival by phosphorylating and inhibiting a Forkhead transcription factor. Cell.

[235] Victorino D.B., Nejm M., Guimaraes-Marques M., Scorza F.A., Scorza C.A. (2021). Repurposing GLP-1 Receptor Agonists for Parkinson’s Disease: Current Evidence and Future Opportunities. Pharm Med..

[236] Yu H., Sun T., He X., Wang Z., Zhao K., An J., Wen L., Li J.Y., Li W., Feng J. (2022). Association between Parkinson’s Disease and Diabetes Mellitus: From Epidemiology, Pathophysiology and Prevention to Treatment. Aging Dis..

[237] Sabari S.S., Balasubramani K., Iyer M., Sureshbabu H.W., Venkatesan D., Gopalakrishnan A.V., Narayanaswamy A., Senthil Kumar N., Vellingiri B. (2023). Type 2 Diabetes (T2DM) and Parkinson’s Disease (PD): A Mechanistic Approach. Mol. Neurobiol..

[238] Lee S.H., Park S.Y., Choi C.S. (2022). Insulin Resistance: From Mechanisms to Therapeutic Strategies. Diabetes Metab. J..

[239] Nelson T.J., Sun M.K., Hongpaisan J., Alkon D.L. (2008). Insulin, PKC signaling pathways and synaptic remodeling during memory storage and neuronal repair. Eur. J. Pharmacol..

[240] van der Heide L.P., Ramakers G.M., Smidt M.P. (2006). Insulin signaling in the central nervous system: Learning to survive. Prog. Neurobiol..

[241] Ghasemi R., Haeri A., Dargahi L., Mohamed Z., Ahmadiani A. (2013). Insulin in the brain: Sources, localization and functions. Mol. Neurobiol..

[242] Uemura E., Greenlee H.W. (2006). Insulin regulates neuronal glucose uptake by promoting translocation of glucose transporter GLUT3. Exp. Neurol..

[243] Heidenrich K.A., Gilmore P.R., Garvey W.T. (1989). Glucose transport in primary cultured neurons. J. Neurosci. Res..

[244] Bak L.K., Walls A.B., Schousboe A., Ring A., Sonnewald U., Waagepetersen H.S. (2009). Neuronal glucose but not lactate utilization is positively correlated with NMDA-induced neurotransmission and fluctuations in cytosolic Ca2+ levels. J. Neurochem..

[245] Arbo B.D., Schimith L.E., Goulart Dos Santos M., Hort M.A. (2022). Repositioning and development of new treatments for neurodegenerative diseases: Focus on neuroinflammation. Eur. J. Pharmacol..

[246] Peineau S., Taghibiglou C., Bradley C., Wong T.P., Liu L., Lu J., Lo E., Wu D., Saule E., Bouschet T. (2007). LTP inhibits LTD in the hippocampus via regulation of GSK3beta. Neuron.

[247] Goldin M., Segal M. (2003). Protein kinase C and ERK involvement in dendritic spine plasticity in cultured rodent hippocampal neurons. Eur. J. Neurosci..

[248] Saravanan S., Ramkumar K., Adalarasu K., Sivanandam V., Kumar S.R., Stalin S., Amirtharajan R. (2022). A Systematic Review of Artificial Intelligence (AI) Based Approaches for the Diagnosis of Parkinson’s Disease. Arch. Comput. Methods Eng..

[249] Talbot K., Wang H.Y., Kazi H., Han L.Y., Bakshi K.P., Stucky A., Fuino R.L., Kawaguchi K.R., Samoyedny A.J., Wilson R.S. (2012). Demonstrated brain insulin resistance in Alzheimer’s disease patients is associated with IGF-1 resistance, IRS-1 dysregulation, and cognitive decline. J. Clin. Investig..

[250] Moloney A.M., Griffin R.J., Timmons S., O’Connor R., Ravid R., O’Neill C. (2010). Defects in IGF-1 receptor, insulin receptor and IRS-1/2 in Alzheimer’s disease indicate possible resistance to IGF-1 and insulin signalling. Neurobiol. Aging.

[251] Bassil F., Delamarre A., Canron M.H., Dutheil N., Vital A., Negrier-Leibreich M.L., Bezard E., Fernagut P.O., Meissner W.G. (2022). Impaired brain insulin signalling in Parkinson’s disease. Neuropathol. Appl. Neurobiol..

[252] Bassil F., Canron M.H., Vital A., Bezard E., Li Y., Greig N.H., Gulyani S., Kapogiannis D., Fernagut P.O., Meissner W.G. (2017). Insulin resistance and exendin-4 treatment for multiple system atrophy. Brain.

[253] Frolich L., Blum-Degen D., Bernstein H.G., Engelsberger S., Humrich J., Laufer S., Muschner D., Thalheimer A., Turk A., Hoyer S. (1998). Brain insulin and insulin receptors in aging and sporadic Alzheimer’s disease. J. Neural Transm..

[254] Moroo I., Yamada T., Makino H., Tooyama I., McGeer P.L., McGeer E.G., Hirayama K. (1994). Loss of insulin receptor immunoreactivity from the substantia nigra pars compacta neurons in Parkinson’s disease. Acta Neuropathol..

[255] Takahashi M., Yamada T., Tooyama I., Moroo I., Kimura H., Yamamoto T., Okada H. (1996). Insulin receptor mRNA in the substantia nigra in Parkinson’s disease. Neurosci. Lett..

[256] Athauda D., Foltynie T. (2016). Insulin resistance and Parkinson’s disease: A new target for disease modification?. Prog. Neurobiol..

[257] Hong C.T., Chen K.Y., Wang W., Chiu J.Y., Wu D., Chao T.Y., Hu C.J., Chau K.D., Bamodu O.A. (2020). Insulin Resistance Promotes Parkinson’s Disease through Aberrant Expression of alpha-Synuclein, Mitochondrial Dysfunction, and Deregulation of the Polo-Like Kinase 2 Signaling. Cells.

[258] Kleinridders A., Cai W., Cappellucci L., Ghazarian A., Collins W.R., Vienberg S.G., Pothos E.N., Kahn C.R. (2015). Insulin resistance in brain alters dopamine turnover and causes behavioral disorders. Proc. Natl. Acad. Sci. USA.

[259] Reale M., Iarlori C., Thomas A., Gambi D., Perfetti B., Di Nicola M., Onofrj M. (2009). Peripheral cytokines profile in Parkinson’s disease. Brain Behav. Immun..

[260] Brodacki B., Staszewski J., Toczylowska B., Kozlowska E., Drela N., Chalimoniuk M., Stepien A. (2008). Serum interleukin (IL-2, IL-10, IL-6, IL-4), TNFalpha, and INFgamma concentrations are elevated in patients with atypical and idiopathic parkinsonism. Neurosci. Lett..

[261] Sawada H., Oeda T., Umemura A., Tomita S., Kohsaka M., Park K., Yamamoto K., Sugiyama H. (2015). Baseline C-Reactive Protein Levels and Life Prognosis in Parkinson Disease. PLoS ONE.

[262] Williams-Gray C.H., Wijeyekoon R., Yarnall A.J., Lawson R.A., Breen D.P., Evans J.R., Cummins G.A., Duncan G.W., Khoo T.K., Burn D.J. (2016). Serum immune markers and disease progression in an incident Parkinson’s disease cohort (ICICLE-PD). Mov. Disord..

[263] Donath M.Y., Shoelson S.E. (2011). Type 2 diabetes as an inflammatory disease. Nat. Rev. Immunol..

[264] Sonnen J.A., Larson E.B., Brickell K., Crane P.K., Woltjer R., Montine T.J., Craft S. (2009). Different patterns of cerebral injury in dementia with or without diabetes. Arch. Neurol..

[265] Sulzer D., Alcalay R.N., Garretti F., Cote L., Kanter E., Agin-Liebes J., Liong C., McMurtrey C., Hildebrand W.H., Mao X. (2017). Erratum: T cells from patients with Parkinson’s disease recognize alpha-synuclein peptides. Nature.

[266] Lindestam Arlehamn C.S., Dhanwani R., Pham J., Kuan R., Frazier A., Rezende Dutra J., Phillips E., Mallal S., Roederer M., Marder K.S. (2020). alpha-Synuclein-specific T cell reactivity is associated with preclinical and early Parkinson’s disease. Nat. Commun..

[267] Tansey M.G., Wallings R.L., Houser M.C., Herrick M.K., Keating C.E., Joers V. (2022). Inflammation and immune dysfunction in Parkinson disease. Nat. Rev. Immunol..

[268] Lau E.Y.M., Carroll E.C., Callender L.A., Hood G.A., Berryman V., Pattrick M., Finer S., Hitman G.A., Ackland G.L., Henson S.M. (2019). Type 2 diabetes is associated with the accumulation of senescent T cells. Clin. Exp. Immunol..

[269] Spielman L.J., Bahniwal M., Little J.P., Walker D.G., Klegeris A. (2015). Insulin Modulates In Vitro Secretion of Cytokines and Cytotoxins by Human Glial Cells. Curr. Alzheimer Res..

[270] Hwang I.K., Choi J.H., Nam S.M., Park O.K., Yoo D.Y., Kim W., Yi S.S., Won M.H., Seong J.K., Yoon Y.S. (2014). Activation of microglia and induction of pro-inflammatory cytokines in the hippocampus of type 2 diabetic rats. Neurol. Res..

[271] Bartels T., De Schepper S., Hong S. (2020). Microglia modulate neurodegeneration in Alzheimer’s and Parkinson’s diseases. Science.

[272] Gispen W.H., Biessels G.J. (2000). Cognition and synaptic plasticity in diabetes mellitus. Trends Neurosci..

[273] Picconi B., De Leonibus E., Calabresi P. (2018). Synaptic plasticity and levodopa-induced dyskinesia: Electrophysiological and structural abnormalities. J. Neural. Transm..

[274] Morgante F., Espay A.J., Gunraj C., Lang A.E., Chen R. (2006). Motor cortex plasticity in Parkinson’s disease and levodopa-induced dyskinesias. Brain.

[275] De Felice F.G., Lourenco M.V., Ferreira S.T. (2014). How does brain insulin resistance develop in Alzheimer’s disease?. Alzheimer’s Dement..

[276] Blesa J., Trigo-Damas I., Quiroga-Varela A., Jackson-Lewis V.R. (2015). Oxidative stress and Parkinson’s disease. Front. Neuroanat..

[277] Burbulla L.F., Song P., Mazzulli J.R., Zampese E., Wong Y.C., Jeon S., Santos D.P., Blanz J., Obermaier C.D., Strojny C. (2017). Dopamine oxidation mediates mitochondrial and lysosomal dysfunction in Parkinson’s disease. Science.

[278] Musgrove R.E., Helwig M., Bae E.J., Aboutalebi H., Lee S.J., Ulusoy A., Di Monte D.A. (2019). Oxidative stress in vagal neurons promotes parkinsonian pathology and intercellular alpha-synuclein transfer. J. Clin. Investig..

[279] Scudamore O., Ciossek T. (2018). Increased Oxidative Stress Exacerbates alpha-Synuclein Aggregation In Vivo. J. Neuropathol. Exp. Neurol..

[280] Nalls M.A., Pankratz N., Lill C.M., Do C.B., Hernandez D.G., Saad M., DeStefano A.L., Kara E., Bras J., Sharma M. (2014). Large-scale meta-analysis of genome-wide association data identifies six new risk loci for Parkinson’s disease. Nat. Genet..

[281] Clarke D.W., Boyd F.T., Kappy M.S., Raizada M.K. (1984). Insulin binds to specific receptors and stimulates 2-deoxy-D-glucose uptake in cultured glial cells from rat brain. J. Biol. Chem..

[282] Ruegsegger G.N., Creo A.L., Cortes T.M., Dasari S., Nair K.S. (2018). Altered mitochondrial function in insulin-deficient and insulin-resistant states. J. Clin. Investig..

[283] Ruegsegger G.N., Vanderboom P.M., Dasari S., Klaus K.A., Kabiraj P., McCarthy C.B., Lucchinetti C.F., Nair K.S. (2019). Exercise and metformin counteract altered mitochondrial function in the insulin-resistant brain. JCI Insight.

[284] Schell M., Wardelmann K., Kleinridders A. (2021). Untangling the effect of insulin action on brain mitochondria and metabolism. J. Neuroendocrinol..

[285] Carvalho C., Santos M.S., Oliveira C.R., Moreira P.I. (2015). Alzheimer’s disease and type 2 diabetes-related alterations in brain mitochondria, autophagy and synaptic markers. Biochim. Biophys. Acta.

[286] Moreira P.I., Rolo A.P., Sena C., Seica R., Oliveira C.R., Santos M.S. (2006). Insulin attenuates diabetes-related mitochondrial alterations: A comparative study. Med. Chem..

[287] Carvalho C., Cardoso S., Correia S.C., Santos R.X., Santos M.S., Baldeiras I., Oliveira C.R., Moreira P.I. (2012). Metabolic alterations induced by sucrose intake and Alzheimer’s disease promote similar brain mitochondrial abnormalities. Diabetes.

[288] Raza H., John A., Howarth F.C. (2015). Increased oxidative stress and mitochondrial dysfunction in zucker diabetic rat liver and brain. Cell. Physiol. Biochem..

[289] Santos M.S., Santos D.L., Palmeira C.M., Seica R., Moreno A.J., Oliveira C.R. (2001). Brain and liver mitochondria isolated from diabetic Goto-Kakizaki rats show different susceptibility to induced oxidative stress. Diabetes Metab. Res. Rev..

[290] Gonzalez-Rodriguez P., Zampese E., Stout K.A., Guzman J.N., Ilijic E., Yang B., Tkatch T., Stavarache M.A., Wokosin D.L., Gao L. (2021). Disruption of mitochondrial complex I induces progressive parkinsonism. Nature.

[291] Santos R.X., Correia S.C., Alves M.G., Oliveira P.F., Cardoso S., Carvalho C., Seica R., Santos M.S., Moreira P.I. (2014). Mitochondrial quality control systems sustain brain mitochondrial bioenergetics in early stages of type 2 diabetes. Mol. Cell. Biochem..

[292] Schalkwijk C.G., Stehouwer C.D.A. (2020). Methylglyoxal, a Highly Reactive Dicarbonyl Compound, in Diabetes, Its Vascular Complications, and Other Age-Related Diseases. Physiol. Rev..

[293] Hou X., Watzlawik J.O., Fiesel F.C., Springer W. (2020). Autophagy in Parkinson’s Disease. J. Mol. Biol..

[294] Kong F.J., Ma L.L., Guo J.J., Xu L.H., Li Y., Qu S. (2018). Endoplasmic reticulum stress/autophagy pathway is involved in diabetes-induced neuronal apoptosis and cognitive decline in mice. Clin. Sci.

[295] Chen J.L., Luo C., Pu D., Zhang G.Q., Zhao Y.X., Sun Y., Zhao K.X., Liao Z.Y., Lv A.K., Zhu S.Y. (2019). Metformin attenuates diabetes-induced tau hyperphosphorylation in vitro and in vivo by enhancing autophagic clearance. Exp. Neurol..

[296] Guan Z.F., Tao Y.H., Zhang X.M., Guo Q.L., Liu Y.C., Zhang Y., Wang Y.M., Ji G., Wu G.F., Wang N.N. (2017). G-CSF and cognitive dysfunction in elderly diabetic mice with cerebral small vessel disease: Preventive intervention effects and underlying mechanisms. CNS Neurosci. Ther..

[297] Guan Z.F., Zhou X.L., Zhang X.M., Zhang Y., Wang Y.M., Guo Q.L., Ji G., Wu G.F., Wang N.N., Yang H. (2016). Beclin-1- mediated autophagy may be involved in the elderly cognitive and affective disorders in streptozotocin-induced diabetic mice. Transl. Neurodegener..

[298] Jing Y.H., Zhang L., Gao L.P., Qi C.C., Lv D.D., Song Y.F., Yin J., Wang D.G. (2017). Autophagy plays beneficial effect on diabetic encephalopathy in type 2 diabetes: Studies in vivo and in vitro. Neuroendocrinol. Lett..

[299] Li Y., Zhang Y., Wang L., Wang P., Xue Y., Li X., Qiao X., Zhang X., Xu T., Liu G. (2017). Autophagy impairment mediated by S-nitrosation of ATG4B leads to neurotoxicity in response to hyperglycemia. Autophagy.

[300] Codogno P., Meijer A.J. (2005). Autophagy and signaling: Their role in cell survival and cell death. Cell Death Differ..

[301] Mammucari C., Milan G., Romanello V., Masiero E., Rudolf R., Del Piccolo P., Burden S.J., Di Lisi R., Sandri C., Zhao J. (2007). FoxO3 controls autophagy in skeletal muscle in vivo. Cell Metab..

[302] Heras-Sandoval D., Perez-Rojas J.M., Hernandez-Damian J., Pedraza-Chaverri J. (2014). The role of PI3K/AKT/mTOR pathway in the modulation of autophagy and the clearance of protein aggregates in neurodegeneration. Cell. Signal.

[303] Pignalosa F.C., Desiderio A., Mirra P., Nigro C., Perruolo G., Ulianich L., Formisano P., Beguinot F., Miele C., Napoli R. (2021). Diabetes and Cognitive Impairment: A Role for Glucotoxicity and Dopaminergic Dysfunction. Int. J. Mol. Sci..

[304] Lv Y.Q., Yuan L., Sun Y., Dou H.W., Su J.H., Hou Z.P., Li J.Y., Li W. (2022). Long-term hyperglycemia aggravates alpha-synuclein aggregation and dopaminergic neuronal loss in a Parkinson’s disease mouse model. Transl. Neurodegener..

[305] Su C.J., Feng Y., Liu T.T., Liu X., Bao J.J., Shi A.M., Hu D.M., Liu T., Yu Y.L. (2017). Thioredoxin-interacting protein induced alpha-synuclein accumulation via inhibition of autophagic flux: Implications for Parkinson’s disease. CNS Neurosci. Ther..

[306] Chen L., Ding Y., Cagniard B., Van Laar A.D., Mortimer A., Chi W., Hastings T.G., Kang U.J., Zhuang X. (2008). Unregulated cytosolic dopamine causes neurodegeneration associated with oxidative stress in mice. J. Neurosci..

[307] Hijaz B.A., Volpicelli-Daley L.A. (2020). Initiation and propagation of alpha-synuclein aggregation in the nervous system. Mol. Neurodegener..

[308] Olanow C.W. (2019). Levodopa is the best symptomatic therapy for PD: Nothing more, nothing less. Mov. Disord..

[309] Kalia L.V., Lang A.E. (2015). Parkinson’s disease. Lancet.

[310] Dexter D.T., Wells F.R., Agid F., Agid Y., Lees A.J., Jenner P., Marsden C.D. (1987). Increased nigral iron content in postmortem parkinsonian brain. Lancet.

[311] International Parkinson’s Disease Genomics Consortium, Wellcome Trust Case Control Consortium 2 (2011). A two-stage meta-analysis identifies several new loci for Parkinson’s disease. PLoS Genet..

[312] Halliday G.M., Stevens C.H. (2011). Glia: Initiators and progressors of pathology in Parkinson’s disease. Mov. Disord..

[313] Imamura K., Hishikawa N., Sawada M., Nagatsu T., Yoshida M., Hashizume Y. (2003). Distribution of major histocompatibility complex class II-positive microglia and cytokine profile of Parkinson’s disease brains. Acta Neuropathol..

[314] Exner N., Lutz A.K., Haass C., Winklhofer K.F. (2012). Mitochondrial dysfunction in Parkinson’s disease: Molecular mechanisms and pathophysiological consequences. EMBO J..

[315] Chen S., Yu S.J., Li Y., Lecca D., Glotfelty E., Kim H.K., Choi H.I., Hoffer B.J., Greig N.H., Kim D.S. (2018). Post-treatment with PT302, a long-acting Exendin-4 sustained release formulation, reduces dopaminergic neurodegeneration in a 6-Hydroxydopamine rat model of Parkinson’s disease. Sci. Rep..

[316] Zhang L., Zhang L., Li L., Holscher C. (2019). Semaglutide is Neuroprotective and Reduces alpha-Synuclein Levels in the Chronic MPTP Mouse Model of Parkinson’s Disease. J. Parkinsons Dis..

[317] Vijiaratnam N., Simuni T., Bandmann O., Morris H.R., Foltynie T. (2021). Progress towards therapies for disease modification in Parkinson’s disease. Lancet Neurol..

[318] Jankovic J. (2008). Parkinson’s disease: Clinical features and diagnosis. J. Neurol. Neurosurg. Psychiatry.

[319] Samii A., Nutt J.G., Ransom B.R. (2004). Parkinson’s disease. Lancet.

[320] Sveinbjornsdottir S. (2016). The clinical symptoms of Parkinson’s disease. J. Neurochem..

[321] AlDakheel A., Kalia L.V., Lang A.E. (2014). Pathogenesis-targeted, disease-modifying therapies in Parkinson disease. Neurotherapeutics.

[322] Connolly B.S., Lang A.E. (2014). Pharmacological treatment of Parkinson disease: A review. JAMA.

[323] National Collaborating Centre for Chronic Conditions (2006). National Collaborating Centre for Chronic, C. National Institute for Health and Clinical Excellence: Guidance. Parkinson’s Disease: National Clinical Guideline for Diagnosis and Management in Primary and Secondary Care.

[324] Armstrong M.J., Okun M.S. (2020). Diagnosis and Treatment of Parkinson Disease: A Review. JAMA.

[325] Lotti V.J., Porter C.C. (1970). Potentiation and inhbition of some central actions of L(-)-dopa by decarboxylase inhibitors. J. Pharmacol. Exp. Ther..

[326] Silva M.A., Mattern C., Hacker R., Tomaz C., Huston J.P., Schwarting R.K. (1997). Increased neostriatal dopamine activity after intraperitoneal or intranasal administration of L-DOPA: On the role of benserazide pretreatment. Synapse.

[327] Nord M. (2017). Levodopa Pharmacokinetics-From Stomach to Brain: A Study on Patients with Parkinson’s Disease. Ph.D. Thesis.

[328] Oertel W.H. (2017). Recent advances in treating Parkinson’s disease. F1000Research.

[329] Goldenberg M.M. (2008). Medical management of Parkinson’s disease. Pharm. Ther..

[330] Atmaca M. (2014). Drug-induced impulse control disorders: A review. Curr. Clin. Pharmacol..

[331] Baumann-Vogel H., Valko P.O., Eisele G., Baumann C.R. (2015). Impulse control disorders in Parkinson’s disease: Don’t set your mind at rest by self-assessments. Eur. J. Neurol..

[332] Moore T.J., Glenmullen J., Mattison D.R. (2014). Reports of pathological gambling, hypersexuality, and compulsive shopping associated with dopamine receptor agonist drugs. JAMA Intern. Med..

[333] Saez-Francas N., Marti Andres G., Ramirez N., de Fabregues O., Alvarez-Sabin J., Casas M., Hernandez-Vara J. (2016). Clinical and psychopathological factors associated with impulse control disorders in Parkinson’s disease. Neurologia.

[334] Seeman P. (2015). Parkinson’s disease treatment may cause impulse-control disorder via dopamine D3 receptors. Synapse.

[335] van Eimeren T., Ballanger B., Pellecchia G., Miyasaki J.M., Lang A.E., Strafella A.P. (2009). Dopamine agonists diminish value sensitivity of the orbitofrontal cortex: A trigger for pathological gambling in Parkinson’s disease?. Neuropsychopharmacology.

[336] Weintraub D., Claassen D.O. (2017). Impulse Control and Related Disorders in Parkinson’s Disease. Int. Rev. Neurobiol..

[337] Rascol O., Fabbri M., Poewe W. (2021). Amantadine in the treatment of Parkinson’s disease and other movement disorders. Lancet Neurol..

[338] Sawada H., Oeda T., Kuno S., Nomoto M., Yamamoto K., Yamamoto M., Hisanaga K., Kawamura T., Amantadine Study G. (2010). Amantadine for dyskinesias in Parkinson’s disease: A randomized controlled trial. PLoS ONE.

[339] Shin M.S., Kim T.W., Lee J.M., Ji E.S., Lim B.V. (2017). Treadmill exercise alleviates nigrostriatal dopaminergic loss of neurons and fibers in rotenone-induced Parkinson rats. J. Exerc. Rehabil..

[340] Lau Y.S., Patki G., Das-Panja K., Le W.D., Ahmad S.O. (2011). Neuroprotective effects and mechanisms of exercise in a chronic mouse model of Parkinson’s disease with moderate neurodegeneration. Eur. J. Neurosci..

[341] Sleiman S.F., Henry J., Al-Haddad R., El Hayek L., Abou Haidar E., Stringer T., Ulja D., Karuppagounder S.S., Holson E.B., Ratan R.R. (2016). Exercise promotes the expression of brain derived neurotrophic factor (BDNF) through the action of the ketone body beta-hydroxybutyrate. eLife.

[342] Fang X., Han D., Cheng Q., Zhang P., Zhao C., Min J., Wang F. (2018). Association of Levels of Physical Activity With Risk of Parkinson Disease: A Systematic Review and Meta-analysis. JAMA Netw. Open.

[343] Tsukita K., Sakamaki-Tsukita H., Takahashi R. (2022). Long-term Effect of Regular Physical Activity and Exercise Habits in Patients with Early Parkinson Disease. Neurology.

[344] van der Kolk N.M., de Vries N.M., Kessels R.P.C., Joosten H., Zwinderman A.H., Post B., Bloem B.R. (2019). Effectiveness of home-based and remotely supervised aerobic exercise in Parkinson’s disease: A double-blind, randomised controlled trial. Lancet Neurol..

[345] Johansson M.E., Cameron I.G.M., Van der Kolk N.M., de Vries N.M., Klimars E., Toni I., Bloem B.R., Helmich R.C. (2022). Aerobic Exercise Alters Brain Function and Structure in Parkinson’s Disease: A Randomized Controlled Trial. Ann. Neurol..

[346] Schenkman M., Moore C.G., Kohrt W.M., Hall D.A., Delitto A., Comella C.L., Josbeno D.A., Christiansen C.L., Berman B.D., Kluger B.M. (2018). Effect of High-Intensity Treadmill Exercise on Motor Symptoms in Patients With De Novo Parkinson Disease: A Phase 2 Randomized Clinical Trial. JAMA Neurol..

[347] Hu F.B., Manson J.E., Stampfer M.J., Colditz G., Liu S., Solomon C.G., Willett W.C. (2001). Diet, lifestyle, and the risk of type 2 diabetes mellitus in women. N. Engl. J. Med..

[348] Schellenberg E.S., Dryden D.M., Vandermeer B., Ha C., Korownyk C. (2013). Lifestyle interventions for patients with and at risk for type 2 diabetes: A systematic review and meta-analysis. Ann. Intern. Med..

[349] Ryan B.J., Schleh M.W., Ahn C., Ludzki A.C., Gillen J.B., Varshney P., Van Pelt D.W., Pitchford L.M., Chenevert T.L., Gioscia-Ryan R.A. (2020). Moderate-Intensity Exercise and High-Intensity Interval Training Affect Insulin Sensitivity Similarly in Obese Adults. J. Clin. Endocrinol. Metab..

[350] Holscher C. (2020). Brain insulin resistance: Role in neurodegenerative disease and potential for targeting. Expert. Opin. Investig. Drugs.

[351] Piancone F., La Rosa F., Marventano I., Saresella M., Clerici M. (2021). The Role of the Inflammasome in Neurodegenerative Diseases. Molecules.

[352] Labandeira C.M., Fraga-Bau A., Arias Ron D., Munoz A., Alonso-Losada G., Koukoulis A., Romero-Lopez J., Rodriguez-Perez A.I. (2021). Diabetes, insulin and new therapeutic strategies for Parkinson’s disease: Focus on glucagon-like peptide-1 receptor agonists. Front. Neuroendocrinol..

[353] Nowell J., Blunt E., Gupta D., Edison P. (2023). Antidiabetic agents as a novel treatment for Alzheimer’s and Parkinson’s disease. Ageing Res. Rev..

[354] Blevins H.M., Xu Y., Biby S., Zhang S. (2022). The NLRP3 Inflammasome Pathway: A Review of Mechanisms and Inhibitors for the Treatment of Inflammatory Diseases. Front. Aging Neurosci..

[355] Mucibabic M., Steneberg P., Lidh E., Straseviciene J., Ziolkowska A., Dahl U., Lindahl E., Edlund H. (2020). alpha-Synuclein promotes IAPP fibril formation in vitro and beta-cell amyloid formation in vivo in mice. Sci. Rep..

[356] Marwarha G., Rhen T., Schommer T., Ghribi O. (2011). The oxysterol 27-hydroxycholesterol regulates alpha-synuclein and tyrosine hydroxylase expression levels in human neuroblastoma cells through modulation of liver X receptors and estrogen receptors--relevance to Parkinson’s disease. J. Neurochem..

[357] Schommer J., Marwarha G., Schommer T., Flick T., Lund J., Ghribi O. (2018). 27-Hydroxycholesterol increases alpha-synuclein protein levels through proteasomal inhibition in human dopaminergic neurons. BMC Neurosci..

[358] García-Sanz P., Aerts J.M.F.G., Moratalla R. (2021). The Role of Cholesterol in alpha-Synuclein and Lewy Body Pathology in GBA1 Parkinson’s Disease. Mov. Disord..

[359] Hsiao J.T., Halliday G.M., Kim W.S. (2017). alpha-Synuclein Regulates Neuronal Cholesterol Efflux. Molecules.

[360] Jakubec M., Barias E., Furse S., Govasli M.L., George V., Turcu D., Iashchishyn I.A., Morozova-Roche L.A., Halskau O. (2021). Cholesterol-containing lipid nanodiscs promote an alpha-synuclein binding mode that accelerates oligomerization. FEBS J..

[361] Doria M., Maugest L., Moreau T., Lizard G., Vejux A. (2016). Contribution of cholesterol and oxysterols to the pathophysiology of Parkinson’s disease. Free Radic. Biol. Med..

[362] Bate C., Williams A. (2015). alpha-Synuclein-induced synapse damage in cultured neurons is mediated by cholesterol-sensitive activation of cytoplasmic phospholipase A2. Biomolecules.

[363] Schneeberger A., Tierney L., Mandler M. (2016). Active immunization therapies for Parkinson’s disease and multiple system atrophy. Mov. Disord..

[364] Volc D., Poewe W., Kutzelnigg A., Luhrs P., Thun-Hohenstein C., Schneeberger A., Galabova G., Majbour N., Vaikath N., El-Agnaf O. (2020). Safety and immunogenicity of the alpha-synuclein active immunotherapeutic PD01A in patients with Parkinson’s disease: A randomised, single-blinded, phase 1 trial. Lancet Neurol..

[365] Pagano G., Taylor K.I., Anzures-Cabrera J., Marchesi M., Simuni T., Marek K., Postuma R.B., Pavese N., Stocchi F., Azulay J.P. (2022). Trial of Prasinezumab in Early-Stage Parkinson’s Disease. N. Engl. J. Med..

[366] Lang A.E., Siderowf A.D., Macklin E.A., Poewe W., Brooks D.J., Fernandez H.H., Rascol O., Giladi N., Stocchi F., Tanner C.M. (2022). Trial of Cinpanemab in Early Parkinson’s Disease. N. Engl. J. Med..

[367] Hutchison R.M., Fraser K., Yang M., Fox T., Hirschhorn E., Njingti E., Scott D., Bedell B.J., Kistner K.M., Cedarbaum J.M. (2024). Cinpanemab in Early Parkinson Disease: Evaluation of Biomarker Results from the Phase 2 SPARK Clinical Trial. Neurology.

[368] Ionica L.N., Gaita L., Bina A.M., Sosdean R., Lighezan R., Sima A., Malita D., Cretu O.M., Burlacu O., Muntean D.M. (2021). Metformin alleviates monoamine oxidase-related vascular oxidative stress and endothelial dysfunction in rats with diet-induced obesity. Mol. Cell. Biochem..

[369] Kelly B., Tannahill G.M., Murphy M.P., O’Neill L.A. (2015). Metformin Inhibits the Production of Reactive Oxygen Species from NADH:Ubiquinone Oxidoreductase to Limit Induction of Interleukin-1beta (IL-1beta) and Boosts Interleukin-10 (IL-10) in Lipopolysaccharide (LPS)-activated Macrophages. J. Biol. Chem..

[370] Bharath L.P., Nikolajczyk B.S. (2021). The intersection of metformin and inflammation. Am. J. Physiol. Cell Physiol..

[371] Soberanes S., Misharin A.V., Jairaman A., Morales-Nebreda L., McQuattie-Pimentel A.C., Cho T., Hamanaka R.B., Meliton A.Y., Reyfman P.A., Walter J.M. (2019). Metformin Targets Mitochondrial Electron Transport to Reduce Air-Pollution-Induced Thrombosis. Cell Metab..

[372] Moiseeva O., Deschenes-Simard X., St-Germain E., Igelmann S., Huot G., Cadar A.E., Bourdeau V., Pollak M.N., Ferbeyre G. (2013). Metformin inhibits the senescence-associated secretory phenotype by interfering with IKK/NF-kappaB activation. Aging Cell.

[373] Shi Q., Liu S., Fonseca V.A., Thethi T.K., Shi L. (2019). Effect of metformin on neurodegenerative disease among elderly adult US veterans with type 2 diabetes mellitus. BMJ Open.

[374] Qin X., Zhang X., Li P., Wang M., Yan L., Bao Z., Liu Q. (2021). Association Between Diabetes Medications and the Risk of Parkinson’s Disease: A Systematic Review and Meta-Analysis. Front. Neurol..

[375] Kuan Y.C., Huang K.W., Lin C.L., Hu C.J., Kao C.H. (2017). Effects of metformin exposure on neurodegenerative diseases in elderly patients with type 2 diabetes mellitus. Prog. Neuropsychopharmacol. Biol. Psychiatry.

[376] Ping F., Jiang N., Li Y. (2020). Association between metformin and neurodegenerative diseases of observational studies: Systematic review and meta-analysis. BMJ Open Diabetes Res. Care.

[377] Baetta R., Corsini A. (2011). Pharmacology of dipeptidyl peptidase-4 inhibitors: Similarities and differences. Drugs.

[378] Capuano A., Sportiello L., Maiorino M.I., Rossi F., Giugliano D., Esposito K. (2013). Dipeptidyl peptidase-4 inhibitors in type 2 diabetes therapy—Focus on alogliptin. Drug Des. Dev. Ther..

[379] Chen S., Zhou M., Sun J., Guo A., Fernando R.L., Chen Y., Peng P., Zhao G., Deng Y. (2019). DPP-4 inhibitor improves learning and memory deficits and AD-like neurodegeneration by modulating the GLP-1 signaling. Neuropharmacology.

[380] Cheng Q., Cheng J., Cordato D., Gao J. (2020). Can dipeptidyl peptidase-4 inhibitors treat cognitive disorders?. Pharmacol. Ther..

[381] Yossef R.R., Al-Yamany M.F., Saad M.A., El-Sahar A.E. (2020). Neuroprotective effects of vildagliptin on drug induced Alzheimer’s disease in rats with metabolic syndrome: Role of hippocampal klotho and AKT signaling pathways. Eur. J. Pharmacol..

[382] Abdelsalam R.M., Safar M.M. (2015). Neuroprotective effects of vildagliptin in rat rotenone Parkinson’s disease model: Role of RAGE-NFkappaB and Nrf2-antioxidant signaling pathways. J. Neurochem..

[383] Badawi G.A., Abd El Fattah M.A., Zaki H.F., El Sayed M.I. (2017). Sitagliptin and liraglutide reversed nigrostriatal degeneration of rodent brain in rotenone-induced Parkinson’s disease. Inflammopharmacology.

[384] Kabel A.M., Omar M.S., Alhadhrami A., Alharthi S.S., Alrobaian M.M. (2018). Linagliptin potentiates the effect of l-dopa on the behavioural, biochemical and immunohistochemical changes in experimentally-induced Parkinsonism: Role of toll-like receptor 4, TGF-beta1, NF-kappaB and glucagon-like peptide 1. Physiol. Behav..

[385] Li J., Zhang S., Li C., Li M., Ma L. (2018). Sitagliptin rescues memory deficits in Parkinsonian rats via upregulating BDNF to prevent neuron and dendritic spine loss. Neurol. Res..

[386] Nassar N.N., Al-Shorbagy M.Y., Arab H.H., Abdallah D.M. (2015). Saxagliptin: A novel antiparkinsonian approach. Neuropharmacology.

[387] Badawi G.A., Abd El Fattah M.A., Zaki H.F., El Sayed M.I. (2019). Sitagliptin and Liraglutide Modulate L-dopa Effect and Attenuate Dyskinetic Movements in Rotenone-Lesioned Rats. Neurotox. Res..

[388] Svenningsson P., Wirdefeldt K., Yin L., Fang F., Markaki I., Efendic S., Ludvigsson J.F. (2016). Reduced incidence of Parkinson’s disease after dipeptidyl peptidase-4 inhibitors-A nationwide case-control study. Mov. Disord..

[389] Jeong S.H., Chung S.J., Yoo H.S., Hong N., Jung J.H., Baik K., Lee Y.H., Sohn Y.H., Lee P.H. (2021). Beneficial effects of dipeptidyl peptidase-4 inhibitors in diabetic Parkinson’s disease. Brain.

[390] Ates Bulut E., Sahin Alak Z.Y., Dokuzlar O., Kocyigit S.E., Soysal P., Smith L., Isik A.T. (2020). Cognitive and metabolic outcomes of vildagliptin addition to the therapy in patients with type 2 diabetes mellitus: 26 week follow-up study. Arch. Gerontol. Geriatr..

[391] Borzi A.M., Condorelli G., Biondi A., Basile F., Vicari E.S.D., Buscemi C., Luca S., Vacante M. (2019). Effects of vildagliptin, a DPP-4 inhibitor, in elderly diabetic patients with mild cognitive impairment. Arch. Gerontol. Geriatr..

[392] Rizzo M.R., Barbieri M., Boccardi V., Angellotti E., Marfella R., Paolisso G. (2014). Dipeptidyl peptidase-4 inhibitors have protective effect on cognitive impairment in aged diabetic patients with mild cognitive impairment. J. Gerontol. A Biol. Sci. Med. Sci..

[393] Harati M., Tayarani-Najaran Z., Javadi B. (2023). Dietary flavonoids: Promising compounds for targeting α-synucleinopathy in Parkinson’s disease. PharmaNutrition.

[394] Nauck M.A., Quast D.R., Wefers J., Meier J.J. (2021). GLP-1 receptor agonists in the treatment of type 2 diabetes—State-of-the-art. Mol. Metab..

[395] Muller T.D., Finan B., Bloom S.R., D’Alessio D., Drucker D.J., Flatt P.R., Fritsche A., Gribble F., Grill H.J., Habener J.F. (2019). Glucagon-like peptide 1 (GLP-1). Mol. Metab..

[396] Grieco M., Giorgi A., Gentile M.C., d’Erme M., Morano S., Maras B., Filardi T. (2019). Glucagon-Like Peptide-1: A Focus on Neurodegenerative Diseases. Front. Neurosci..

[397] Drucker D.J. (2018). Mechanisms of Action and Therapeutic Application of Glucagon-like Peptide-1. Cell Metab..

[398] Batista A.F., Bodart-Santos V., De Felice F.G., Ferreira S.T. (2019). Neuroprotective Actions of Glucagon-Like Peptide-1 (GLP-1) Analogues in Alzheimer’s and Parkinson’s Diseases. CNS Drugs.

[399] Glotfelty E.J., Olson L., Karlsson T.E., Li Y., Greig N.H. (2020). Glucagon-like peptide-1 (GLP-1)-based receptor agonists as a treatment for Parkinson’s disease. Expert. Opin. Investig. Drugs.

[400] Kim S., Moon M., Park S. (2009). Exendin-4 protects dopaminergic neurons by inhibition of microglial activation and matrix metalloproteinase-3 expression in an animal model of Parkinson’s disease. J. Endocrinol..

[401] Li Y., Perry T., Kindy M.S., Harvey B.K., Tweedie D., Holloway H.W., Powers K., Shen H., Egan J.M., Sambamurti K. (2009). GLP-1 receptor stimulation preserves primary cortical and dopaminergic neurons in cellular and rodent models of stroke and Parkinsonism. Proc. Natl. Acad. Sci. USA.

[402] Liu W., Jalewa J., Sharma M., Li G., Li L., Holscher C. (2015). Neuroprotective effects of lixisenatide and liraglutide in the 1-methyl-4-phenyl-1,2,3,6-tetrahydropyridine mouse model of Parkinson’s disease. Neuroscience.

[403] Zhang L., Zhang L., Li L., Holscher C. (2018). Neuroprotective effects of the novel GLP-1 long acting analogue semaglutide in the MPTP Parkinson’s disease mouse model. Neuropeptides.

[404] Bertilsson G., Patrone C., Zachrisson O., Andersson A., Dannaeus K., Heidrich J., Kortesmaa J., Mercer A., Nielsen E., Ronnholm H. (2008). Peptide hormone exendin-4 stimulates subventricular zone neurogenesis in the adult rodent brain and induces recovery in an animal model of Parkinson’s disease. J. Neurosci. Res..

[405] Lin T.K., Lin K.J., Lin H.Y., Lin K.L., Lan M.Y., Wang P.W., Wang T.J., Wang F.S., Tsai P.C., Liou C.W. (2021). Glucagon-Like Peptide-1 Receptor Agonist Ameliorates 1-Methyl-4-Phenyl-1,2,3,6-Tetrahydropyridine (MPTP) Neurotoxicity Through Enhancing Mitophagy Flux and Reducing alpha-Synuclein and Oxidative Stress. Front. Mol. Neurosci..

[406] Aviles-Olmos I., Dickson J., Kefalopoulou Z., Djamshidian A., Kahan J., Ell P., Whitton P., Wyse R., Isaacs T., Lees A. (2014). Motor and cognitive advantages persist 12 months after exenatide exposure in Parkinson’s disease. J. Parkinsons Dis..

[407] Athauda D., Maclagan K., Budnik N., Zampedri L., Hibbert S., Skene S.S., Chowdhury K., Aviles-Olmos I., Limousin P., Foltynie T. (2018). What Effects Might Exenatide have on Non-Motor Symptoms in Parkinson’s Disease: A Post Hoc Analysis. J. Parkinsons Dis..

[408] Nakamura K., Mori F., Tanji K., Miki Y., Yamada M., Kakita A., Takahashi H., Utsumi J., Sasaki H., Wakabayashi K. (2015). Isopentenyl diphosphate isomerase, a cholesterol synthesizing enzyme, is localized in Lewy bodies. Neuropathology.

[409] Mutez E., Duhamel A., Defebvre L., Bordet R., Destee A., Kreisler A. (2009). Lipid-lowering drugs are associated with delayed onset and slower course of Parkinson’s disease. Pharmacol. Res..

[410] Friedman B., Lahad A., Dresner Y., Vinker S. (2013). Long-term statin use and the risk of Parkinson’s disease. Am. J. Manag. Care.

[411] Huang X., Alonso A., Guo X., Umbach D.M., Lichtenstein M.L., Ballantyne C.M., Mailman R.B., Mosley T.H., Chen H. (2015). Statins, plasma cholesterol, and risk of Parkinson’s disease: A prospective study. Mov. Disord..

[412] Jeong S.H., Lee H.S., Chung S.J., Yoo H.S., Jung J.H., Baik K., Lee Y.H., Sohn Y.H., Lee P.H. (2021). Effects of statins on dopamine loss and prognosis in Parkinson’s disease. Brain.

[413] Liu G., Sterling N.W., Kong L., Lewis M.M., Mailman R.B., Chen H., Leslie D., Huang X. (2017). Statins may facilitate Parkinson’s disease: Insight gained from a large, national claims database. Mov. Disord..

[414] Yan J., Qiao L., Tian J., Liu A., Wu J., Huang J., Shen M., Lai X. (2019). Effect of statins on Parkinson’s disease: A systematic review and meta-analysis. Medicine.

[415] Kaur D., Sharma V., Deshmukh R. (2019). Activation of microglia and astrocytes: A roadway to neuroinflammation and Alzheimer’s disease. Inflammopharmacology.

[416] Mendiola A.S., Cardona A.E. (2018). The IL-1beta phenomena in neuroinflammatory diseases. J. Neural. Transm..

[417] Luciunaite A., McManus R.M., Jankunec M., Racz I., Dansokho C., Dalgediene I., Schwartz S., Brosseron F., Heneka M.T. (2020). Soluble Abeta oligomers and protofibrils induce NLRP3 inflammasome activation in microglia. J. Neurochem..

[418] Pike A.F., Varanita T., Herrebout M.A.C., Plug B.C., Kole J., Musters R.J.P., Teunissen C.E., Hoozemans J.J.M., Bubacco L., Veerhuis R. (2021). alpha-Synuclein evokes NLRP3 inflammasome-mediated IL-1beta secretion from primary human microglia. Glia.

[419] Gonzalez P.V., Schioth H.B., Lasaga M., Scimonelli T.N. (2009). Memory impairment induced by IL-1beta is reversed by alpha-MSH through central melanocortin-4 receptors. Brain Behav. Immun..

[420] Kitazawa M., Cheng D., Tsukamoto M.R., Koike M.A., Wes P.D., Vasilevko V., Cribbs D.H., LaFerla F.M. (2011). Blocking IL-1 signaling rescues cognition, attenuates tau pathology, and restores neuronal beta-catenin pathway function in an Alzheimer’s disease model. J. Immunol..

[421] Long-Smith C.M., Collins L., Toulouse A., Sullivan A.M., Nolan Y.M. (2010). Interleukin-1beta contributes to dopaminergic neuronal death induced by lipopolysaccharide-stimulated rat glia in vitro. J. Neuroimmunol..

[422] Chakraborty A., Tannenbaum S., Rordorf C., Lowe P.J., Floch D., Gram H., Roy S. (2012). Pharmacokinetic and pharmacodynamic properties of canakinumab, a human anti-interleukin-1beta monoclonal antibody. Clin. Pharmacokinet..

[423] Ferrari F., Moretti A., Villa R.F. (2022). Incretin-based drugs as potential therapy for neurodegenerative diseases: Current status and perspectives. Pharmacol. Ther..

[424] De Iuliis A., Montinaro E., Fatati G., Plebani M., Colosimo C. (2022). Diabetes mellitus and Parkinson’s disease: Dangerous liaisons between insulin and dopamine. Neural Regen. Res..

[425] Fiory F., Perruolo G., Cimmino I., Cabaro S., Pignalosa F.C., Miele C., Beguinot F., Formisano P., Oriente F. (2019). The Relevance of Insulin Action in the Dopaminergic System. Front. Neurosci..

[426] Fine J.M., Stroebel B.M., Faltesek K.A., Terai K., Haase L., Knutzen K.E., Kosyakovsky J., Bowe T.J., Fuller A.K., Frey W.H. (2020). Intranasal delivery of low-dose insulin ameliorates motor dysfunction and dopaminergic cell death in a 6-OHDA rat model of Parkinson’s Disease. Neurosci. Lett..

[427] Iravanpour F., Dargahi L., Rezaei M., Haghani M., Heidari R., Valian N., Ahmadiani A. (2021). Intranasal insulin improves mitochondrial function and attenuates motor deficits in a rat 6-OHDA model of Parkinson’s disease. CNS Neurosci. Ther..

[428] Galustian C., Dalgleish A. (2009). Lenalidomide: A novel anticancer drug with multiple modalities. Expert. Opin. Pharmacother..

[429] Zhu Y.X., Kortuem K.M., Stewart A.K. (2013). Molecular mechanism of action of immune-modulatory drugs thalidomide, lenalidomide and pomalidomide in multiple myeloma. Leuk. Lymphoma.

[430] Valera E., Mante M., Anderson S., Rockenstein E., Masliah E. (2015). Lenalidomide reduces microglial activation and behavioral deficits in a transgenic model of Parkinson’s disease. J. Neuroinflamm..

[431] Cankara F.N., Gunaydin C., Bilge S.S., Ozmen O., Kortholt A. (2020). The neuroprotective action of lenalidomide on rotenone model of Parkinson’s Disease: Neurotrophic and supportive actions in the substantia nigra pars compacta. Neurosci. Lett..

[432] Li L., Sun R., Zenga J., Himburg H., Wang L., Duan S., Liu J., Bui D., Xie Z., Du T. (2022). Comparison of Absolute Expression and Turnover Number of COX-1 and COX-2 in Human and Rodent Cells and Tissues. J. Inflamm. Res..

[433] Kadusevicius E. (2021). Novel Applications of NSAIDs: Insight and Future Perspectives in Cardiovascular, Neurodegenerative, Diabetes and Cancer Disease Therapy. Int. J. Mol. Sci..

[434] Zaminelli T., Gradowski R.W., Bassani T.B., Barbiero J.K., Santiago R.M., Maria-Ferreira D., Baggio C.H., Vital M.A. (2014). Antidepressant and antioxidative effect of Ibuprofen in the rotenone model of Parkinson’s disease. Neurotox. Res..

[435] Hain E.G., Sparenberg M., Rasinska J., Klein C., Akyuz L., Steiner B. (2018). Indomethacin promotes survival of new neurons in the adult murine hippocampus accompanied by anti-inflammatory effects following MPTP-induced dopamine depletion. J. Neuroinflamm..

[436] Chen H., Zhang S.M., Hernan M.A., Schwarzschild M.A., Willett W.C., Colditz G.A., Speizer F.E., Ascherio A. (2003). Nonsteroidal anti-inflammatory drugs and the risk of Parkinson disease. Arch. Neurol..

[437] Szekely C.A., Thorne J.E., Zandi P.P., Ek M., Messias E., Breitner J.C., Goodman S.N. (2004). Nonsteroidal anti-inflammatory drugs for the prevention of Alzheimer’s disease: A systematic review. Neuroepidemiology.

[438] Poly T.N., Islam M.M.R., Yang H.C., Li Y.J. (2019). Non-steroidal anti-inflammatory drugs and risk of Parkinson’s disease in the elderly population: A meta-analysis. Eur. J. Clin. Pharmacol..

[439] Kothari V., Galdo J.A., Mathews S.T. (2016). Hypoglycemic agents and potential anti-inflammatory activity. J. Inflamm. Res..

[440] Zhang G., Lin X., Zhang S., Xiu H., Pan C., Cui W. (2017). A Protective Role of Glibenclamide in Inflammation-Associated Injury. Mediat. Inflamm..

[441] Abdelkader N.F., Farid H.A., Youness E.R., Abdel-Salam O.M.E., Zaki H.F. (2020). The role of K(ATP) channel blockade and activation in the protection against neurodegeneration in the rotenone model of Parkinson’s disease. Life Sci..

[442] Ishola I.O., Akataobi O.E., Alade A.A., Adeyemi O.O. (2019). Glimepiride prevents paraquat-induced Parkinsonism in mice: Involvement of oxidative stress and neuroinflammation. Fundam. Clin. Pharmacol..

[443] Qiu X., Wang Q., Hou L., Zhang C., Wang Q., Zhao X. (2021). Inhibition of NLRP3 inflammasome by glibenclamide attenuated dopaminergic neurodegeneration and motor deficits in paraquat and maneb-induced mouse Parkinson’s disease model. Toxicol. Lett..

[444] Landreth G., Jiang Q., Mandrekar S., Heneka M. (2008). PPARgamma agonists as therapeutics for the treatment of Alzheimer’s disease. Neurotherapeutics.

[445] Jankowska A., Wesolowska A., Pawlowski M., Chlon-Rzepa G. (2020). Diabetic Theory in Anti-Alzheimer’s Drug Research and Development—Part 1: Therapeutic Potential of Antidiabetic Agents. Curr. Med. Chem..

[446] Barbiero J.K., Santiago R.M., Persike D.S., da Silva Fernandes M.J., Tonin F.S., da Cunha C., Lucio Boschen S., Lima M.M., Vital M.A. (2014). Neuroprotective effects of peroxisome proliferator-activated receptor alpha and gamma agonists in model of parkinsonism induced by intranigral 1-methyl-4-phenyl-1,2,3,6-tetrahyropyridine. Behav. Brain Res..

[447] Breidert T., Callebert J., Heneka M.T., Landreth G., Launay J.M., Hirsch E.C. (2002). Protective action of the peroxisome proliferator-activated receptor-gamma agonist pioglitazone in a mouse model of Parkinson’s disease. J. Neurochem..

[448] Carta A.R., Frau L., Pisanu A., Wardas J., Spiga S., Carboni E. (2011). Rosiglitazone decreases peroxisome proliferator receptor-gamma levels in microglia and inhibits TNF-alpha production: New evidences on neuroprotection in a progressive Parkinson’s disease model. Neuroscience.

[449] Pinto M., Nissanka N., Peralta S., Brambilla R., Diaz F., Moraes C.T. (2016). Pioglitazone ameliorates the phenotype of a novel Parkinson’s disease mouse model by reducing neuroinflammation. Mol. Neurodegener..

[450] Schintu N., Frau L., Ibba M., Caboni P., Garau A., Carboni E., Carta A.R. (2009). PPAR-gamma-mediated neuroprotection in a chronic mouse model of Parkinson’s disease. Eur. J. Neurosci..

[451] Martin H.L., Mounsey R.B., Mustafa S., Sathe K., Teismann P. (2012). Pharmacological manipulation of peroxisome proliferator-activated receptor gamma (PPARgamma) reveals a role for anti-oxidant protection in a model of Parkinson’s disease. Exp. Neurol..

[452] Wang Y., Zhao W., Li G., Chen J., Guan X., Chen X., Guan Z. (2017). Neuroprotective Effect and Mechanism of Thiazolidinedione on Dopaminergic Neurons In Vivo and In Vitro in Parkinson’s Disease. PPAR Res..

[453] Lee E.Y., Lee J.E., Park J.H., Shin I.C., Koh H.C. (2012). Rosiglitazone, a PPAR-gamma agonist, protects against striatal dopaminergic neurodegeneration induced by 6-OHDA lesions in the substantia nigra of rats. Toxicol. Lett..

[454] Machado M.M.F., Bassani T.B., Coppola-Segovia V., Moura E.L.R., Zanata S.M., Andreatini R., Vital M. (2019). PPAR-gamma agonist pioglitazone reduces microglial proliferation and NF-kappaB activation in the substantia nigra in the 6-hydroxydopamine model of Parkinson’s disease. Pharmacol. Rep..

[455] NINDS Exploratory Trials in Parkinson Disease (NET-PD) FS-ZONE Investigators (2015). Pioglitazone in early Parkinson’s disease: A phase 2, multicentre, double-blind, randomised trial. Lancet Neurol..

[456] Hussain S., Singh A., Baxi H., Taylor B., Burgess J., Antony B. (2020). Thiazolidinedione use is associated with reduced risk of Parkinson’s disease in patients with diabetes: A meta-analysis of real-world evidence. Neurol. Sci..

[457] Schenk D.B., Koller M., Ness D.K., Griffith S.G., Grundman M., Zago W., Soto J., Atiee G., Ostrowitzki S., Kinney G.G. (2017). First-in-human assessment of PRX002, an anti-alpha-synuclein monoclonal antibody, in healthy volunteers. Mov. Disord..

[458] Jankovic J., Goodman I., Safirstein B., Marmon T.K., Schenk D.B., Koller M., Zago W., Ness D.K., Griffith S.G., Grundman M. (2018). Safety and Tolerability of Multiple Ascending Doses of PRX002/RG7935, an Anti-alpha-Synuclein Monoclonal Antibody, in Patients With Parkinson Disease: A Randomized Clinical Trial. JAMA Neurol..

[459] Katila N., Bhurtel S., Shadfar S., Srivastav S., Neupane S., Ojha U., Jeong G.S., Choi D.Y. (2017). Metformin lowers alpha-synuclein phosphorylation and upregulates neurotrophic factor in the MPTP mouse model of Parkinson’s disease. Neuropharmacology.

[460] Lu M., Su C., Qiao C., Bian Y., Ding J., Hu G. (2016). Metformin Prevents Dopaminergic Neuron Death in MPTP/P-Induced Mouse Model of Parkinson’s Disease via Autophagy and Mitochondrial ROS Clearance. Int. J. Neuropsychopharmacol..

[461] Wang D.X., Chen A.D., Wang Q.J., Xin Y.Y., Yin J., Jing Y.H. (2020). Protective effect of metformin against rotenone-induced parkinsonism in mice. Toxicol. Mech. Methods.

[462] Katila N., Bhurtel S., Park P.H., Choi D.Y. (2021). Metformin attenuates rotenone-induced oxidative stress and mitochondrial damage via the AKT/Nrf2 pathway. Neurochem. Int..

[463] Saewanee N., Praputpittaya T., Malaiwong N., Chalorak P., Meemon K. (2021). Neuroprotective effect of metformin on dopaminergic neurodegeneration and alpha-synuclein aggregation in C. elegans model of Parkinson’s disease. Neurosci. Res..

[464] Tayara K., Espinosa-Oliva A.M., Garcia-Dominguez I., Ismaiel A.A., Boza-Serrano A., Deierborg T., Machado A., Herrera A.J., Venero J.L., de Pablos R.M. (2018). Divergent Effects of Metformin on an Inflammatory Model of Parkinson’s Disease. Front. Cell. Neurosci..

[465] Chen Y., Hamidu S., Yang X., Yan Y., Wang Q., Li L., Oduro P.K., Li Y. (2022). Dietary Supplements and Natural Products: An Update on Their Clinical Effectiveness and Molecular Mechanisms of Action During Accelerated Biological Aging. Front. Genet..

[466] Aviles-Olmos I., Dickson J., Kefalopoulou Z., Djamshidian A., Ell P., Soderlund T., Whitton P., Wyse R., Isaacs T., Lees A. (2013). Exenatide and the treatment of patients with Parkinson’s disease. J. Clin. Investig..

[467] Athauda D., Gulyani S., Karnati H.K., Li Y., Tweedie D., Mustapic M., Chawla S., Chowdhury K., Skene S.S., Greig N.H. (2019). Utility of Neuronal-Derived Exosomes to Examine Molecular Mechanisms That Affect Motor Function in Patients with Parkinson Disease: A Secondary Analysis of the Exenatide-PD Trial. JAMA Neurol..

[468] Fu X., Wang Y., He X., Li H., Liu H., Zhang X. (2020). A systematic review and meta-analysis of serum cholesterol and triglyceride levels in patients with Parkinson’s disease. Lipids Health Dis..

[469] Rozani V., Gurevich T., Giladi N., El-Ad B., Tsamir J., Hemo B., Peretz C. (2018). Higher serum cholesterol and decreased Parkinson’s disease risk: A statin-free cohort study. Mov. Disord..

[470] Pike C.J. (2001). Testosterone attenuates beta-amyloid toxicity in cultured hippocampal neurons. Brain Res..

[471] Fine J.M., Kosyakovsky J., Baillargeon A.M., Tokarev J.V., Cooner J.M., Svitak A.L., Faltesek K.A., Frey W.H., Hanson L.R. (2020). Intranasal deferoxamine can improve memory in healthy C57 mice, suggesting a partially non-disease-specific pathway of functional neurologic improvement. Brain Behav..

[472] Novak P., Pimentel Maldonado D.A., Novak V. (2019). Safety and preliminary efficacy of intranasal insulin for cognitive impairment in Parkinson disease and multiple system atrophy: A double-blinded placebo-controlled pilot study. PLoS ONE.

[473] Teismann P., Ferger B. (2001). Inhibition of the cyclooxygenase isoenzymes COX-1 and COX-2 provide neuroprotection in the MPTP-mouse model of Parkinson’s disease. Synapse.

[474] Kurkowska-Jastrzebska I., Babiuch M., Joniec I., Przybylkowski A., Czlonkowski A., Czlonkowska A. (2002). Indomethacin protects against neurodegeneration caused by MPTP intoxication in mice. Int. Immunopharmacol..

[475] Chen H., Jacobs E., Schwarzschild M.A., McCullough M.L., Calle E.E., Thun M.J., Ascherio A. (2005). Nonsteroidal antiinflammatory drug use and the risk for Parkinson’s disease. Ann. Neurol..

[476] Piri H., Haghdoost-Yazdi H., Fraidouni N., Dargahi T., Yaghoubidoust M., Azadmehr A. (2017). The Anti-Parkinsonism Effects of K(ATP) Channel Blockade in the 6-Hydroxydopamine-Induced Animal Model: The Role of Oxidative Stress. Basic Clin. Neurosci..

[477] Blackburn J.K., Curry D.W., Thomsen A.N., Roth R.H., Elsworth J.D. (2020). Pioglitazone activates paraoxonase-2 in the brain: A novel neuroprotective mechanism. Exp. Neurol..

[478] Pisanu A., Lecca D., Mulas G., Wardas J., Simbula G., Spiga S., Carta A.R. (2014). Dynamic changes in pro- and anti-inflammatory cytokines in microglia after PPAR-gamma agonist neuroprotective treatment in the MPTPp mouse model of progressive Parkinson’s disease. Neurobiol. Dis..

[479] Costa H.N., Esteves A.R., Empadinhas N., Cardoso S.M. (2023). Parkinson’s Disease: A Multisystem Disorder. Neurosci. Bull..

[480] Picca A., Guerra F., Calvani R., Romano R., Coelho-Junior H.J., Bucci C., Marzetti E. (2021). Mitochondrial Dysfunction, Protein Misfolding and Neuroinflammation in Parkinson’s Disease: Roads to Biomarker Discovery. Biomolecules.

[481] Li T., Le W. (2020). Biomarkers for Parkinson’s Disease: How Good Are They?. Neurosci. Bull..

[482] Schirinzi T., Di Lazzaro G., Sancesario G.M., Summa S., Petrucci S., Colona V.L., Bernardini S., Pierantozzi M., Stefani A., Mercuri N.B. (2020). Young-onset and late-onset Parkinson’s disease exhibit a different profile of fluid biomarkers and clinical features. Neurobiol. Aging.

[483] Srikanth V., Westcott B., Forbes J., Phan T.G., Beare R., Venn A., Pearson S., Greenaway T., Parameswaran V., Munch G. (2013). Methylglyoxal, cognitive function and cerebral atrophy in older people. J. Gerontol. A Biol. Sci. Med. Sci..

